# Exploring glycopeptide-resistance in *Staphylococcus aureus*: a combined proteomics and transcriptomics approach for the identification of resistance-related markers

**DOI:** 10.1186/1471-2164-7-296

**Published:** 2006-11-22

**Authors:** Alexander Scherl, Patrice François, Yvan Charbonnier, Jacques M Deshusses, Thibaud Koessler, Antoine Huyghe, Manuela Bento, Jianru Stahl-Zeng, Adrien Fischer, Alexandre Masselot, Alireza Vaezzadeh, Francesca Gallé, Adriana Renzoni, Pierre Vaudaux, Daniel Lew, Catherine G Zimmermann-Ivol, Pierre-Alain Binz, Jean-Charles Sanchez, Denis F Hochstrasser, Jacques Schrenzel

**Affiliations:** 1 Biomedical Proteomics Research Group, Geneva University, Geneva, Switzerland; 2 Service of infectious diseases, University Hospitals of Geneva, Geneva, Switzerland; 3 Applied Biosystems, Darmstadt, Germany; 4 GeneBio SA, Geneva, Switzerland; 5 Central Clinical Chemistry Laboratory, University Hospitals of Geneva, Geneva Switzerland; 6 Pharmacy section, Faculty of Sciences, Geneva University, Switzerland

## Abstract

**Background:**

To unravel molecular targets involved in glycopeptide resistance, three isogenic strains of *Staphylococcus aureus *with different susceptibility levels to vancomycin or teicoplanin were subjected to whole-genome microarray-based transcription and quantitative proteomic profiling. Quantitative proteomics performed on membrane extracts showed exquisite inter-experimental reproducibility permitting the identification and relative quantification of >30% of the predicted *S. aureus *proteome.

**Results:**

In the absence of antibiotic selection pressure, comparison of stable resistant and susceptible strains revealed 94 differentially expressed genes and 178 proteins. As expected, only partial correlation was obtained between transcriptomic and proteomic results during stationary-phase. Application of massively parallel methods identified one third of the complete proteome, a majority of which was only predicted based on genome sequencing, but never identified to date. Several over-expressed genes represent previously reported targets, while series of genes and proteins possibly involved in the glycopeptide resistance mechanism were discovered here, including regulators, global regulator attenuator, hyper-mutability factor or hypothetical proteins. Gene expression of these markers was confirmed in a collection of genetically unrelated strains showing altered susceptibility to glycopeptides.

**Conclusion:**

Our proteome and transcriptome analyses have been performed during stationary-phase of growth on isogenic strains showing susceptibility or intermediate level of resistance against glycopeptides. Altered susceptibility had emerged spontaneously after infection with a sensitive parental strain, thus not selected *in vitro*. This combined analysis allows the identification of hundreds of proteins considered, so far as hypothetical protein. In addition, this study provides not only a global picture of transcription and expression adaptations during a complex antibiotic resistance mechanism but also unravels potential drug targets or markers that are constitutively expressed by resistant strains regardless of their genetic background, amenable to be used as diagnostic targets.

## Background

The Gram-positive bacterium *Staphylococcus aureus *is an important human pathogen that has become increasingly resistant to a wide range of antibiotics over the last two decades. The emergence of multidrug-resistant isolates of methicillin-resistant *S. aureus *(MRSA) exhibiting also decreased susceptibilities to glycopeptides (glycopeptide-intermediate *S. aureus*, GISA) represents a crucial challenge for antimicrobial therapy, antimicrobial susceptibility testing, and hospital infection control. After initial description in Japan of MRSA strains with decreased susceptibility to glycopeptides [1], clinical isolates showing similar phenotypes were repeatedly reported in various countries [2-6]. These strains are distinct from high-level glycopeptide resistant isolates (VRSA) that result from the acquisition of the *vanA *gene from *Enterococcus faecalis *[7]. Their potential spreading appears of particular concern since glycopeptides represent the last barrier drugs effective against MRSA. In addition, intensive use of glycopeptides will probably contribute to the selection of other resistant strains, as already observed for numerous antimicrobial agents [8].

Vancomycin is a natural product, isolated from the bacteria *Amycolatopsis orientalis *in the early fifties [9]. Binding of this molecule to the N-acyl D-ala-D-ala residue of bacterial peptidoglycan through five strong hydrogen bonds inhibits cross-linking of the cell-wall [10]. Vancomycin acts therefore immediately upstream of the transpeptidase, the target of β-lactam antibiotics. Structure and mechanism of action of teicoplanin are similar to that of vancomycin [10]. Little is known about the underlying mechanisms which produce GISA strains. A major common marker of GISA strains is the increased cell-wall thickness [11]: the prototype GISA strain shows 30–40 cross-linked peptidoglycan layers, whereas fully susceptible strains contain only 20 layers [12].

Approximately 20% of free D-ala-D-ala residues from the peptidoglycan structure remain unprocessed by penicillin-binding proteins (PBPs) in vancomycin-susceptible strains. It was therefore suggested that important quantities of glycopeptides are trapped by these free residues [12]. In addition, it was shown that the cross-linking rate is slower in laboratory-derived GISA compared to susceptible strains [13,14], which may increase the number of trapped vancomycin molecules and contribute to the destruction of the mesh-structure of the cell-wall [15]. This cooperative clogging phenomenon has been recently shown to prevent vancomycin from reaching its target in the cytoplasmic membrane of strain Mu50, the first clinical isolate reported as GISA [16]. However, analysis of the digested cell-wall compounds with high-performance liquid chromatography (HPLC) showed different peptidoglycan structures among various clinical isolates of GISA strains showing various cross-linking frequency [17]. In other backgrounds, originating from *in vitro *selection of laboratory strains, structural changes in cell-wall composition were limited [18] suggesting that there is no single genetic or biochemical change responsible for the decreased glycopeptide sensitivity. Several genes were indeed described to play a role in glycopeptide resistance. Penicillin-binding protein 4 (PBP4) is hypothesized to cleave the terminal D-alanine residue from un-cross-linked peptidoglycan chains [19]. A decrease in PBP4 activity, as observed in clinical isolates of GISA strains, should therefore result in a higher number of D-ala-D-ala targets for vancomycin [19]. PBP2 over-expression seems also correlated with decreased glycopeptide susceptibility [20,21]. The loss of function of the accessory gene regulator (*agr*) also increases resistance to glycopeptide [22]. A recent report demonstrated the contribution of *tcaA *in the resistance mechanism. This gene encodes for a transmembrane protein showing an intracellular metal-binding motif and a large extracellular domain of unknown function. While inactivation of this gene induces increased tolerance to glycopeptides, its presence induces overproduction of the same protein, suggesting that this protein acts as a sensor and/or as a signal transducer [23]. However, the impact of such an observation on clinical isolates is still debated [24]. The two-component system *vraRS *[25] has been shown to be involved in glycopeptide resistance. The expression of *vraRS *probably mediates positive regulation of cell-wall synthesis pathway in *S. aureus*, through induction of *pbp2 *[21], thus increasing the sensitivity of GISA strains to cell-wall synthesis inhibitors [25]. In another study performed with clinical isolates and Mu50 or Mu50 derivative strains showing increased levels of resistance after passages onto vancomycin-containing medium, most of the genes involved in purine biosynthesis and transport were found up-regulated in the highly vancomycin-resistant strain as compared to the parental strain [26]. The deduced hypothesis relies on an increase in AMP pool able to generate ATP and compensate the difference in energy requirement observed between susceptible and resistant isolates.

Altogether, glycopeptide-resistance in *Staphylococcus aureus *appears due to multiple factors, including cell-wall synthesis and processing [27], autolysis [28,29], or regulatory events [23,24,30]. A major limitation of this type of comparative study is the difficulty to obtain isogenic strains showing various susceptibility phenotypes within the same genetic background and relevance in the context of human infections. Our group reported the isolation of GISA subpopulations emerging from a glycopeptide-susceptible parental strain in an experimental model of subcutaneous infection [31]. In the same model of infection, we then isolated a spontaneous revertant strain showing restored susceptibility to glycopeptides [32]. To improve our understanding of mechanisms contributing to this spontaneous evolution, these three isogenic strains presenting different glycopeptide susceptibility levels were compared at the transcriptome and proteome levels during the stationary phase of growth. Transcriptional profiles were performed using a customized and extensively validated oligoarray [33]. In addition, mass spectrometry (MS)-based protein quantification was performed on membrane-enriched extracts. Enriched membrane extracts were chosen for proteomic experiments, since membrane proteins are often involved in bacterial antibiotic resistance and contribute also to numerous important metabolic pathways and transports, not only of nutrients but also of chemicals [34-38]. The recently introduced isobaric tagging technology was used for simultaneous quantification in all strains [39,40]. Isoelectric focusing (IEF) on immobilized pH gradients served as the first dimensional peptide separation [41], followed by LC-MS/MS analysis with a MALDI tandem MS instrument. These combined approaches performed on isogenic clinical isolates showing stable resistance, without antibiotic pressure revealed genes and proteins identified as potentially involved in the acquisition of glycopeptide resistance through the involvement of a complex and multi-factorial biological network. In addition, most of genes or proteins previously suspected to contribute to glycopeptide resistance were identified in our study, confirming the robustness of our approach. Finally, six potential targets found up-regulated at the transcript and protein levels are also shown to be over-expressed in a collection of *S. aureus *clinical isolates from unrelated genetic background showing reduced susceptibility to glycopeptides.

## Results

### Clonality of the studied strains

MLST performed with partial sequences of 7 genes revealed that strains MRGR3, 14-4 and 14-4Rev displayed the same allele profile 2.3.1.1.4.4.3 for *arcC*, *aroE*, *glpf*, *gmk*, *pta*, *tpi*, *yqiL*, respectively, corresponding to MLST 239.

To further confirm the clonality of these strains, comparative genome hybridization was performed on an oligoarray covering the whole genome of *Staphylococcus aureus *COL, N315, Mu50 and MW2. Additional file 1 illustrates that susceptible strain MRGR3 and the GISA strain 14-4 displayed strictly identical fluorescence patterns on the 5427 unique oligonucleotide probes [33].

### Proteomic experiments

**Figure 1:**
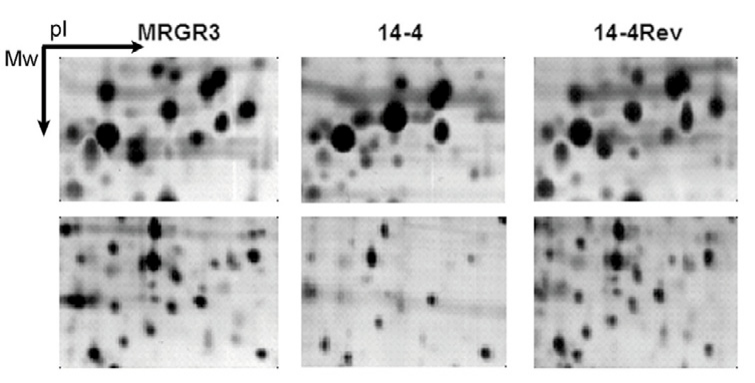
Two windows for each strain are represented to illustrate 2-D gel electrophoresis performed on membrane-enriched protein fractions of GISA (14-4) and glycopeptide-susceptible strains (MRGR3 and 14-4Rev). While similar patterns are observed between susceptible strains, drastic differences are visible when comparing the GISA with either of the susceptible strains.

Analysis by 2-DE revealed striking similarities in protein expression of the two glycopeptide-susceptible strains (MRGR3 and 14-4Rev). On the opposite, the protein pattern between the glycopeptide intermediate strain (14-4) and both susceptible strains appeared totally different (Figure 1). To document this high variability, quantitative proteomic experiments were performed on membrane-enriched fractions with MS-based approaches, using isobaric tags. Isoelectric focusing of peptides was used as first dimension separation, prior to LC-MS/MS analysis. Enrichment was evaluated by assaying the lactate dehydrogenase activity, a marker of cytosolic contents. Enzymatic activity reached 1330 U/g in total protein extract and 60 U/g in membrane protein.

All quantitative proteomics experiments were performed in duplicates. In the first experiment (PR1), 3'724 unique peptides corresponding to 632 proteins were identified from the bacterial membrane fraction. In the second experiment (PR2), 3'719 peptides corresponding to 754 proteins were identified. A total of 551 proteins were commonly identified in both experiments. Together, these experiments yielded a total of 835 unique proteins, covering approximately 32% of the whole deduced proteome of *S. aureus *strain N315 (see Additional files 2, 3, 4).

Among the 2'575 predicted ORFs of strain N315, 637 protein products (24%) are predicted to be integral membrane proteins (e.g. containing at least one transmembrane domain). Among the proteins identified during these two experiments, approximately 20% are predicted to be integral membrane proteins.

Intense signal from the reporter fragment ions (derived from the reporter fragment ions abundance at m/z 114.1, 115.1, 116.1 and 117.1 Da) was obtained for almost all peptides (>95%). Relative quantification could therefore be performed on all 835 proteins differentially expressed between strain MRGR3 and 14-4, on 826 proteins expressed between 14-4 and 14-4Rev, and on 826 proteins between MRGR3 and 14-4Rev. A list of these proteins is provided separately (see Additional files 5, 6). Tables contain the corresponding ORF number, their relative abundance ratio as well as the coefficient of variation (CV) of ratios from individual peptides belonging to the same protein. In average, 4.9 peptides per protein were used for quantification. As shown in additional tables, the CV of individual peptide ratios belonging to the same protein is relatively high. When comparing glycopeptide-susceptible strains, mean CV value is 23.5%, whereas it increased to 32.6% between 14-4 and 14-4Rev, and 36.5% between 14-4 and MRGR3. Most identified peptides matched those of the nucleotide sequence-deduced [42] peptides of strain N315. However, when peptides were not found in N315 genome, ORF numbers from other sequenced *S. aureus *strains are provided in the tables (see Additional files 5, 6).

**Figure 2:**
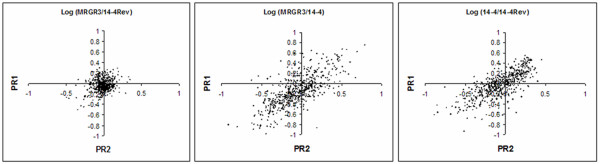
Dispersion of expression ratios obtained for two independent quantitative proteomic experiments illustrates the reproducibility of the whole procedure and the homogeneity observed when comparing the two susceptible strains (A). A good correlation but a large difference in protein expression is observed when comparing GISA and either MRGR3 or 14-4Rev (panels B and C with correlation coefficients of 0.92 and 0.93; respectively).

Scatter plots of the proteomics ratio revealed very similar profiles in both glycopeptide-susceptible strains (MRGR3 and 14-4Rev). The number of proteins out of the range -0.5 to 0.5 (log_10 _of protein expression ratio) was 0, and only 10 proteins appeared out of the range of -0.3 to 0.3 (Figure 2A). On the opposite, important modifications of the distribution were observed between the GISA strain and any of the 2 glycopeptide-susceptible ones (Figures 2B and 2C). The number of proteins found more abundant in one strain over the other was approximately 60 in the range of -0.5 to 0.5 and 160 in the range of -0.3 to 0.3, resulting in a scatter distribution instead of a 0-centered representation. The lists of identified proteins between the GISA strain and either of the susceptible strains revealed almost the same content as all but only 9 proteins were found in the two analyses, indicating excellent reproducibility of the membrane protein preparations. The experimental variability observed between quantifications of the two replicates was relatively important. Figures 2B and 2C depict several proteins with divergent expression ratios between replicates. Due to experimental variation, inherent to quantitative mass spectrometry techniques [43] and illustrated by the relative high CV values of individual peptides from the same protein, a conservative approach was selected that considered only proteins significantly differentially expressed in both experiments. To this end, we considered the distribution between both glycopeptide-susceptible strains (MRGR3 and 14-4Rev) as a Gaussian normal distribution. Threshold for significant differential expression was set at the 5^th ^percentile of the most over- or under-expressed proteins (2.5 percentile most over-expressed and 2.5 percentile most under-expressed) between both glycopeptide-susceptible strains (MRGR3 and 14-4Rev).

A total of 155 proteins are differentially expressed between strains 14-4 and MRGR3, and 110 proteins between strains 14-4 and 14-4Rev, which corresponds to approximately 4% of the deduced proteome. Together, 178 unique proteins are differentially expressed in the GISA strain. A list of these proteins is given in supplementary material (see Additional files 5 and 6). Comparison reveals that more than 65% of these proteins are common between both lists obtained from comparison of the resistant strain with either of the two susceptible isolates. Among these 2 lists, the most important categories involved proteins playing a role in: energy metabolism, amino-acids transport, cell envelope biosynthesis, protein turnover and inorganic ion transport (corresponding to COG categories C, E, M, O, P, respectively). Among the 19 categories represented, these 5 categories constituted 40% of the identified proteins.

### Transcriptomic analysis

Genes with statistically significant changes in the level of expression and differences superior to two-fold changes were identified and listed for pairwise comparisons (see additional files 7, 8). According to these criteria, only 10 genes were differentially regulated between strain MRGR3 and 14-4Rev, consisting in 0.4% of the transcriptome. However, strain 14-4 compared to MRGR3 showed 67 differentially regulated genes, whereas 14-4 compared to 14-4Rev showed 64. In total, 94 differentially expressed genes were identified between GISA and both glycopeptide-susceptible strains. Finally, these tables reveal that more than 65% of these genes were common. Among the up- or down-regulated genes, the most represented transcripts were found in COG E, K and P corresponding to genes involved in amino acids transport, transcription and inorganic ions transport, respectively. Thus, 2 out of these 3 categories were also found predominant in the quantitative proteome study.

### Correlation between transcriptomic and proteomic data

The transcriptomic expression trends compared to the proteomic quantification for all identified proteins are shown in Tables 1 and 2 for the comparison between the two sensitive strains and the GISA. This analysis reveals that an equivalent proportion of genes are regulated similarly in both comparisons.

**Figure 3:**
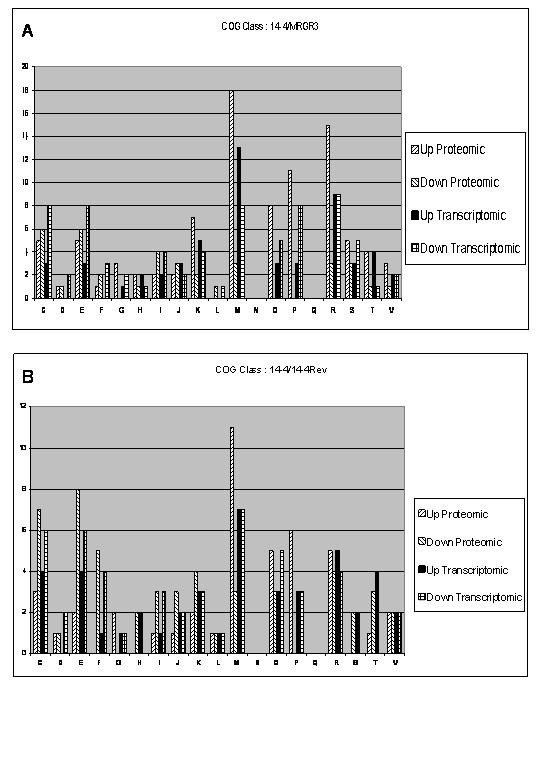
Differentially expressed proteins with the corresponding transcripts expression trend regrouped by functions for the comparison between GISA strain 14-4, and MRGR3 or 14-4Rev (A and B). Similar general profiles were obtained for the two comparisons between the GISA and either of the susceptible strains while totally different profile was observed for the two susceptible strains. Functional classes are: C = energy production and conversion; D = cell division and chromosome partitioning; E = amino acid transport and metabolism; F = nucleotide transport and metabolism; G = carbohydrate transport and metabolism; H = coenzyme metabolism; I = lipid metabolism; J = translation, ribosomal structure and biogenesis; K = transcription; L = DNA replication, recombination and repair; M = cell envelope biogenesis, outer membrane; N = cell motility; O = post-translational modification, protein turnover, chaperones; P = inorganic ion transport and metabolism; Q = secondary metabolites biosynthesis, transport and catabolism; R = general function prediction; S = function unknown; T = signal transduction mechanisms; U = secretion; V = defense mechanism. Number of proteins for each category is indicated on top of the corresponding columns.

The comparison between the proteomic expression direction (Tables 1 and 2) and the corresponding transcriptomic expression direction according to the functional classes (according to the COG database) shows important similarities for most categories. However, two functional classes (COG O and P, corresponding respectively to post-translational modification, protein turnover, chaperones and inorganic ion transport) show opposed quantification trends (Figure 3A and 3B).

Finally, strict comparison of significantly differentially expressed proteins and genes allowed us to identify 7 common ORFs (Tables 3 and 4) showing the same transcript and proteins expression trends. Among these genes, 5 are over-expressed whereas only two are under-expressed. In addition, SA0532 that revealed strongly up-regulated in microarray experiments was also evaluated in quantitative analysis.

### Genetic relationships between strains showing glycopeptide intermediate level of resistance and identified markers expression

**Figure 4:**
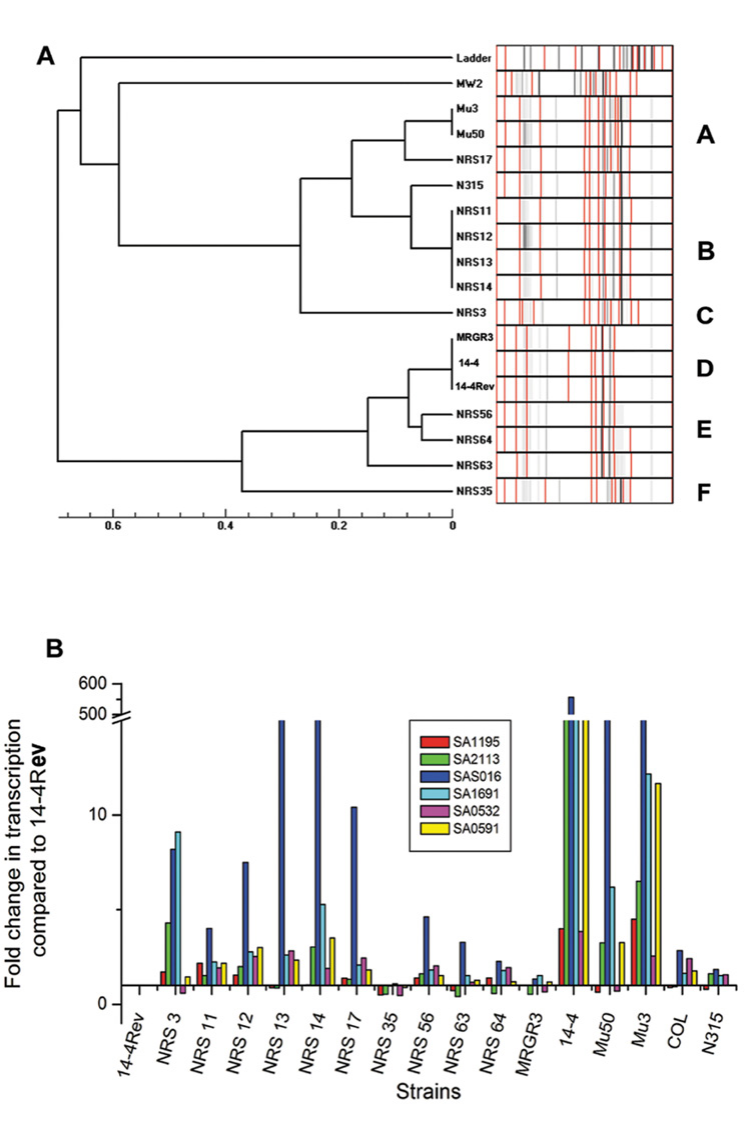
**A. **Genotyping of strains evaluated by VNTR. Based on the limit of clonality of the assay (0.1 unit of divergence) [77], our collection contains at least 4 unrelated genetic backgrounds (noted A-D). Control strains whose genome has been sequenced have been simultaneously analyzed. **B. **Gene expression of identified markers on a collection of strains showing altered susceptibility to glycopeptides. Relative expression of 6 gene markers found up-regulated in the resistant strain 14-4 compared to the two sensitive strains. The same markers were also quantified in a collection of strains from the NARSA, selected for their resistance to glycopeptide. Values are mean + standard deviation of triplicate measurements from 2 independent experiments. Increase expression of selected markers, linked to glycopeptide resistance is independent on the background. The complete raw microarray dataset has been posted on the Gene Expression Omnibus database [78], accession number GSE5188. All additional files are freely accessible [79].

The expression ratio of the five over-expressed ORFs at the gene and protein level in glycopeptide-intermediate strain 14-4 as well as the over-expressed transcript SA0532 was measured on a collection of genetically unrelated strains (Table 5). Rapid genotype clustering (Figure 4A) shows that our collection of strains displays at least five distinct genomic contents confirming that these strains are not genetically related. Cluster A composed of Mu50 and Mu3 shows some relatedness with NRS17, a strain isolated in the USA. This relatedness between the two Japanese isolates has been documented previously [44]. Cluster B contains the closely-related French isolates. Patterns C and F contain only a single isolate (NRS3 and NRS35, respectively). Cluster D confirms that our set of isogenic strains is unique, and appeared clonal which is in accordance with the MLST and CGH results (see Additional file 1), and partially related to cluster E. Finally, the 3 strains extensively documented in the present study appear clonal but clearly different from at least 4 other genotypes identified. As previously documented using pulse-field gel electrophoresis, our three strains appeared isogenic [45].

Expression levels of the 6 mRNA markers potentially involved in the mechanism of glycopeptide resistance, assessed by quantitative PCR using specific oligonucleotides (Table 6) is shown on Figure 4B. Important differences were observed between strains and their genetic backgrounds. All reference strains (e.g. N315, COL) showed marginal differences in terms of gene expression compared to other strains reported as glycopeptides-intermediate. The most variable markers were SA0536.1, SA1691 and SA0591, which revealed importantly up-regulated in the vast majority of unrelated GISA strains. A similar expression pattern of these markers was found in our resistant strain 14-4 as in the ancestral GISA prototype, Mu50 and its clonal derivative Mu3 showing important up-regulation of all identified markers. Taken together, these results illustrate the benefits of performing combined transcriptomic and proteomic analysis. Indeed, both were necessary to obtain a global and holistic view of the state of the bacterial cells. On the opposite, analysis restricted to gene-expression or protein abundance may lead to important bias.

## Discussion

### Technical issues and challenges

The use of isobaric-tagging during isoelectric focusing prior to LC-MS/MS provided the possibility to quantitatively examine a large number of proteins for differential expression [39,40]. Using membrane-enriched protein extracts from *Staphylococcus aureus *during stationary phase, we consistently identified and quantified relative protein abundance for approximately 32% of the proteome when comparing pairs of strains. Previously, we showed that only 23% of the proteome was accessible using different combinations of protein or peptide fractionation and numerous separation techniques [46]. The capacity to obtain extended proteome recovery, particularly from membrane fractions, represents one of the major bottlenecks in proteomic experiments. As expected from multiple previous reports, numerous soluble contaminants are identified from the membrane-enriched fractions. However, these compounds greatly enhance the number of identified proteins and contribute to the global picture of the proteome from a single cellular fraction. In addition, the removal of important fractions of abundant cytosolic proteins increases the number of low-abundance proteins linked to the bacterial membrane [46]. In this report, we concentrated on membrane-enriched fraction. Our membrane purification process allowed reducing drastically the content of cell-wall anchored and cytosolic proteins, which constitute the most abundant fraction of cellular proteins by a 200-fold factor. Based on the content of *Staphylococcus aureus *proteome, this corresponds to approximately 4% contamination of membrane fraction by cytosolic proteins Western blot performed with antibodies raised against *S. aureus *protein A showed that the contamination by cell-wall anchored proteins was marginal. The new approach with an IEF separation of peptides leads to the identification of an appreciable part of the bacterium proteome, maximizing the chances to discover new targets involved in *S. aureus *glycopeptide resistance. In this respect, our analysis of *S. aureus *proteome allowed satisfactory representation of low-abundance or membrane proteins. In this work, 19% of the identified proteins constituted integral membrane proteins, a value close to the predicted transmembrane proteome (19.7% of proteome showed at least 2 putative transmembrane domains) as deduced from the nucleotide sequence [42,46]. Numerous examples of bacterial resistance against different antibiotic families involve membrane proteins [34-37]. Membrane proteins are notoriously difficult to solubilize during tryptic digestion and, depending on the number of transmembrane domains, most of the fractionated peptides remain highly hydrophobic which is an important limitation during isoelectric focusing [47]. Due to important differences between the three strains analyzed in this study, this aspect is of utmost importance. The mean CV of individual peptide ratios belonging to the same protein varies between 23.5% (ratios between MRGR3 and 14-4Rev) and 36.5% (ratios between 14-4 and susceptible strains) which is relatively low, based on the complexity of the whole experimental procedure. The sample variability certainly contributed to these variations, as we observed higher CVs for the comparison between susceptible and GISA strains. The high peptide recovery due to combination of IEF, LC-MS/MS and labeling strategy is therefore essential for reliable protein quantification. Similar quantitative profiles were obtained in each individual experiment, as shown on Figure 2. However, totally different profiles were observed between either of the two susceptible strains and the GISA (Figures 2A and 2B). This observation is concordant with 2D-gel electrophoresis results which clearly illustrates divergent patterns between GISA and the susceptible strains.

Whereas high-level glycopeptide resistance in *S. aureus *has been shown to rely on the horizontal transfer of *vanA *gene from *Enterococcus faecalis *[7,48], the mechanisms involved in glycopeptide-intermediate resistance remain poorly understood and numerous potential mechanisms are currently explored [11,27]. They probably involve a complex and global gene expression and protein translation changes as well as protein processing alterations. The most documented modifications contributing to this phenotype involve profound modifications of cell-wall biosynthesis including increased cell-wall thickness, cross-linking, or accumulation of cell-wall components [11,27]. In this study, several targets involved in cell-wall synthesis were found differentially expressed either at the protein or transcript level, or at both levels. Other potential hypothesis rely on the increased abundance of D-Ala-D-Ala residues, forming false-target trapping glycopeptides or to an altered regulation of cell-wall synthesis [12,14]. Other potential mechanisms are related to the requirement of increased energy synthesis related to larger cell-wall contents, involving phosphoglycerate kinase [49], or purine metabolism [26].

In a cell, protein abundance is not strictly correlated to that of its cognate mRNA levels. Thus, a combined transcriptomics and proteomics approach is warranted for genome-wide identification of molecular targets involved in the resistance mechanism. Previous experiments performed in our laboratory showed that resistance to glycopeptides expressed by strain 14-4 remains stable even after numerous sub-cultures in antibiotic-free medium [31]. This resistant phenotype relies on constitutive changes in gene expression and is stable, thus not requiring drug induction. At stationary phase, a decoupling between protein and transcripts abundance is generally observed except for most of constitutive processes [50], supporting our exploration of the transcriptome and proteome of *S. aureus *during this stage.

### Differentially expressed targets in GISA compared to susceptible strains

Proteomics as well as transcriptomics studies revealed moderate numbers of differentially regulated genes or proteins. Comparison of GISA strain with either of the 2 susceptible isolates showed a majority of common differentially expressed targets. In addition, a perfect correlation was observed for those common genes in the transcriptomic evaluation and the proteomic studies. Genes or gene products known to be involved in different antibiotic resistance mechanisms, such as PBP2 (SA1283/Q7A5K8), Ant (SA2385/P04827), MurE (SA0876/P65480), and the methicillin resistance-related autolysis protein FmtA (SA0909/Q7A6A2) were over-expressed in the GISA strain. In addition to these targets involved in cell-wall biosynthesis, many other proteins involved in cell-wall metabolism (formation or post-processing and hydrolysis) were found over-expressed, such as SgtB (SA1691/Q7A4S6) and SsaA (SA2093/Q7A423). Part of these compounds involved in cell-wall processing was reported to alter glycopeptide susceptibility [27]. These results were shown in gene expression study [27] and are now confirmed on protein expression levels.

Another important protein target found differentially expressed at the mRNA and protein levels is the product of *vraX *gene (SA0536.1/Q99W32, accession number AB050664). This gene belongs to the *vra *operon (SA0533–SA0535) encoding for genes initially described to be involved in imipenem resistance. Most of these genes were up-regulated at the mRNA levels together with SA0532 and SA0536.1, located upstream and downstream of the *vra *region, respectively and suggesting contribution in glycopeptide resistance. Motive search revealed that both genes, SA0532 and SA0536 harbored a putative phosphorylation site, suggesting a role in a regulatory process. Regulatory systems contributed to the GISA phenotype, such as the two-component sensor histidine kinase, coded by the *vraSR *genes (SA1701-SA1700), previously shown as important in the development of resistance to either imipenem or glycopeptides [25]. Our study reveals also that protein regulators are over-expressed, such as the signal transduction protein TRAP (Q7A4W3/SA1653) [51], acting as a signal-transducer protein during quorum sensing [51,52], the divIVA protein (SA1279/Q7A5L1) known to regulate cell division, or the putative transcription factor (SA2296/Q7A3J2) and *sarH1*, whose up-regulation is in accordance with a decreased expression of *spa*. Finally, the over-expression of proteins involved in stress protection such as proteinases CtpA (SA1253/Q7A5M9), MsrA and MsrB (SA1257/P99065 and SA1256/P65446) and the regulator MsrR (SA1195/Q99Q02), contributing to the reparation of proteins inactivated by oxidation. These attenuators of transcription, mainly expressed during exponential phase are over-expressed by the GISA strain at the stationary phase and are also abundant at the protein level. This observation suggests that the GISA strain is constitutively in a stress state, despite absence of antibiotic exposure.

Some proteins involved in purine biosynthesis were found slightly down-regulated in 14-4 compared to sensitive strains at the protein level, whereas none of the genes involved in this operon was found differentially expressed by microarray. This observation is in contradiction with a recent report showing a massive up regulation of the *pur *operon [26] in GISA strains isolated *in vitro*. Our study, performed on *ex vivo *derived clinical isolates involves probably different mechanisms yielding to the resistant phenotype. These differences illustrate also the importance of growth conditions and antibiotic resistance selection. Indeed, these different conditions allow pointing out complementary mechanisms leading to reduced susceptibility to glycopeptides. In our comparison, metabolic targets showing differential expression between GISA and susceptible strains concerned mainly the metabolism of arginine. Arginine repressor is up-regulated and consequently argininosuccinate lyase expression is reduced as observed previously in response to cell-wall active antibiotics [53]. The potential effect is the decreased succinate and fumarate levels and the accumulation of aspartate. Aspartate is a central compound involved in numerous metabolic functions such as amino acids synthesis, urea cycle or energetic transports and cell-wall synthesis. Another category of targets found differentially regulated in our study is ABC transporters. Some of these compounds have been proposed as targets for immunotherapy [38]. In our study, 4 ABC transporters were found down-regulated in GISA compared to susceptible strain, suggesting that the GISA phenotype does not involve this type of target.

### Correlation of proteomic and transcriptomic profiles

In order to compare the transcriptomic profiles with the proteomic expression ratios, the differentially expressed proteins were compared to trend of gene-expression ratio (Tables 1 and 2). A significant part of the over-expressed proteins corresponds to down-regulated transcripts and suggests altered protein degradation. Comparison of GISA with either of the sensitive strains showed similarities between up- or down-regulated functions between the transcriptome and the proteome for all functional classes. However, these tendencies are inverted for the COG functions O and P, corresponding to post-translational modification, protein turnover, chaperones; and inorganic ion transport and metabolism, respectively. Indeed, most proteins in these functional groups are up-regulated whereas most transcripts are down-regulated. Similarities are observed by comparing the glycopeptide intermediate strain with the two susceptible strains. The analysis revealed also that most of the expressions have the same trend in proteomic and in transcriptomic (mainly related to a reduced metabolism in the GISA) but some regions showed different directions. These regions are probably composed of genes showing altered turn-over rates [50,54], as previously observed [46]. Finally, genes and proteins found differentially expressed showing the same trend represent potential targets for rapid diagnostic of evolution to GISA phenotype. These lists contain SA0536.1 (*vraX*), SA0591 and SA2113, two hypothetical proteins and *msrR*. Additionally, SA0532 showing an elevated difference in expression between 14-4 and MRGR3 appeared also as a potential marker of interest. The expression at the mRNA level was evaluated in a collection of unrelated strains showing altered susceptibility to glycopeptides. Among our collection, 5 unrelated genetic backgrounds were identified and all of them displayed intermediate level of resistance to glycopeptides. The level of expression of 6 markers found up-regulated in our *in vivo *isolated strain was very similar to that observed in the ancestral GISA strains. One of these markers, SA0536.1, has been very recently identified as up-regulated in GISA strains [30]. In accordance with this report, SA0536.1 was found up-regulated in all the genetically unrelated strains of our study. However this study observed that the up-regulation of SA0536.1 is not related to vancomycin induction [30] but is constitutive in all our strains. McAleese suggested as "provocative findings" that the expression of several markers is constitutive as revealed by our work.

Compared to the different studies published in the field and elaborating on potential mechanisms involved in glycopeptide resistance, this study was performed using GISA strains recovered in the absence of antibiotic pressure. However, most genes previously discovered in different GISA strains, either selected *in vitro *or *in vivo*, were also documented in our study. To the involvement of cell-wall biosynthesis as contributor of the GISA phenotype, our analysis now adds the discovery of new targets present in higher abundance in the GISA strains. Our determination revealed also the contribution of the *msr *locus, as well as that of several transporters that could contribute to a more direct resistance mechanism. In that sense, the presence at the protein levels of these targets appeared as a strong evidence. Thus, our results suggest that glycopeptide resistance is a stable phenomenon acquired by *Staphylococcus aureus *and expressed in the absence of any selection pressure. Finally, coupled to a rapid genotyping method, we identified markers potentially involved in the resistance mechanisms, potentially amenable to be used as diagnostic markers.

## Conclusion

Combined proteomic and transcriptomic analyses allowed obtaining a global view of complex processes involving differentially regulated factors contributing to antibiotic resistance. This combined information is essential for the global integration of the data. Several potential genes and proteins leading to glycopeptide resistance were identified. This study provides the identity of targets potentially related to the bacterial evolution toward a GISA phenotype, thus a contribution to the elucidation of complex mechanisms leading to glycopeptide resistance. In view of the results found in the literature and additional information obtained in this study, we showed that our genetic background appeared particularly relevant and that multiple mechanisms are mobilized by *Staphylococcus aureus *to evolve to the GISA phenotype. In addition, several markers reflecting the potential evolution of *Staphylococcus aureus *to the GISA phenotype have been identified.

## Methods

### Reagents and chemicals

All chemicals purchased were of the highest purity grade, unless otherwise stated. MilliQ water (Millipore, Bedford, MA) was used for the preparation of all buffers and solvents. Methanol, hydrochloric acid, magnesium chloride, potassium chloride, saccharose were purchased from Merck (Darmstadt, Germany). Acetonitrile (AcN) was purchased from Biosolve (Valkenswaard, The Netherland). High boiling-point petroleum ether, SDS, orthophosphoric acid and 2,2,2-trifluoroethanol (99.0%) were purchased from Fluka (Buchs, Switzerland). Trifluoroacetic acid (TFA), α-cyano-4-hydroxycinnamic acid, 1,4-dithioerythritol (DTE), ammonium bicarbonate, potassium chloride, potassium dihydrogenophosphate, iodoacetamide, glycerol, glycine, phosphate buffered saline, porcine trypsin and Tris were from Sigma-Aldrich (St. Louis, MO). IPG strips (3–10, nonlinear, 18 cm) were purchased from GE Healthcare (Piscataway, NJ). Agarose, ampholines (3–10) and molecular mass markers were purchased from BioRad (Hercules, CA). Mueller Hinton broth was obtained from Difco (Detroit, MI), and saccharose from Merck (Darmstadt, Germany). The murolytic enzyme lysostaphin (Ambicin) was purchased from Applied Microbiology Inc (Tarrytown, NY). dCTP coupled to cyanine dyes were obtained from NEN (Perkin Elmer, Boston, MA, USA).

### Strains, growth conditions and time point

MRSA strain MRGR3, a glycopeptide-susceptible strain was isolated from a patient with catheter-related sepsis [31]; it is highly pathogenic in a rat model of chronic infections [55]. Strain 14-4, a stable isogenic GISA strain was recovered from an experimental infection with MRGR3 [31]. Strain 14-4Rev that spontaneously emerged during infection with strain 14-4 is a revertant glycopeptide-susceptible isolate [32]. For protein extracts, strains MRGR3, 14-4 and 14-4Rev were grown with agitation at 37°C in Mueller Hinton Broth (MHB; 200 mL in 1000-mL flask), as previously described [56]. At stationary phase (OD_540 nm _= 6 corresponding to 2–3 × 10^9 ^cells/ml), cells were chilled on ice and harvested by centrifugation at 8'000 × *g *for 5 min at 4°C. For preparation of crude membrane extracts, 20 mL culture aliquots were washed in 1.1 M saccharose-containing buffer [46], then suspended in 2 mL aliquots of the same buffer containing 50 μg/mL of the hydrolytic enzyme lysostaphin for 10 min at 37°C. Protoplasts were recovered after centrifugation (30 min at 8,000 × *g*) and hypo-osmotic shock was applied in the presence of 10 μg/mL DNase I (Fluka, Buchs, Switzerland) to decrease the viscosity of the medium. Crude membrane pellets were obtained after ultracentrifugation at 50,000 × *g *for 50 min in a Beckman Optima TLX (Beckman Coulter Intl SA, Nyon, Switzerland).

Evaluation of membrane extracts purity-Evaluation of membrane enrichment was performed by assaying the lactate dehydrogenase following a previously described method [57,58] in a LX20 Beckman-Coulter (Beckman Coulter Intl SA, Nyon, Switzerland). In addition, total and membrane protein extracts (20 μg) were separated on SDS-PAGE, transferred to PVDF membrane [59] then incubated with anti-protein A (clone spa-27, Sigma, at a 1:750 dilution). Anti-mouse IgG coupled to phosphatase (1:7500 dilution) was used to reveal specific binding. A large 50 kD smeary band was obtained in total protein extracts whereas in membrane-enriched fraction, the staining was close to the limit of detection of the Western blot procedure (see Additional file 9).

A strain collection showing altered susceptibility levels to glycopeptides (Table 5) was obtained from the Network on Antimicrobial Resistance in *Staphylococcus aureus *(NARSA, Virginia, USA) [60].

### Proteomics experiments

Two-dimensional gel electrophoresis was performed using previously described conditions [47]. Quantitative-MS based proteomic experiments were performed in duplicates, on two individual cultures. For each experiment, an estimated amount of 300 μg of crude membrane protein extract (BCA method, Pierce, Rockford, IL) from each strain was separately dissolved, reduced, alkylated, digested and labeled with iTRAQ compounds according to the manufacturer's procedure (Applied Biosystems, Framingham MD). For the first proteomic experiment (PR1), strain MRGR3 was labeled with iTRAQ 114, strain 14-4 with iTRAQ 116 and 14-4Rev with iTRAQ 117. For the second proteomic replicate experiment (PR2), strain MRGR3 was labeled with iTRAQ 117, strain 14-4 with iTRAQ 114 and 14-4Rev with iTRAQ 115. After mixing, peptides from all strains were concentrated and desalted using an Oasis HLB 1 cc 10 mg solid-phase extraction cartridge (Waters, Milford, MA). Desalted peptides were re-suspended in 300 μl isoelectric focusing buffer containing 4 M urea and 0.5% ampholines in 50% TFE. The IPG strips were rehydrated overnight with the peptide solution. Isoelectric focusing was performed with the following conditions: Linear gradient from 0 to 3'500 V in 3 hours, and 3'500 V during 20 hours. After isoelectric focusing, the IPG strip was washed 3 times 10 seconds in 3 distinct baths containing high boiling point petroleum ether in order to remove the paraffin oil. The strip was then manually cut in 58 (PR1) and 64 (PR2) fractions with a scalpel, and gel pieces were placed in polypropylene tubes containing 70 μl 0.1% TFA. After 30 minutes, TFA solution was transferred into another tube and replaced with 0.1% TFA in 50% Acetonitrile (AcN). After 30 minutes, the solution was also transferred to the second tube and replaced with 0.1% TFA in AcN, also transferred after 30 minutes. The combined solution was then dry-evaporated, re-suspended in 25 μl HPLC buffer A (0.1% formic acid in 5% AcN) and stored at -20°C. A volume of 5 μl of peptide solution of each fraction was loaded on a 10 cm long home-made column with an ID of 100 μm, packed with C_18 _reverse phase (YMS-ODS-AQ200, Michrom BioResource, Auburn, CA), and eluted directly on a MALDI target using a home-made spotting robot. The elution gradient ranged from 4% to 38% solvent B (0.1% formic acid in 80% AcN) in 40 minutes. Peptides were analyzed in MS and MS/MS mode using a 4700 MALDI-TOF/TOF tandem mass spectrometer (Applied Biosystems, Framingham, MA).

After MS/MS analysis, peak lists from each fraction were created with embedded software (4700 explorer 2.0 peak-to-mascot) and merged together before database searching using the Phenyx platform from GeneBio (Geneva, Switzerland). Searching was performed against a home-made database containing all predicted ORFs from genome-sequenced strain N315 [42] and proteins from other *S. aureus *and *S. epidermidis *strains with less than 90% identity (5515 entries extracted from UniProt (release 46, 25 Jan. 2005) and TrEMBL (release 29, 25 Jan. 2005)). Multiple-peptide hits were accepted with individual peptide z-score higher than 6, single-peptide hits with z-scores higher than 8, corresponding to a predicted peptide false-positive ratio of respectively less than 6% and 3.5%. These values were obtained after plotting false positive rates against true positive rates on a ROC-like curve. False positive hits where obtained from searches in the entire SwissProt TrEMBL database without species restriction and true positive were selected from validated, high-score peptides matching to *S. aureus *entries.

Areas of the iTRAQ reporter ions for each peptide were extracted with Phenyx software. Peptides with highest iTRAQ reporter fragment ion areas below 2000 (arbitrary unit from "peak to mascot" software) were excluded. Mean peptide expression ratio was mathematically centered on value 1. Before prediction of transmembrane (TM) segments, the signal peptide of the predicted protein sequences was removed using the SignalP V2.0 tool [61], accessible through the internet [62]. The number of TM segments was then predicted using the TMHMM2.0 tool [63], also accessible through the internet [64]. Functional classes of all predicted ORFs were retrieved from the COG database [65,66], publicly available from the internet [67].

### Microarray manufacturing

The microarray was manufactured by *in situ *synthesis of 8'454 long oligonucleotide probes (Agilent, Palo Alto, CA, USA), selected as previously described [33]. It covers >99% of all ORFs annotated in strains N315, Mu50 [42], MW2 [68] and COL [69]. Briefly, the microarray contains 8'192 *S. aureus *specific oligonucleotides. Based on available sequence information 89% of the probes are common to the 4 strains used for the design, whereas 11% are strain-specific capture elements. Extensive experimental validation of this array has been described previously, using CGH, mapping of deletion and quantitative RT-PCR [33].

### Preparation of labeled nucleic acids

*S. aureus *strains were grown overnight in MHB, as described for proteomics analysis. Total RNA was extracted from 2 mL of cells at 2–3 × 10^9 ^cells/ml, using the RNeasy kit (Qiagen, Basel, Switzerland), as previously described [32,33]. After additional DNase treatment, the absence of remaining DNA traces was evaluated by quantitative PCR (SDS 7700; Applied Biosystems, Framingham, MA) with assays specific for 16s rRNA and the *HU *genes [32,70], encoding for a DNA-binding protein. Batches of 10 μg total *S. aureus *RNA were labeled by Cy-3 dCTP using the SuperScript II (Invitrogen, Basel, Switzerland) following manufacturer's instructions. Labeled was then purified onto QiaQuick columns (Qiagen). Purified genomic DNA from the 4 sequenced strains was extracted (DNeasy, Qiagen), labeled with Cy-5 dCTP using the Klenow fragment of DNA polymerase I (BioPrime, Invitrogen, Carlsbad, CA) [33,71].

### Hybridization and scanning parameters

Cy5-labeled DNA (0.125 μg per stain) and Cy3-labeled cDNA (10 μg) mixture was diluted in 250 μl Agilent hybridization buffer, and hybridized at a temperature of 60°C for 17 hours in a dedicated hybridization oven (Robbins Scientific, Sunnyvale, CA, USA). Slides were washed, dried under nitrogen flow, and scanned (Agilent, Palo Alto, CA, USA) using 100% PMT power for both wavelengths. Data were extracted and processed using Feature Extraction™ software (version 6.1.1, Agilent). For expression analysis, local background-subtracted signals were corrected for unequal dye incorporation or unequal load of labeled product. The algorithm consisted of a rank consistency filter and a curve fit using the default LOWESS (locally weighted linear regression) method. Data consisting of three independent biological experiments were expressed as Log_10 _ratios and analyzed using GeneSpring 7.0 (SiliconGenetics, Redwood City, CA, USA). Statistical significance of differentially expressed genes was identified by variance analysis (ANOVA) [72], performed using GeneSpring, including the Benjamini and Hochberg false discovery rate correction (5%).

### Real-time PCR validation of discovered markers

Gene-specific probes were designed using Primer Express 2.0 (Applied Biosystems). Oligonucleotide primers and probes (Table 6) obtained from Eurogentec (Seraing, Belgium) or Applied Biosystems (minor groove binder coupled to dark quencher) were solubilized in water and reactions were assembled in a one-step RT-PCR enzymatic mixture (Invitrogen) in a final volume of 15 μl. Reaction was performed in a SDS 7500 (Applied Biosystems). All measurements were performed in triplicate from two independent samples of purified RNA (0.5 ng/reaction) obtained from overnight and log-phase cultures isolated as previously described [33,73]. Cycle thresholds were assessed using default parameters. Briefly, the standard deviation of fluorescent values recorded from cycles 3–15 was multiplied by 10 to define the cycle threshold line. Cycle thresholds (Ct) were then derived from the intercept between this line and the signal obtained during the RT-PCR reaction. Results were normalized using intensity levels recorded for the rRNA 16s gene as previously described [32]. Figure shows relative gene expression for all strains as compared to 14-4Rev.

Genotyping of the related strains – Multi-locus Sequence Typing (MLST) was performed using previously described procedure and primers [74]. Allele numbers were assigned according to MLST Web site [75]. Comparative genome hybridization was performed according to the previously described procedure [76].

### Genotyping of *Staphylococcus aureus *collection by a variable number of tandem repeat (VNTR) approach

A previously published method was used to evaluate the genomic content of strains composing the analyzed collection [76,77]. Briefly, this assay is based on a multiplex PCR using ten primer pairs targeting genes showing variable number of tandem repeats. This method shows at least similar discriminatory power as that of pulse field-gel electrophoresis [77]. In addition to the NARSA collection, fully sequenced control strains were subjected to the assay.

## Authors' contributions

AS performed all proteomic experiments including data analysis and interpretation. MB performed transcriptomic experiments. PF contributed to the design, analysis and interpretation of transcriptomic and expression experiments. YC, TK and AH were involved in transcriptomic and proteomic data comparison. AF and FG performed genotyping and Taqman experiments including data analysis. JMD participated to the design and optimization of the isoelectric focusing procedure. PV and DL were involved in revising the article critically. ARV contributed to estimate membrane enrichment procedure, AR contributed to the isogenic characterization of strains. JSZ helped with the use of iTRAQ compounds. AM and PAB optimized the protein identification and quantification software for this study as well as the corresponding statistical analysis. CGZI and JCS participated in the design and coordination of the study. DH and JS designed the study and coordinated it. All authors read and approved the final manuscript.

## Supplementary Material

Additional file 1 Comparative genome hybridization using microarray. CGH performed with labeled gDNA from strains MRGR3 and 14-4. showing that strains MRGR3 and 14-4 generated strictly identical patterns. Click here for file

Additional file 2 Raw quantitative proteomic data obtained for the comparison between MRGR3 vs 14-4Rev. Table showing quantitative proteomics measurements of protein expression between strains MRGR3 (parental strain) and 14-4Rev (susceptible revertant of GISA 14-4) Click here for file

Additional file 3 Raw quantitative proteomic data obtained for the comparison between MRGR3 vs 14-4. Table showing quantitative proteomics measurements of protein expression between strains MRGR3 (parental strain) and 14-4 (GISA) Click here for file

Additional file 4 Raw quantitative proteomic data obtained for the comparison between 14-4 vs 14-4Rev. Table showing quantitative proteomics measurements of protein expression between strains 14-4 (GISA) and 14-4Rev (susceptible revertant of GISA 14-4) Click here for file

Additional file 5 Relative quantification of all identified proteins and trend of mRNA expression obtained by microarray for the comparison between strains MRGR3 and 14-4. Table showing the comparison between protein expression and trend of mRNA expression measured by microarray for strain MRGR3 (parental) strain and 14-4 (GISA) Click here for file

Additional file 6 Relative quantification of all identified proteins and trend of mRNA expression obtained by microarray for the comparison between strains 14-4 and 14-4Rev. Table showing the comparison between protein expression and trend of mRNA expression measured by microarray for strain 14-4 (GISA) strain and 14-4Rev (susceptible revertant of GISA 14-4) Click here for file

Additional file 7 Differentially expressed transcripts between 14-4 and MRGR3. Table showing all differentially expressed genes measured by using microarray between strains 14-4 (GISA) and MRGR3 (parental strain) Click here for file

Additional file 8 Differentially expressed transcripts between 14-4 and 14-4Rev. Table showing all differentially expressed genes measured by using microarray between strains 14-4 (GISA) and 14-4Rev (susceptible revertant of GISA 14-4) Click here for file

Additional file 9 Evaluation of membrane purity by Western blot. Coomassie blue stained SDS-PAGE and Western blot performed with anti-protein A antibody allowing to evaluate enrichment in membrane proteins of the protein fractions. Click here for file

## Figures and Tables

**Table 1:** Transcriptomic expression direction for the differentially expressed proteins between strains 14-4 and MRGR3.

		**Proteomic**		
	
		**Up**	**=**	**Down**
	
	**Up**	57		12
**Transcriptomic**	**=**	-	684	
	**Down**	36		26

**Table 2:** Transcriptomic expression direction for the differentially expressed proteins between strains 14-4 and 14-4Rev.

		**Proteomic**		
	
		**Up**	**=**	**Down**
	
	**Up**	30		12
**Transcriptomic**	**=**		718	
	**Down**	15		31

**Table 3:** List of significantly differentially expressed genes corresponding to over- or under-expressed proteins between 14-4 and MRGR3.

ORF number	Function	Transcript	Protein
SA0536.1	vraX	up	up
SA0591	hypothetical protein	up	up
SA1195	peptide methionine sulfoxide reductase regulator MsrR	up	up
SA2113	hypothetical protein	up	up

**Table 4:** List of significantly differentially expressed genes corresponding to over- or under-expressed proteins between 14-4 and 14-4Rev.

ORF number	Function	Transcript	Protein
SA0022	hypothetical protein, similar to 5'-nucleotidase	down	down
SA0591	hypothetical protein	up	up
SA0977	cell surface protein	down	down
SA1195	peptide methionine sulfoxide reductase regulator MsrR	up	up
SA1691	similar to penicillin-binding protein 1A/1B	up	up
SA2113	hypothetical protein	up	up

**SA0591**. Shows the **VLTHEFGHVL **motif, a characteristic of neutral Zn metallo-protease. Membrane associated protein.

**SA2113**. Shows partial homology with DNA repair protein from *Psychrobacter arcticus *and is particularly conserved among the different *Staphylococcus aureus *strains (often strictly identical nucleotide sequence).

**SA0536.1**. 1–19 N-terminal amino-acids are signal peptide (probably exported).

**Table 5:** Characteristics of strains subjected to real-time PCR quantification

Strain Name	CMI		
	Vanco	Teico	Characteristics and origin
NRS 3	4	8	Peritoneal fluid, USA
NRS 11	2	2	Ophthalmologic infection, France
NRS 12	8	8	Ophthalmologic infection, France
NRS 13	4	4	Ophthalmologic infection, France
NRS 14	8	8	Ophthalmologic infection, France
NRS 17	8	8	Bloodstream, USA
NRS 35	2	2	Bloodstream, France
NRS 56	8	8	Wound/skin/soft tissue, Brazil
NRS 63	8	8	Bloodstream, Oman
NRS 64	2	2	Bloodstream, Oman
Mu50 (NRS1)	8	8	Wound/skin/soft tissue, Japan [42]
Mu3 (NRS2)	0.5	2	Purulent sputum, Japan
			
MRGR3	1	1	Bloodstream, Switzerland
14-4	8	8	Sub-cutaneous infection with MRGR3, Switzerland [31]
14-4Rev	1	1	Sub-cutaneous infection with 14-4, Switzerland [32]
MW2	1	1	Community-acquired *Staphylococcus aureus *[68]
COL	0.5	1	Ancestor strain of MRSA, England [80]
N315	0.5	1	Pharyngeal smear, Japan [42]

**Table 6:** List and characteristics of primers and probes used for validation of identified markers.

Gene number^**A**^	Sequence (5'→3')	Length	5'-Dye	Concentration (nM)
***SA0591***				
F_SA0591-195F	TGGCCGTGCAAAAGATTTAGT	21		200
R_SA0591-275R	TGTGTAATTGCGAATCCTTGTTG	23		200
P_SA0591-219T	TGTAACAAGTCCACGAGAGCGACAACAGA	29	FAM	100
***SA2113***				
F_SA2113-40F	GCCGGTTTAAATGACCGTACTACT	24		200
R_SA2113-117R	TTGCAACGCATCACAAAATTG	21		200
P_SA2113-75T	CACTTTTGCATAACTTACTT^B^	20	VIC	100
***SA1195***				
F_SA1195-799F	CCTGATTCAGGGATTTTCCAAA	22		200
R_SA1195-877R	TTGGCACAGTCAATGACTTAACATC	25		200
P_SA1195-822T	TCACCTCGGATGCCAAAACTCAAACCT	27	FAM	100
***SA1691***				
F_SA1691-319F	CATCATGGATTCGATTTGAAAGG	23		200
R_SA1691-389R	CCTTGCACATCTCTGTCGCTAA	22		200
P_SA1691-344T	CAACTAGAGCTTTATTTTCA^B^	20	FAM	100
***SA0536.1***				
F_SA0536.1-31F	GGCGCACCAGTTTATGAAATTAT	23		200
R_SA0536.1-111R	TTCAGTATCACTAAATGAATCGTCACAT	28		200
P_SA0536.1-60T	AACGTTTCAGCATGTTT^B^	17	VIC	100
***SA0532***				
F_SA0532-15F	GATTGCGATAACTTCTTTTCTCTAT	25		200
R_SA0532-115R	AATCAAACTTAAGCCCTAGATAAA	24		200
P_SA0532-51T	CAGTATCAAATTTATCTAGGGCTTA^B^	25	VIC	100
***rRNA16S***				
F_rRNA16s-551F	GGCAAGCGTTATCCGGAATT	20		200
R_rRNA16S-651R	GTTTCCAATGACCCTCCACG	20		200
P_rRNA16s-573T	CCTACGCGCGCTTTACGCCCA	21	FAM	100

^**A **^Based on the annotation of N315 genome.

^**B**^ Minor groove binder (MGB) probes with non-fluorescent quencher bound to the 3'-end (Applied Biosystems).
