# Supplementary material for: Exploring glycopeptide-resistance in Staphylococcus aureus: a combined proteomics and transcriptomics approach for the identification of resistance-related markers
Source: BMC Genomics. 2006 Nov 22;7:296. doi: 10.1186/1471-2164-7-296 (PMC1687195; doi:10.1186/1471-2164-7-296)
Supplement: Additional file 1 — Comparative genome hybridization using microarray. CGH performed with labeled gDNA from strains MRGR3 and 14-4. showing that strains MRGR3 and 14-4 generated strictly identical patterns. [file 1471-2164-7-296-S1.pdf]

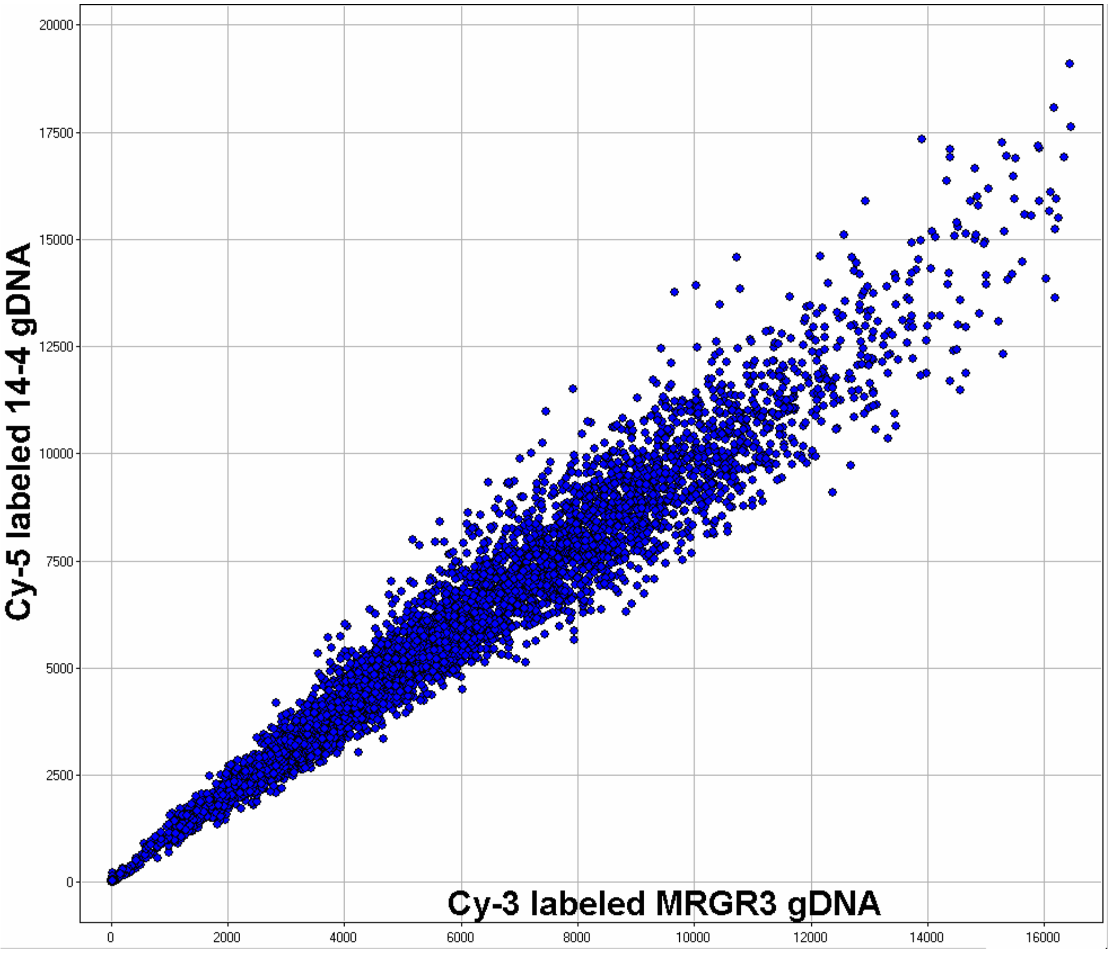

Additional file 1: Comparative genome hybridization using microarray. Comparative genome hybridization performed with labeled gDNA from strains MRGR3 and 14-4. Based on this determination on 5'427 oligonucleotide probes (covering 4 *S. aureus* genomes), whose design and validation were recently described (33), strains MRGR3 and 14-4 generated strictly identical patterns.
