# Supplementary material for: Exploring glycopeptide-resistance in Staphylococcus aureus: a combined proteomics and transcriptomics approach for the identification of resistance-related markers
Source: BMC Genomics. 2006 Nov 22;7:296. doi: 10.1186/1471-2164-7-296 (PMC1687195; doi:10.1186/1471-2164-7-296)
Supplement: Additional file 3 — Raw quantitative proteomic data obtained for the comparison between MRGR3 vs 14-4. Table showing quantitative proteomics measurements of protein expression between strains MRGR3 (parental strain) and 14-4 (GISA) [file 1471-2164-7-296-S3.pdf]

Additional file 3 : Raw quantitative proteomic data obtained for the comparison between MRGR3 vs 14-4

				Proteomic experiment 1 (PE1)				Proteomic experiment 2 (PE2)			
ORF number	AC	Description	TM domains	MRGR3/14-4	CV [%]	log(MRGR3/14-4)	nb. peptides	MRGR3/14-4	CV [%]	log(MRGR3/14-4)	nb. peptides
SA0001	P68866	Chromosomal replication initiator protein dnaA	0	1.82	24	0.26	3	1.43	18	0.15	2
SA0005	P66937	DNA gyrase subunit B (EC 5.99.1.3)	0	1.04	32	0.02	11	1.45	36	0.16	6
SA0006	Q99XG5	DNA gyrase subunit A (EC 5.99.1.3)	0	0.92	30	-0.04	12	1.95	31	0.29	8
SA0011	Q7A8E5	SA0011 protein	0					0.57	58	-0.25	2
SA0013	Q7A8E3	Hypothetical protein SA0013	2	0.45	16	-0.34	5	0.37	24	-0.43	7
SA0014	P66318	50S ribosomal protein L9	0	1.04	56	0.02	10	0.36	65	-0.44	5
SA0017	Q7A8E1	Response regulator	0	0.94	51	-0.03	5	1.43	23	0.16	7
SA0018	Q7A8E0	Two-component sensor histidine kinase	2	1.81	133	0.26	7	0.61	11	-0.21	1
SA0019	Q7A8D9	Hypothetical protein SA0019	1					0.88	3	-0.06	2
SA0022	Q99XE9	SA0022 protein	0	0.88		-0.05	1	1.56		0.19	1
SA0023	Q9WVW7	Hypothetical UPF0247 protein SA0023	0	0.87	24	-0.06	4	1.00	16	0.00	4
SA0038	Q7A8C6	Penicillin binding protein 2 prime	1	0.82	33	-0.09	29	0.48	26	-0.31	18
SA0041	Q99XE2	XylR protein	0	2.13	3	0.33	2	1.17	22	0.07	2
SA0045	Q7A8C2	Hypothetical protein SA0045	0					1.24	39	0.09	2
SA0089	Q7A890	SA0089 protein	0	1.96	26	0.29	3	0.85	33	-0.07	5
SA0097	Q7A882	SA0097 protein	0	0.58		-0.24	1				
SA0100	Q7A879	Hypothetical protein SA0100	9	0.99	48	0.00	5	0.35	29	-0.46	5
SA0108	Q7A872	SarH1 protein	0	2.62	70	0.42	3	2.08	98	0.32	3
SA0111	Q7A869	Lipoprotein	0					1.04	49	0.02	2
SA0134	P99100	Phosphopentomutase (EC 5.4.2.7) (Phosphodeoxyribomutase)	0					1.23	39	0.09	6
SA0143	Q7A843	Alcohol-acetaldehyde dehydrogenase	0	3.84	54	0.58	14	1.55	93	0.19	10
SA0144	Q7A842	Capsular polysaccharide synthesis enzyme Cap5A	2	0.36	38	-0.45	2	0.10	41	-0.98	2
SA0145	Q99X66	Capsular polysaccharide synthesis enzyme Cap5B	0	0.47	4	-0.33	2				
SA0147	Q7A840	Capsular polysaccharide synthesis enzyme Cap5D	4	0.30	50	-0.52	5	0.15	41	-0.83	4
SA0155	Q7A832	Capsular polysaccharide synthesis enzyme Cap5L	0	0.41		-0.39	1	0.14	40	-0.84	2
SA0156	Q7A831	Capsular polysaccharide synthesis enzyme Cap5M	1	0.31	10	-0.51	4	0.15	5	-0.81	2
SA0157	Q7A830	Capsular polysaccharide synthesis enzyme Cap5N	0					0.13	61	-0.89	3
SA0159	Q7A828	Capsular polysaccharide synthesis enzyme Cap5P	0	0.54	48	-0.27	3	0.47	59	-0.33	2
SA0162	Q7A825	AldA protein	0	0.27	9	-0.57	2				
SA0173	Q7A813	SA0173 protein	0	0.88	36	-0.06	18	1.15	35	0.06	12
SA0182	Q7A808	SA0182 protein	0	0.79	26	-0.10	3	0.79	12	-0.10	1
SA0183	Q7A807	GlcA protein	10	0.86	12	-0.06	3	0.89	18	-0.05	4

				Proteomic experiment 1 (PE1)				Proteomic experiment 2 (PE2)			
ORF number	AC	Description	TM domains	MRGR3/14-4	CV [%]	log(MRGR3/14-4)	nb. peptides	MRGR3/14-4	CV [%]	log(MRGR3/14-4)	nb. peptides
SA0184	Q7A806	Hypothetical protein SA0184	0	1.50	32	0.18	3	1.77	41	0.25	6
SA0186	Q7A804	SA0186 protein	10	1.09	19	0.04	7	0.73	50	-0.13	4
SA0187	Q7A803	SA0187 protein	0	1.94	26	0.29	4	1.01	36	0.00	3
SA0189	Q7A801	Probable type I restriction enzyme restriction chain	0	2.79	48	0.45	5	2.97	74	0.47	2
SA0206	Q7A7Y6	Multiple sugar-binding transport ATP-binding protein	0	1.25	24	0.10	5	0.70	28	-0.15	7
SA0207	Q7A7Y5	SA0207 protein	0	0.91	21	-0.04	5	0.83	19	-0.08	2
SA0208	Q7A7Y4	SA0208 protein	8					3.20		0.51	1
SA0215	Q7A7X9	SA0215 protein	0					0.62	47	-0.21	2
SA0218	Q7A7X6	Formate acetyltransferase	0	1.75	40	0.24	56	1.69	43	0.23	64
SA0219	Q7A7X5	Formate acetyltransferase activating enzyme	0	1.30	1	0.11	2	2.93	40	0.47	3
SA0229	Q7A7W6	SA0229 protein	0	0.40	40	-0.40	6	0.30	29	-0.52	5
SA0231	Q7A7W3	SA0231 protein	0	0.67	11	-0.17	2	1.01	32	0.01	6
SA0243	Q7A7V2	SA0243 protein	0	0.92	46	-0.04	7	0.44	40	-0.35	6
SA0244	Q7A7V1	SA0244 protein	0	0.44	38	-0.35	9	0.25	18	-0.60	5
SA0245	Q7A7V0	2-C-methyl-D-erythritol 4-phosphate cytidylyltransferase 2 (EC 2.7.7.60) (4-diphosphocytidyl-2C-methyl-D-erythritol synthase 2) (MEP cytidylyltransferase 2) (MCT 2)	0	0.30		-0.52	1				
SA0246	Q7A7U9	SA0246 protein	0	2.83	22	0.45	2	3.42	70	0.53	3
SA0247	Q7A7U8	SA0247 protein	0	0.65	35	-0.18	4	0.43	33	-0.37	2
SA0248	Q7A7U7	SA0248 protein	0	0.52	61	-0.28	8	0.41	24	-0.39	7
SA0295	Q7A7Q2	SA0295 protein	0	0.95	33	-0.02	8	0.57	38	-0.25	5
SA0296	Q7A7Q1	Hypothetical protein SA0296	4	1.08	18	0.03	3	1.14	31	0.06	1
SA0317	Q7A7N5	SA0317 protein	0	0.86	31	-0.07	3	0.89	24	-0.05	4
SA0339	Q7A7L5	SA0339 protein	0	0.79	45	-0.10	6	0.60	13	-0.22	2
SA0342	Q7A7L2	SA0342 protein	0	1.55	28	0.19	3	3.90	61	0.59	5
SA0348	Q7A7K7	SA0348 protein	0	0.70	30	-0.16	2	0.75	12	-0.13	3
SA0351	Q7A7K4	SA0351 protein	0	1.24	52	0.09	3	1.74	39	0.24	4
SA0352	P99142	30S ribosomal protein S6	0	1.43	34	0.16	12	1.06	35	0.03	8
SA0354	P66468	30S ribosomal protein S18	0	1.61	45	0.21	3	0.58	90	-0.24	5
SA0359	Q7A7J8	Hypothetical protein SA0359	0	0.72	24	-0.14	8	0.45	14	-0.34	2
SA0361	Q7A7J4	SA0361 protein	0					1.12		0.05	1
SA0363	Q7A7J2	Hypothetical protein SA0363	0	0.76	12	-0.12	3	0.67	7	-0.17	1
SA0365	P99118	Alkyl hydroperoxide reductase subunit F (EC 1.6.4.-)	0					2.06	63	0.31	4

				Proteomic experiment 1 (PE1)				Proteomic experiment 2 (PE2)			
ORF number	AC	Description	TM domains	MRGR3/14-4	CV [%]	log(MRGR3/14-4)	nb. peptides	MRGR3/14-4	CV [%]	log(MRGR3/14-4)	nb. peptides
SA0366	Q53647	Alkyl hydroperoxide reductase subunit C	0	0.25	36	-0.60	6				
SA0368	Q7A7I9	SA0368 protein	9	0.33	36	-0.48	2	0.44	19	-0.36	3
SA0371	Q99WJ3	Hypothetical protein SA0371	0					1.00	35	0.00	3
SA0373	Q7A7I5	Xanthine phosphoribosyltransferase	0	1.98	51	0.30	3	1.32	43	0.12	2
SA0375	P99106	Inosine-5'-monophosphate dehydrogenase (EC 1.1.1.205) (IMP dehydrogenase) (IMPDH) (IMPD)	0	1.07		0.03	1	2.15	24	0.33	2
SA0376	P99105	GMP synthase [glutamine-hydrolyzing] (EC 6.3.5.2) (Glutamine amidotransferase) (GMP synthetase)	0					2.86	36	0.46	5
SA0397	Q7A7G5	Hypothetical protein lpl2	0	1.01	50	0.00	2				
SA0406	Q7A7F7	Hypothetical protein SA0406	0					1.13	30	0.05	2
SA0416	Q7A7E6	SA0416 protein	0	0.30		-0.53	1				
SA0422	Q7A7E1	SA0422 protein	0	0.42		-0.37	1	0.41	53	-0.39	2
SA0423	Q7A7E0	SA0423 protein	0	1.14	18	0.06	2	0.45	11	-0.34	2
SA0424	Q7A7D9	Hypothetical protein SA0424	2					0.29	22	-0.54	1
SA0428	Q7A7D5	Hypothetical protein SA0428	8					0.29		-0.54	1
SA0432	Q7A7D1	PTS enzyme II, phosphoenolpyruvate-dependent, trehalose-specific	9	1.28	98	0.11	2	0.56	39	-0.25	4
SA0433	Q7A7D0	Alpha-glucosidase	0					1.04	9	0.02	3
SA0435	Q7A7C8	Hypothetical protein SA0435	0					0.91		-0.04	1
SA0436	Q7A7C7	DNA polymerase III gamma and tau subunits	0	0.49	15	-0.31	5	0.55	15	-0.26	4
SA0447	Q7A7C1	Hypothetical protein SA0447	0	1.39	27	0.14	2	0.73		-0.14	1
SA0448	P67579	Methionyl-tRNA synthetase (EC 6.1.1.10) (Methionine--tRNA ligase) (MetRS)	0	2.82	46	0.45	4	3.07	56	0.49	11
SA0454	Q7A7B7	PurR protein	0	1.23	34	0.09	3	0.96	43	-0.02	6
SA0456	Q7A7B5	SpoVG protein	0	1.56	77	0.19	4	1.87	34	0.27	4
SA0457	Q7A7B4	GcaD protein	0	1.37	34	0.14	3	1.49	26	0.17	4
SA0458	P65237	Ribose-phosphate pyrophosphokinase (EC 2.7.6.1) (RPPK) (Phosphoribosyl pyrophosphate synthetase) (P-Rib-PP synthetase) (PRPP synthetase)	0	1.33	14	0.12	5	1.14	37	0.06	6
SA0459	Q7A7B3	50S ribosomal protein L25	0	1.15	44	0.06	6	0.78	27	-0.11	7
SA0461	Q7A7B2	Transcription-repair coupling factor	0	0.92	4	-0.04	2	0.97	82	-0.01	3
SA0463	Q7A7B0	Hypothetical protein SA0463	0					1.17		0.07	1
SA0464	Q7A7A9	Hypothetical protein SA0464	0	0.86		-0.07	1				

				Proteomic experiment 1 (PE1)				Proteomic experiment 2 (PE2)			
ORF number	AC	Description	TM domains	MRGR3/14-4	CV [%]	log(MRGR3/14-4)	nb. peptides	MRGR3/14-4	CV [%]	log(MRGR3/14-4)	nb. peptides
SA0465	Q7A7A8	SA0465 protein	1					0.86	98	-0.07	3
SA0466	Q7A7A7	SA0466 protein	0	1.70	23	0.23	1	0.91	5	-0.04	3
SA0469	Q7A7A5	Cell-division protein	2	0.52	123	-0.29	15	0.33	44	-0.48	15
SA0471	P63871	Cysteine synthase (EC 2.5.1.47) (O-acetylserine sulfhydrylase) (O-acetylserine (Thiol)-lyase) (CSase)	0	0.37		-0.43	1	1.27	34	0.10	4
SA0475	P67610	Lysyl-tRNA synthetase (EC 6.1.1.6) (Lysine--tRNA ligase) (LysRS)	0	0.70	25	-0.15	4	0.38	173	-0.43	6
SA0477	P60798	Pyridoxine biosynthesis protein pdx1	0					1.30	60	0.11	2
SA0478	Q7A7A1	Hypothetical protein SA0478	0					0.91	34	-0.04	2
SA0479	Q7A7A0	Pyrimidine nucleoside transport protein	10	0.86	29	-0.06	1	0.69	9	-0.16	1
SA0480	Q7A799	CtsR protein	0	0.44	12	-0.36	3	0.42	16	-0.37	3
SA0482	P65206	Hypothetical ATP:guanido phosphotransferase SA0482 (EC 2.7.3.-)	0	0.79	4	-0.10	2	0.78	18	-0.11	5
SA0483	Q7A797	Endopeptidase	0	1.34	34	0.13	9	1.31	50	0.12	16
SA0484	Q7A796	RadA protein	0	1.08	20	0.03	2	0.65		-0.19	1
SA0485	Q7A795	Hypothetical protein SA0485	4	0.56	21	-0.25	5	0.41		-0.39	1
SA0487	P67765	Serine acetyltransferase (EC 2.3.1.30) (SAT)	0	0.93	12	-0.03	4	0.67	20	-0.17	2
SA0490	Q7A794	SA0490 protein	0	0.93	19	-0.03	3				
SA0494	O08386	Transcription antitermination protein nusG	0	0.62	2	-0.21	2	1.27	9	0.10	4
SA0495	O06443	50S ribosomal protein L11	0	1.03	20	0.01	3	0.67	11	-0.17	4
SA0496	Q99W68	50S ribosomal protein L1	0	0.92	46	-0.04	7	0.57	14	-0.24	4
SA0497	P99155	50S ribosomal protein L10	0	1.60	21	0.20	5	0.84	28	-0.08	7
SA0498	P99154	50S ribosomal protein L7/L12	0	2.55	67	0.41	3	1.07	14	0.03	6
SA0499	Q7A790	Hypothetical protein SA0499	0	1.05	34	0.02	3	1.05	7	0.02	2
SA0500	P60278	DNA-directed RNA polymerase beta chain (EC 2.7.7.6) (RNAP beta subunit) (Transcriptase beta chain) (RNA polymerase beta subunit)	0	0.82	34	-0.09	33	2.32	69	0.36	13
SA0501	P60285	DNA-directed RNA polymerase beta' chain (EC 2.7.7.6) (RNAP beta' subunit) (Transcriptase beta' chain) (RNA polymerase beta' subunit)	0	0.69	53	-0.16	43	1.12	22	0.05	20
SA0503	P48942	30S ribosomal protein S12	0	1.33	32	0.12	5	0.90	17	-0.04	2
SA0504	P66616	30S ribosomal protein S7	0	1.47	44	0.17	11	0.92	21	-0.04	9
SA0505	P68789	Elongation factor G (EF-G) (85 kDa vitronectin binding protein)	0	1.18	47	0.07	12	1.90	43	0.28	18

				Proteomic experiment 1 (PE1)				Proteomic experiment 2 (PE2)			
ORF number	AC	Description	TM domains	MRGR3/14-4	CV [%]	log(MRGR3/14-4)	nb. peptides	MRGR3/14-4	CV [%]	log(MRGR3/14-4)	nb. peptides
SA0506	P99152	Elongation factor Tu (EF-Tu)	0	1.03	40	0.01	36	1.08	29	0.03	32
SA0508	P60120	2-amino-3-ketobutyrate coenzyme A ligase (EC 2.3.1.29) (AKB ligase) (Glycine C-acetyltransferase)	0	0.80		-0.10	1	1.66	30	0.22	4
SA0511	Q7A788	SA0511 protein	0	3.30	47	0.52	2	1.74	61	0.24	5
SA0512	P99138	Probable branched-chain-amino-acid aminotransferase (EC 2.6.1.42) (BCAT)	0					1.40	39	0.15	3
SA0513	Q7A787	Hypothetical protein SA0513	0	0.27	17	-0.57	2				
SA0517	Q7A783	Hypothetical protein SA0517	0					2.21		0.34	1
SA0518	Q7A782	Hypothetical protein SA0518	0					1.40	31	0.15	2
SA0522	Q7A779	SA0522 protein	0	1.37	48	0.14	4	1.10	2	0.04	2
SA0523	Q7A778	SA0523 protein	0	1.22	43	0.09	4	1.23	50	0.09	7
SA0531	Q7A771	ProP protein	12	1.12		0.05	1				
SA0536.1	Q99W32	Hypothetical protein SAS016	0	0.08		-1.12	1	0.11		-0.94	1
SA0537	Q7A765	SA0537 protein	0	2.34	1	0.37	1	2.97	41	0.47	3
SA0544	Q7A759	Hypothetical protein SA0544	0					0.97	28	-0.01	2
SA0547	Q7A757	Mevalonate kinase	0					1.25	47	0.10	3
SA0549	Q7A755	Phosphomevalonate kinase	0	0.88	7	-0.05	2				
SA0557	Q7A747	SA0557 protein	0					1.82	44	0.26	3
SA0561	Q7A743	Hypothetical protein SA0561	0	1.11	65	0.04	4	0.99	27	-0.01	3
SA0562	Q7A742	Alcohol dehydrogenase I	0	1.33	44	0.12	3	4.46	110	0.65	13
SA0566	Q7A739	SA0566 protein	0	0.80	20	-0.10	5				
SA0569	Q7A736	SA0569 protein	0	0.72	19	-0.14	6	0.52	46	-0.28	4
SA0570	Q7A735	Hypothetical protein SA0570	0	0.42	14	-0.38	2	0.16	32	-0.80	3
SA0572	Q7A733	SA0572 protein	0	1.26	48	0.10	2				
SA0573	Q7A732	Staphylococcal accessory regulator A	0	2.48	86	0.39	6	0.82	111	-0.09	6
SA0587	Q7A719	SA0587 protein	0	0.90	36	-0.04	31	0.67	33	-0.17	22
SA0589	Q7A717	SA0589 protein	0	0.68	25	-0.17	9	0.57	31	-0.25	5
SA0591	Q7A715	Hypothetical protein SA0591	6	0.12	73	-0.91	2	0.05	79	-1.29	3
SA0592	Q7A714	Teichoic acid biosynthesis protein	0					0.48	20	-0.31	3
SA0593	Q7A713	Teichoic acids export ATP-binding protein tagH (EC 3.6.3.40)	0	0.39		-0.41	1	0.50	21	-0.30	3
SA0595	Q7A712	Teichoic acid biosynthesis protein B	0	0.45		-0.35	1	0.40	5	-0.40	2
SA0596	Q7A711	Teichoic acid biosynthesis protein X	0					0.41	48	-0.39	2
SA0598	Q7A709	Penicillin binding protein 4	0	0.57	22	-0.24	3	0.70	67	-0.15	4

ORF number	AC	Description	TM domains	Proteomic experiment 1 (PE1)				Proteomic experiment 2 (PE2)			
				MRGR3/14-4	CV [%]	log(MRGR3/14-4)	nb. peptides	MRGR3/14-4	CV [%]	log(MRGR3/14-4)	nb. peptides
SA0599	Q7A708	ATP-binding cassette transporter A	6	0.52	36	-0.29	12	0.48	36	-0.31	9
SA0600	Q7A706	SA0600 protein	10	0.76		-0.12	1				
SA0601	Q7A705	Hypothetical protein SA0601	4	0.60	12	-0.22	2	0.45	25	-0.35	4
SA0606	Q7A700	Hypothetical protein SA0606	0	0.88	13	-0.05	2	1.72	48	0.24	3
SA0615	Q7A6Z3	SA0615 protein	2	0.70	28	-0.15	2				
SA0616	Q7A6Z2	ABC transporter ATP-binding protein	0					0.64	30	-0.19	2
SA0618	Q7A6Z0	Hypothetical protein SA0618	0	0.57	36	-0.24	5	0.32	16	-0.49	4
SA0624	P67182	Hypothetical UPF0082 protein SA0624	0	0.69	9	-0.16	2	1.16	57	0.06	2
SA0626	Q7A6Y4	Hypothetical protein SA0626	0					0.65	14	-0.19	2
SA0630	Q7A6Y0	Hypothetical protein SA0630	4	0.55		-0.26	1				
SA0633	Q7A6X7	Hypothetical protein SA0633	0	0.81	33	-0.09	7	0.32	38	-0.50	4
SA0637	Q7A6X4	Hypothetical protein SA0637	0	0.89	54	-0.05	8				
SA0639	Q99VT7	SA0639 protein	5					0.29	10	-0.53	1
SA0640	Q7A6X3	SA0640 protein	5	0.51	20	-0.29	4				
SA0641	Q7A6X2	SA0641 protein	0	1.64	46	0.21	2	0.78	5	-0.11	3
SA0643	Q7A6X0	SA0643 protein	0	2.21	31	0.35	3	0.89	30	-0.05	3
SA0655	Q7A6V9	Fructose specific permease	9	0.64	25	-0.19	5	0.66	19	-0.18	8
SA0658	Q7A6V6	SA0658 protein	0	0.92	44	-0.03	2	0.66	139	-0.18	2
SA0659	Q7A6V5	SA0659 protein	2					0.60		-0.22	1
SA0660	Q7A6V4	Histidine protein kinase	2	0.80	30	-0.10	7	0.63	18	-0.20	7
SA0661	Q7A6V3	Response regulator	0					0.65	10	-0.19	2
SA0663	Q7A6V1	Hypothetical protein SA0663	0	0.74	34	-0.13	13	0.46	35	-0.34	8
SA0673	Q7A6U2	Hypothetical protein SA0673	0	1.24	9	0.09	2				
SA0675	Q7A6U0	SA0675 protein	0	0.54	29	-0.27	4	0.90	55	-0.05	6
SA0677	Q7A6T8	SA0677 protein	0					0.54	2	-0.27	1
SA0678	Q7A6T7	SA0678 protein	6	0.47	30	-0.33	3	0.48	20	-0.32	3
SA0682	Q7A6T5	SA0682 protein	13					0.42	9	-0.38	3
SA0686	Q7A6T2	Ribonuceloside diphosphate reductase major subunit	0	0.96	26	-0.02	15	0.92	14	-0.04	11
SA0691	Q7A6S7	SA0691 protein	0	0.91	7	-0.04	2	0.51	29	-0.29	4
SA0693	P65463	UDP-N-acetylenolpyruvoylglucosamine reductase (EC 1.1.1.158) (UDP-N-acetylmuramate dehydrogenase)	0	1.27		0.11	1				
SA0695	Q7A6S4	Hypothetical protein SA0695	1					0.47	61	-0.33	3
SA0707	Q7A6R6	Hypothetical protein SA0707	0	1.34	28	0.13	5	0.85	39	-0.07	7

				Proteomic experiment 1 (PE1)				Proteomic experiment 2 (PE2)			
ORF number	AC	Description	TM domains	MRGR3/14-4	CV [%]	log(MRGR3/14-4)	nb. peptides	MRGR3/14-4	CV [%]	log(MRGR3/14-4)	nb. peptides
SA0708	Q7A6R5	Preprotein translocase secA subunit	0	1.13	34	0.05	22	1.11	26	0.05	12
SA0709	Q7A6R4	Peptide chain release factor 2 (RF-2)	0					0.38	37	-0.42	2
SA0711	Q7A6R2	Hypothetical protein SA0711	0					3.36		0.53	1
SA0713	P67425	UvrABC system protein B (UvrB protein) (Excinuclease ABC subunit B)	0	1.95	73	0.29	2	2.03	50	0.31	3
SA0714	P63383	UvrABC system protein A (UvrA protein) (Excinuclease ABC subunit A)	0	1.75	26	0.24	8	1.34	67	0.13	6
SA0715	P60701	HPr kinase/phosphorylase (EC 2.7.1.-) (EC 2.7.4.-) (HPrK/P) (HPr(Ser) kinase/phosphorylase)	0	1.19	26	0.08	10	0.78	15	-0.11	6
SA0718	Q7A6Q8	Hypothetical protein SA0718	0	1.33		0.12	1				
SA0720	P67109	Hypothetical UPF0042 protein SA0720	0	0.81	21	-0.09	6				
SA0721	Q7A6Q7	Hypothetical protein SA0721	0	1.64		0.21	1	2.03	18	0.31	2
SA0724	Q7A6Q5	SA0724 protein	0	0.60	43	-0.22	13	0.32	30	-0.50	10
SA0727	Q9Z5C5	Glyceraldehyde-3-phosphate dehydrogenase 1	0	0.59	61	-0.23	3	0.36	47	-0.44	6
SA0728	P99135	Phosphoglycerate kinase (EC 2.7.2.3)	0					1.61	62	0.21	16
SA0730	P64270	2,3-bisphosphoglycerate-independent phosphoglycerate mutase (EC 5.4.2.1) (Phosphoglyceromutase) (BPG-independent PGAM) (iPGM)	0					2.26	34	0.35	5
SA0731	P99088	Enolase (EC 4.2.1.11) (2-phosphoglycerate dehydratase) (2-phospho-D-glycerate hydro-lyase)	0	0.39	31	-0.41	15	0.25	36	-0.60	15
SA0733	Q7A6Q1	Probable protein-export membrane protein	2	0.45	44	-0.35	1				
SA0735	Q99VK1	Ribonuclease R	0	0.91	16	-0.04	11	0.47	35	-0.33	10
SA0741	Q7A6P6	Hypothetical protein SA0741	0	0.38	22	-0.42	2	0.37	7	-0.43	1
SA0744	Q7A6P4	Extracellular ECM and plasma binding protein	0	0.34	36	-0.47	2	0.41	0	-0.38	1
SA0759	Q7A6M6	SA0759 protein	0	1.79	0	0.25	1				
SA0769	Q7A6M2	SA0769 protein	0					0.50	13	-0.30	1
SA0771	Q7A6M0	Hypothetical protein SA0771	0	0.54	56	-0.27	5	0.43	46	-0.37	5
SA0774	Q7A6L7	SA0774 protein	0	1.44	16	0.16	9	1.63	33	0.21	11
SA0775	Q7A6L6	Hypothetical protein SA0775	0					0.48	12	-0.32	2
SA0776	P99177	Probable cysteine desulfurase	0					1.37		0.14	1
SA0777	Q7A6L5	SA0777 protein	0					0.36	44	-0.45	2
SA0780	Q7A6L2	SA0780 protein	4	0.59	11	-0.23	3	0.37	11	-0.44	1

				Proteomic experiment 1 (PE1)				Proteomic experiment 2 (PE2)			
ORF number	AC	Description	TM domains	MRGR3/14-4	CV [%]	log(MRGR3/14-4)	nb. peptides	MRGR3/14-4	CV [%]	log(MRGR3/14-4)	nb. peptides
SA0785	P65286	Lipoyl synthase (EC 2.8.1.-) (Lipoic acid synthase) (Lipoate synthase) (Lipoyl-acyl-carrier protein synthase) (Sulfur insertion protein lipA) (Lip-syn)	0	0.98	26	-0.01	3				
SA0791	Q7A6K3	SA0791 protein	0					1.68	46	0.23	6
SA0796	Q7A6K0	Poly D-alanine transfer protein	1	0.68	47	-0.17	9	0.74	21	-0.13	4
SA0799	Q7A6J7	SA0799 protein	0	1.15	24	0.06	8	1.22	22	0.09	10
SA0802	Q7A6J4	SA0802 protein	0	0.29	25	-0.54	18	0.29	32	-0.54	13
SA0803	Q7A6J3	Probable cytosol aminopeptidase	0	0.52	23	-0.29	6				
SA0809	P60689	Na(+)/H(+) antiporter subunit E (Mnh complex subunit E)	4	0.46	28	-0.33	1				
SA0811	P60681	Na(+)/H(+) antiporter subunit C (Mnh complex subunit C)	3					0.32		-0.49	1
SA0813	P60675	Na(+)/H(+) antiporter subunit A (Mnh complex subunit A)	21	0.45		-0.35	1	0.25	7	-0.61	2
SA0814	Q7A6I2	Hypothetical protein SA0814	0	0.63	31	-0.20	2				
SA0815	Q7A6I1	Peptidyl-prolyl cis-trans isomerase (EC 5.2.1.8)	0	0.63	7	-0.20	2	0.28	28	-0.55	2
SA0818	P60298	Acetylornithine aminotransferase 2 (EC 2.6.1.11) (ACOAT 2)	0	0.92	13	-0.04	2	2.62	63	0.42	11
SA0819	Q7A6H8	NAD-specific glutamate dehydrogenase	0	0.40	22	-0.40	5				
SA0820	Q7A6H7	Glycerophosphoryl diester phosphodiesterase	0					1.06	45	0.02	3
SA0821	P63583	Argininosuccinate lyase (EC 4.3.2.1) (Arginosuccinase) (ASAL)	0					2.18		0.34	1
SA0823	P99078	Glucose-6-phosphate isomerase (EC 5.3.1.9) (GPI) (Phosphoglucose isomerase) (PGI) (Phosphohexose isomerase) (PHI)	0	0.17	5	-0.77	2				
SA0826	P72365	Signal peptidase IB	1	0.25	38	-0.60	12	0.13	39	-0.89	7
SA0828	Q7A6H4	SA0828 protein	0					0.78		-0.11	1
SA0829	Q7A6H3	SA0829 protein	0					2.45		0.39	1
SA0831	Q7A6H1	Coenzyme A disulfide reductase (EC 1.8.1.14) (CoA-disulfide reductase) (CoADR)	0	2.99		0.48	1	2.88	58	0.46	3
SA0832	Q7A6H0	Hypothetical protein SA0832	0	0.80	16	-0.10	4	1.56	29	0.19	4
SA0835	Q7A6G6	Chaperone clpB	0	1.46	46	0.16	4	1.30	67	0.11	5
SA0842	P99159	3-oxoacyl-[acyl-carrier-protein] synthase III (EC 2.3.1.41) (Beta-ketoacyl-ACP synthase III) (KAS III) (SaFabH)	0					1.07	54	0.03	2
SA0843	Q7A6F8	3-oxoacyl-synthase	0	2.93	46	0.47	3	3.29	58	0.52	3
SA0848	Q7A6F3	OppF protein	0	0.59	46	-0.23	3				
SA0849	Q7A6F2	SA0849 protein	0	0.70	21	-0.15	4				

				Proteomic experiment 1 (PE1)				Proteomic experiment 2 (PE2)			
ORF number	AC	Description	TM domains	MRGR3/14-4	CV [%]	log(MRGR3/14-4)	nb. peptides	MRGR3/14-4	CV [%]	log(MRGR3/14-4)	nb. peptides
SA0855	P67593	Tryptophanyl-tRNA synthetase (EC 6.1.1.2) (Tryptophan--tRNA ligase) (TrpRS)	0					1.15	13	0.06	6
SA0856	P60379	Regulatory protein spx	0	1.04	9	0.02	2				
SA0859	Q7A6E5	SA0859 protein	0					0.67		-0.18	1
SA0860	Q99V89	Hypothetical protein SA0860	0					0.84	42	-0.08	2
SA0864	Q99V85	GTP pyrophosphokinase	0	0.64	33	-0.19	4	1.18	31	0.07	2
SA0868	Q7A6D9	SA0868 protein	12	0.36	6	-0.45	2	0.36	17	-0.44	2
SA0869	Q7A6D8	Trans-2-enoyl-ACP reductase	0	0.73	24	-0.14	4	0.53	32	-0.27	9
SA0870	Q7A6D7	Hypothetical protein SA0870	9					0.81		-0.09	1
SA0873	Q7A6D4	Hypothetical protein SA0873	0	2.59	67	0.41	6	3.83	77	0.58	7
SA0876	P65480	UDP-N-acetylmuramoylalanyl-D-glutamate--2,6-diaminopimelate ligase (EC 6.3.2.13) (UDP-N-acetylmuramyl-tripeptide synthetase) (Meso-diaminopimelate-adding enzyme) (UDP-MurNAc-tripeptide synthetase)	0	0.36	45	-0.45	4	0.34	37	-0.47	3
SA0877	Q99V72	Peptide chain release factor 3 (RF-3)	0	0.72	51	-0.14	3	0.90	45	-0.05	2
SA0879	Q7A6C9	Serine protease HtrA	1	0.64	37	-0.19	8	0.57	19	-0.25	6
SA0898	Q7A6A9	Naphthoate synthase	0					2.15	70	0.33	6
SA0905	Q99V41	Autolysin	0	1.90	44	0.28	38	0.66	42	-0.18	45
SA0907	P52079	Hypothetical protein SA0907	0	0.11		-0.94	1				
SA0908	Q7A6A3	Hypothetical protein SA0908	0	0.38	13	-0.42	3				
SA0909	Q7A6A2	FmtA, autolysis and methicillin resistant-related protein	1	0.24	38	-0.62	11	0.17	48	-0.78	7
SA0912	Q7A699	Quinol oxidase polypeptide I QoxB	15	1.77	58	0.25	6	0.99	80	0.00	5
SA0913	Q7A698	SA0913 protein	2	1.74	42	0.24	18	0.88	62	-0.06	20
SA0915	Q7A697	FolD bifunctional protein	0					1.28	43	0.11	5
SA0917	Q7A695	PurK protein	0	2.15	49	0.33	6	2.44	57	0.39	4
SA0918	P99064	Phosphoribosylaminoimidazole-succinocarboxamide synthase (EC 6.3.2.6) (SAICAR synthetase)	0					1.98	66	0.30	4
SA0919	Q7A694	Hypothetical protein SA0919	0					0.51	23	-0.30	2
SA0922	P99164_WO	Amidophosphoribosyltransferase precursor (EC 2.4.2.14) (Glutamine phosphoribosylpyrophosphate amidotransferase) (ATASE) (GPATase)	0	1.47		0.17	1	1.69	10	0.23	2

				Proteomic experiment 1 (PE1)				Proteomic experiment 2 (PE2)			
ORF number	AC	Description	TM domains	MRGR3/14-4	CV [%]	log(MRGR3/14-4)	nb. peptides	MRGR3/14-4	CV [%]	log(MRGR3/14-4)	nb. peptides
SA0924	P99162	Phosphoribosylglycinamide formyltransferase (EC 2.1.2.2) (GART) (GAR transformylase) (5'-phosphoribosylglycinamide transformylase)	0					3.54	74	0.55	3
SA0925	P67544	Bifunctional purine biosynthesis protein purH [Includes: Phosphoribosylaminoimidazolecarboxamide formyltransferase (EC 2.1.2.3) (AICAR transformylase); IMP cyclohydrolase (EC 3.5.4.10) (Inosinicase) (IMP synthetase) (ATIC)]	0	2.72	68	0.44	15	1.49	90	0.17	9
SA0926	P65896	Phosphoribosylamine--glycine ligase (EC 6.3.4.13) (GARS) (Glycinamide ribonucleotide synthetase) (Phosphoribosylglycinamide synthetase)	0					2.85	70	0.46	3
SA0927	Q7A693	Hypothetical protein SA0927	6	1.15	22	0.06	4	0.95	25	-0.02	2
SA0928	Q7A692	SA0928 protein	0	1.22	33	0.09	8	0.86	24	-0.06	4
SA0931	Q7A689	Hypothetical protein SA0931	10	0.39	21	-0.41	2	0.27	46	-0.56	3
SA0932	Q7A688	Hypothetical protein SA0932	0	0.77	29	-0.12	7	0.57	26	-0.24	5
SA0933	Q7A687	Hypothetical protein SA0933	0	1.13	19	0.05	3	0.62	12	-0.21	5
SA0937	Q7A685	SA0937 protein	9	0.41	67	-0.39	2	0.40	16	-0.40	3
SA0939	Q7A683	Hypothetical protein SA0939	0	0.41	43	-0.39	3	0.32	56	-0.49	3
SA0940	Q7A682	Hypothetical protein SA0940	0	1.01	35	0.00	15	1.10	32	0.04	14
SA0943	Q7A681	Hypothetical protein SA0943	0	0.42	14	-0.38	6	0.43	19	-0.36	2
SA0943.1	Q820A6	Pyruvate dehydrogenase E1 component, alpha subunit (EC 1.2.4.1)	0	2.16	69	0.33	9	1.54	47	0.19	7
SA0944	Q9L6H5	Pyruvate dehydrogenase E1 component, beta subunit (EC 1.2.4.1)	0	2.18	56	0.34	10	1.68	63	0.23	13
SA0945	P65636	Dihydrolipoyllysine-residue acetyltransferase component of pyruvate dehydrogenase complex (EC 2.3.1.12) (E2) (Dihydrolipoamide acetyltransferase component of pyruvate dehydrogenase complex)	0	3.05	51	0.48	13	1.64	47	0.21	13
SA0946	Q59822	Dihydrolipoyl dehydrogenase (EC 1.8.1.4)	0	2.04	30	0.31	10	3.31	88	0.52	18
SA0950	Q7A679	Spermidine/putrescine import ATP-binding protein potA (EC 3.6.3.31)	0	0.63	8	-0.20	5	0.88	63	-0.06	5
SA0953	Q7A676	PotD protein	1	0.91	41	-0.04	3	0.58	38	-0.24	3
SA0954	Q7A675	Hypothetical protein SA0954	3	0.56	40	-0.25	5	0.32	17	-0.49	3
SA0957	Q99UZ6	Hypothetical protein SA0957	0	1.43	12	0.15	4	0.56	46	-0.25	3

				Proteomic experiment 1 (PE1)				Proteomic experiment 2 (PE2)			
ORF number	AC	Description	TM domains	MRGR3/14-4	CV [%]	log(MRGR3/14-4)	nb. peptides	MRGR3/14-4	CV [%]	log(MRGR3/14-4)	nb. peptides
SA0958	Q7A673	SA0958 protein	0					1.41	54	0.15	2
SA0959	Q7A671	SA0959 protein	0	1.24	16	0.09	5	1.01	21	0.01	5
SA0963	Q7A666	Pyruvate carboxylase	0	0.70	50	-0.15	4	0.78	55	-0.11	4
SA0969	Q7A661	SA0969 protein	1	0.39	49	-0.41	9	0.26	33	-0.58	7
SA0974	Q7A658	Hypothetical protein SA0974	0					0.69		-0.16	1
SA0975.1	P66210	50S ribosomal protein L32	0	1.15	47	0.06	1				
SA0977	Q7A655	Cell surface protein	1	2.75	96	0.44	5	1.95	56	0.29	2
SA0984	Q7A648	SA0984 protein	0					0.88	22	-0.06	2
SA0988	Q7A646	Hypothetical protein SA0988	0	0.70	28	-0.16	3				
SA0991	P65496	MutS2 protein	0	1.05	11	0.02	3	0.75	27	-0.13	4
SA0995	Q7A642	Succinate dehydrogenase flavoprotein subunit	0	0.72	35	-0.14	31	0.51	119	-0.30	25
SA0996	Q99UV7	Succinate dehydrogenase iron-sulfur protein subunit	0	0.91	77	-0.04	14	0.53	28	-0.28	8
SA0997	P63638	Glutamate racemase (EC 5.1.1.3)	0	0.75	29	-0.13	4	1.13	28	0.05	2
SA1000	Q7A639	SA1000 protein	0	0.36	60	-0.45	3	0.08	99	-1.08	3
SA1003	P68800	Fibrinogen-binding protein precursor	0	0.89	22	-0.05	3	0.58	39	-0.24	2
SA1008	Q7A631	Hypothetical protein SA1008	0	2.20	56	0.34	2				
SA1012	Q9K3A1	Ornithine carbamoyltransferase (EC 2.1.3.3) (OTCase)	0					2.42	42	0.38	3
SA1019	Q99UT4	Hypothetical protein SA1019	0	0.39	48	-0.41	4	0.47	107	-0.33	5
SA1021	P65439	Protein mraZ	0	1.37	35	0.14	2	1.00	14	0.00	2
SA1022	P60392	S-adenosyl-methyltransferase mraW (EC 2.1.1.-)	0	0.81	18	-0.09	4	0.74	34	-0.13	3
SA1024	Q7A619	Penicillin-binding protein 1	1	0.55	40	-0.26	8	0.41	27	-0.39	11
SA1025	P68783	Phospho-N-acetylmuramoyl-pentapeptide-transferase	10	0.62	18	-0.21	3				
SA1026	O33595	UDP-N-acetylmuramoylalanine--D-glutamate ligase (EC 6.3.2.9)	0					1.31	15	0.12	2
SA1027	Q7A618	Div1b protein	1	0.69	24	-0.16	5	0.47	26	-0.33	4
SA1028	P63765	Cell division protein ftsA	0				2	1.31	23	0.12	11
SA1029	P45498	Cell division protein ftsZ	0	1.64	44	0.22	12	2.44	63	0.39	16
SA1031	Q7A616	Hypothetical protein SA1031	0					1.61	43	0.21	2
SA1035	Q7A612	SA1035 protein	0					0.63		-0.20	1
SA1036	P67509	Isoleucyl-tRNA synthetase (EC 6.1.1.5) (Isoleucine--tRNA ligase) (IleRS)	0					1.46	37	0.16	3
SA1040	Q7A610	Pseudouridine synthase (EC 4.2.1.70) (Uracil hydrolyase)	0	0.40	34	-0.40	9	0.29	33	-0.54	8

				Proteomic experiment 1 (PE1)				Proteomic experiment 2 (PE2)			
ORF number	AC	Description	TM domains	MRGR3/14-4	CV [%]	log(MRGR3/14-4)	nb. peptides	MRGR3/14-4	CV [%]	log(MRGR3/14-4)	nb. peptides
SA1041	P65944	PyrR bifunctional protein [Includes: Pyrimidine operon regulatory protein; Uracil phosphoribosyltransferase (EC 2.4.2.9) (UPRTase)]	0	1.14	32	0.06	5	1.69	57	0.23	4
SA1043	P65618	Aspartate carbamoyltransferase (EC 2.1.3.2) (Aspartate transcarbamylase) (ATCase)	0	2.23	43	0.35	7	2.40	75	0.38	4
SA1044	P65906	Dihydroorotase (EC 3.5.2.3) (DHOase)	0					1.81	69	0.26	4
SA1045	P99147	Carbamoyl-phosphate synthase small chain (EC 6.3.5.5) (Carbamoyl-phosphate synthetase glutamine chain)	0	4.02	37	0.60	2	2.66	44	0.42	4
SA1046	P63740	Carbamoyl-phosphate synthase large chain (EC 6.3.5.5) (Carbamoyl-phosphate synthetase ammonia chain)	0	3.16	52	0.50	6	3.57	115	0.55	8
SA1051	Q7A606	SA1051 protein	0	0.93	5	-0.03	3	0.55	39	-0.26	3
SA1052	P99176	Guanylate kinase (EC 2.7.4.8) (GMP kinase)	0					2.51	29	0.40	2
SA1053	P66726	DNA-directed RNA polymerase omega chain (EC 2.7.7.6) (RNAP omega subunit) (Transcriptase omega chain) (RNA polymerase omega subunit)	0	1.79		0.25	1	3.11	0	0.49	1
SA1054	Q7A605	SA1054 protein	0	0.85	9	-0.07	5	0.55	17	-0.26	3
SA1060	Q7A601	SA1060 protein	0	1.17	22	0.07	3	1.23	11	0.09	2
SA1061	Q7A600	Hypothetical protein SA1061	0					0.48		-0.32	1
SA1063	Q7A5Z8	Protein kinase	1	0.47	33	-0.33	9	0.42	32	-0.38	9
SA1064	P67682	Probable GTPase engC (EC 3.6.1.-)	0	1.10	31	0.04	2	0.97	9	-0.01	2
SA1066	Q7A5Z6	Hypothetical protein SA1066	0					2.57		0.41	1
SA1068	Q7A5Z5	Hypothetical protein SA1068	0	1.02	15	0.01	3	0.92	36	-0.04	4
SA1069	Q7A5Z4	Conserved hypotehtical protein	0	1.49	13	0.17	4	1.76	48	0.25	7
SA1070	P64325	ATP-dependent DNA helicase recG (EC 3.6.1.-)	0	0.73	7	-0.14	2	0.56	6	-0.25	3
SA1071	P67620	Transcription factor fapR (Fatty acid and phospholipid biosynthesis regulator)	0					1.04	15	0.02	1
SA1072	P65739	Fatty acid/phospholipid synthesis protein plsX	0	0.84	36	-0.07	3	1.06	13	0.03	3
SA1073	Q7A5Z3	Malonyl CoA-acyl carrier protein transacylase	0	3.48		0.54	1	1.73	115	0.24	2
SA1074	Q99QK7	3-oxoacyl-[acyl-carrier-protein] reductase (EC 1.1.1.100)	0					2.36	94	0.37	7
SA1076	P66668	Ribonuclease III (EC 3.1.26.3) (RNase III)	0	1.06	26	0.03	3				
SA1077	Q7A5Z2	Chromosome segregation SMC protein	0	0.96	57	-0.02	2	1.12	73	0.05	4
SA1080	Q7A5Z0	Ffh protein	0	0.82	12	-0.09	3	1.37	41	0.14	3
SA1081	P66440	30S ribosomal protein S16	0	1.33	47	0.12	2	0.89	49	-0.05	2

				Proteomic experiment 1 (PE1)				Proteomic experiment 2 (PE2)			
ORF number	AC	Description	TM domains	MRGR3/14-4	CV [%]	log(MRGR3/14-4)	nb. peptides	MRGR3/14-4	CV [%]	log(MRGR3/14-4)	nb. peptides
SA1082	P66656	Probable 16S rRNA processing protein rimM	0					0.50	19	-0.30	4
SA1084	P66083	50S ribosomal protein L19	0	0.98	18	-0.01	3	0.73	28	-0.14	5
SA1086	Q7A5Y9	Hypothetical protein SA1086	0	0.99	23	0.00	3	0.45	25	-0.35	4
SA1088	P99071	Succinyl-CoA synthetase beta chain (EC 6.2.1.5) (SCS-beta)	0	1.80	48	0.25	9	1.73	162	0.24	12
SA1089	P99070	Succinyl-CoA synthetase alpha chain (EC 6.2.1.5) (SCS-alpha)	0					2.01	158	0.30	5
SA1093	Q7A5Y5	SA1093 protein	0	1.16	13	0.06	3	0.86	7	-0.07	4
SA1094	P64235	Protein gid homolog	0					0.20	63	-0.71	2
SA1098	P63844	GTP-sensing transcriptional pleiotropic repressor codY	0	1.27	26	0.10	8	1.09	23	0.04	6
SA1099	P66544	30S ribosomal protein S2	0	1.39	27	0.14	12	0.74	27	-0.13	10
SA1100	P99171	Elongation factor Ts (EF-Ts)	0					0.58	50	-0.24	2
SA1101	P65936	Uridylate kinase (EC 2.7.4.-) (UK) (Uridine monophosphate kinase) (UMP kinase)	0	0.88	49	-0.06	3	0.79	80	-0.10	3
SA1105	P63333	Hypothetical zinc metalloprotease SA1105 (EC 3.4.24.-)	5	0.33	9	-0.48	4	0.42	67	-0.38	3
SA1106	Q7A5Y3	Proline-tRNA ligase	0	0.29	57	-0.54	6	1.41	35	0.15	7
SA1107	P63982	DNA polymerase III polC-type (EC 2.7.7.7) (PolIII)	0	0.60	18	-0.22	3	0.55	36	-0.26	4
SA1109	Q7A5Y2	Transcription termination-antitermination factor	0	0.53	16	-0.27	4	0.69	6	-0.16	3
SA1112	P65134	Translation initiation factor IF-2	0	1.08	28	0.03	16	0.87	71	-0.06	15
SA1113	P65967	Ribosome-binding factor A	0	1.48	31	0.17	2	0.72	10	-0.14	2
SA1114	P65855	tRNA pseudouridine synthase B (EC 4.2.1.70) (tRNA pseudouridine 55 synthase) (Psi55 synthase) (Pseudouridylate synthase) (Uracil hydrolyase)	0	1.07	49	0.03	4	0.65	42	-0.19	2
SA1115	Q7A5X9	Riboflavin kinase / FAD synthase ribC	0	1.80	23	0.26	4				
SA1116	Q7A5X8	30Sribosomal protein S15	0					0.64	60	-0.19	4
SA1117	Q7A5X7	Polyribonucleotide nucleotidyltransferase	0	0.49	48	-0.31	13	0.53	22	-0.27	14
SA1118	Q7A5X6	Conserved hypotehtical protein	0	1.35	19	0.13	10	0.96	55	-0.02	8
SA1119	P64165	DNA translocase ftsK	5					0.49	23	-0.31	2
SA1124	Q7A5X1	Hypothetical protein SA1124	1					0.91		-0.04	1
SA1126	P63756	CDP-diacylglycerol--glycerol-3-phosphate 3-phosphatidyltransferase (EC 2.7.8.5) (Phosphatidylglycerophosphate synthase) (PGP synthase)	4					0.37	35	-0.43	2
SA1127	Q7A5W9	Competence-damage inducible protein cinA	0	1.29	40	0.11	2	0.89	4	-0.05	2
SA1128	P68844	RecA protein (Recombinase A)	0	1.17	22	0.07	8	0.81	39	-0.09	6
SA1129	P67278	Hypothetical UPF0144 protein SA1129	1	0.45	30	-0.35	9	0.33	44	-0.48	8

				Proteomic experiment 1 (PE1)				Proteomic experiment 2 (PE2)			
ORF number	AC	Description	TM domains	MRGR3/14-4	CV [%]	log(MRGR3/14-4)	nb. peptides	MRGR3/14-4	CV [%]	log(MRGR3/14-4)	nb. peptides
SA1130	Q7A5W7	Hypothetical protein SA1130	0	1.15	78	0.06	4	1.47	12	0.17	5
SA1133	Q7A5W4	Hypothetical protein SA1133	0	2.15		0.33	1	2.85		0.45	1
SA1141	P99113	Glycerol kinase (EC 2.7.1.30) (ATP:glycerol 3-phosphotransferase) (Glycerokinase) (GK)	0	1.07	20	0.03	11	1.92	95	0.28	12
SA1142	Q7A5V7	Aerobic glycerol-3-phosphate dehydrogenase	0	0.54	24	-0.27	21	0.29	43	-0.53	20
SA1143	Q7A5V6	SA1143 protein	0	0.58	36	-0.23	3	0.39	40	-0.41	4
SA1146	P99097	Glutathione peroxidase homolog bsaA	0	0.85	30	-0.07	3	0.41	48	-0.39	2
SA1149	Q7A5V2	Glutamine synthetase repressor	0					1.32		0.12	1
SA1150	P99095	Glutamine synthetase (EC 6.3.1.2) (Glutamate--ammonia ligase) (GS)	0	0.37	24	-0.43	4	0.71	41	-0.15	2
SA1155	Q7A5U5	SA1155 protein	2	0.44	1	-0.36	2	0.49	30	-0.31	2
SA1161	Q7A5T9	Hypothetical protein SA1161	1	0.57	36	-0.24	15	0.60	33	-0.22	8
SA1168	Q7A5T3	Hypothetical protein SA1168	0					0.82	29	-0.09	3
SA1170	Q7A5T2	Catalase (EC 1.11.1.6)	0	0.68	17	-0.17	9	0.41	19	-0.39	9
SA1172	P60563	GMP reductase (EC 1.7.1.7) (Guanosine 5'-monophosphate oxidoreductase) (Guanosine monophosphate reductase)	0	0.93	49	-0.03	5	2.06	26	0.31	3
SA1178	P67291	Hypothetical UPF0154 protein SA1178	1	0.55	4	-0.26	4	0.59	24	-0.23	6
SA1181	Q7A5S6	SA1181 protein	0	0.63		-0.20	1	0.84	81	-0.08	2
SA1183	Q99UC9	Glycine betaine transporter	11	0.44	11	-0.36	3	0.46	22	-0.34	2
SA1184	P99148	Aconitate hydratase (EC 4.2.1.3) (Citrate hydro-lyase) (Aconitase)	0					0.72	49	-0.14	6
SA1185	Q7A5S5	Hypothetical protein SA1185	0	1.44	20	0.16	2	0.88		-0.06	1
SA1186	Q7A5S4	Hypothetical protein SA1186	0	0.29	12	-0.54	2	0.26		-0.58	1
SA1187	P67164	Hypothetical UPF0078 protein SA1187	5					0.44		-0.36	1
SA1188	P66939	Topoisomerase IV subunit B (EC 5.99.1.-)	0	1.15	24	0.06	5	0.83	27	-0.08	5
SA1189	Q93KF4	Topoisomerase IV subunit A (EC 5.99.1.-)	0	1.43	15	0.15	3	0.65	57	-0.19	13
SA1190	Q7A5S3	Amino acid carrier protein	9	0.40	17	-0.39	2	0.49	21	-0.31	6
SA1192	Q7A5S0	Hypothetical protein SA1192	8	0.41	8	-0.39	2	0.35	24	-0.46	2
SA1193	Q7A5R9	Oxacillin resistance-related FmtC protein	14	0.34	36	-0.46	3	0.46	43	-0.33	2
SA1195	Q99Q02	Peptide methionine sulfoxide reductase regulator MsrR	1	0.26	30	-0.58	8	0.24	36	-0.62	3
SA1206	Q7A5R3	Factor essential for expression of methicillin resistance	0	0.80	25	-0.10	11	0.42	20	-0.38	9
SA1207	P14305	Protein femB	0	0.75	26	-0.12	11	0.61	29	-0.21	11
SA1210	Q7A5R0	Hypothetical protein SA1210	0	0.40	30	-0.40	3				

ORF number	AC	Description	TM domains	Proteomic experiment 1 (PE1) MRGR3/14-4	CV [%]	log(MRGR3/14-4)	nb. peptides	Proteomic experiment 2 (PE2) MRGR3/14-4	CV [%]	log(MRGR3/14-4)	nb. peptides
SA1217	Q7A5Q4	SA1217 protein	0					0.37		-0.43	1
SA1223	Q7A5Q1	Hypothetical protein SA1223	0	0.80	28	-0.10	4	1.16	11	0.07	3
SA1224	Q7A5Q0	SA1224 protein	0	0.76	0	-0.12	2	1.57	25	0.20	2
SA1238	P60108	TelA-like protein SA1238	0	0.47	76	-0.33	4	0.37	36	-0.43	7
SA1243	Q7A5N5	SA1243 protein	0	0.89	44	-0.05	4	1.43	53	0.15	3
SA1244	Q7A5N4	Dihydrolipoamide succinyltransferase	0	4.86	49	0.69	4	4.20	63	0.62	4
SA1245	Q99U74	2-oxoglutarate dehydrogenase E1	0	1.06	37	0.03	8	3.35	81	0.52	7
SA1246	Q7A5N3	Hypothetical protein arlS	2					0.76		-0.12	1
SA1251	P65482	UDP-N-acetylglucosamine--N-acetylmuramyl-(pentapeptide) pyrophosphoryl-undecaprenol N-acetylglucosamine transferase (EC 2.4.1.227) (Undecaprenyl-PP-MurNAc-pentapeptide-UDPGlcNAc GlcNAc transferase)	0	0.78	24	-0.11	14	0.52	23	-0.29	13
SA1253	Q7A5M9	Probable carboxy-terminal processing proteinase ctpA	1	0.26	35	-0.59	11	0.23	37	-0.64	4
SA1255	P60857	PTS system, glucose-specific IIA component (EIIA-Glc) (Glucose-permease IIA component) (Phosphotransferase enzyme II, A component) (EC 2.7.1.69) (EIII-Glc)	0					0.42		-0.38	1
SA1256	P99065	Peptide methionine sulfoxide reductase msrB (EC 1.8.4.6)	0	0.25	99	-0.60	3	0.16	67	-0.79	5
SA1257	P65446	Peptide methionine sulfoxide reductase msrA 2 (EC 1.8.4.6) (Protein-methionine-S-oxide reductase 2) (Peptide Met(O) reductase 2)	0	0.12	43	-0.92	10	0.16	38	-0.80	11
SA1258	P67371	Hypothetical UPF0230 protein SA1258	0	1.08	33	0.04	3	1.97	26	0.29	6
SA1261	Q7A5M6	Hypothetical protein SA1261	0					0.72	2	-0.14	2
SA1267	Q99U54	EbhA protein	1					0.79	37	-0.10	4
SA1271	Q7A5L8	SA1271 protein	0	3.23	42	0.51	5	4.76	73	0.68	5
SA1272	P99151	Alanine dehydrogenase 1 (EC 1.4.1.1)	0					3.21	45	0.51	4
SA1274	Q7A5L6	Hypothetical protein SA1274	0					2.17	27	0.34	5
SA1275	Q7A5L5	Hypothetical protein SA1275	5					0.96	67	-0.02	2
SA1276	Q7A5L4	Hypothetical protein SA1276	1	1.22	17	0.09	3	0.77	17	-0.11	2
SA1279	Q7A5L1	Hypothetical protein SA1279	0	0.39	33	-0.41	3	0.22	65	-0.65	3
SA1282	P68817	Recombination protein U homolog (Penicillin-binding protein-related factor A homolog) (PBP related factor A homolog)	0					0.53	54	-0.28	2
SA1283	Q7A5K8	PBP2	1	0.45	153	-0.35	21	0.14	33	-0.85	21

				Proteomic experiment 1 (PE1)				Proteomic experiment 2 (PE2)			
ORF number	AC	Description	TM domains	MRGR3/14-4	CV [%]	log(MRGR3/14-4)	nb. peptides	MRGR3/14-4	CV [%]	log(MRGR3/14-4)	nb. peptides
SA1287	P67572	Asparaginyl-tRNA synthetase (EC 6.1.1.22) (Asparagine--tRNA ligase) (AsnRS)	0	0.85	17	-0.07	6	0.78	20	-0.11	9
SA1288	Q7A5K4	Probable ATP-dependent DNA helicase dinG	0	1.14	6	0.06	2	0.59	38	-0.23	3
SA1293	Q7A5J9	Hypothetical protein SA1293	4	0.32	54	-0.49	6	0.24	30	-0.61	5
SA1302	Q7A5J3	Heptaprenyl diphosphate syntase component II	0	1.19	24	0.08	3	0.55	61	-0.26	3
SA1303	P67062	Menaquinone biosynthesis methyltransferase ubiE (EC 2.1.1.-)	0	0.44	23	-0.35	4	0.36	51	-0.44	5
SA1304	Q7A5J2	SA1304 protein	0					1.77	4	0.25	2
SA1305	Q7A5J1	DNA-binding protein II	0	3.00	35	0.48	4	1.13	44	0.05	9
SA1307	P64060	GTP-binding protein engA	0	2.88	39	0.46	7	2.78	36	0.44	3
SA1308	Q7A5J0	30S ribosomal protein S1	0	0.77	19	-0.11	8	1.00	22	0.00	9
SA1310	Q7A5I8	Probable L-asparaginase	0					0.59	50	-0.23	2
SA1311	Q7A5I7	SA1311 protein	0					3.11	5	0.49	2
SA1312	Q7A5I6	Elastin binding protein	1	0.83	19	-0.08	7	0.86	21	-0.07	4
SA1318	Q7A5I1	Hypothetical protein SA1318	0	0.70	27	-0.15	3				
SA1322	Q7A5H7	Staphylococcal respiratory response protein SrrB	2	0.64	31	-0.20	9	0.54	16	-0.27	5
SA1323	Q7A5H6	Staphylococcal respiratory response protein SrrA	0	1.29	81	0.11	2	1.96	46	0.29	2
SA1324	Q7A5H5	Pseudouridine synthase (EC 4.2.1.70) (Uracil hydrolyase)	0	0.99	13	0.00	6	0.74	29	-0.13	4
SA1329	Q7A5H3	SA1329 protein	0					0.74	42	-0.13	3
SA1331	Q7A5H1	Hypothetical protein SA1331	0	0.98	11	-0.01	2	0.71	27	-0.15	4
SA1338	Q7A5G5	Alpha-D-1,4-glucosidase	0					1.92	24	0.28	3
SA1345	Q7A5G0	Hypothetical protein SA1345	0					0.57	31	-0.24	3
SA1346	Q7A5F9	Branched-chain alpha-keto acid dehydrogenase E2	0	1.53		0.18	1	0.98	36	-0.01	3
SA1348	Q7A5F7	Branched-chain alpha-keto acid dehydrogenase E1	0					2.87		0.46	1
SA1350	Q7A5F5	DNA repair protein	0	0.99	36	-0.01	2	0.94	28	-0.03	6
SA1351	P63580	Arginine repressor	0					1.03	26	0.01	3
SA1355	P65578	N utilization substance protein B homolog (NusB protein)	0	0.28		-0.55	1	0.83	8	-0.08	2
SA1356	Q7A5F3	Hypothetical protein SA1356	0	0.84	31	-0.08	4	0.54	4	-0.27	1
SA1359	P99066	Elongation factor P (EF-P)	0					1.79	3	0.25	2
SA1361	Q7A5F0	Hypothetical protein SA1361	0	0.46	22	-0.34	3	0.44	30	-0.36	3
SA1363	Q7A5E8	Hypothetical protein SA1363	0	0.52	10	-0.28	4	0.42	37	-0.37	6
SA1364	Q7A5E7	Hypothetical protein SA1364	1	0.71	53	-0.15	6	0.70	16	-0.15	2

				Proteomic experiment 1 (PE1)				Proteomic experiment 2 (PE2)			
ORF number	AC	Description	TM domains	MRGR3/14-4	CV [%]	log(MRGR3/14-4)	nb. peptides	MRGR3/14-4	CV [%]	log(MRGR3/14-4)	nb. peptides
SA1365	P99168	Probable glycine dehydrogenase [decarboxylating] subunit 2 (EC 1.4.4.2) (Glycine decarboxylase subunit 2) (Glycine cleavage system P-protein subunit 2)	0	0.24	43	-0.63	12	1.17	11	0.07	3
SA1366	P64218	Probable glycine dehydrogenase [decarboxylating] subunit 1 (EC 1.4.4.2) (Glycine decarboxylase subunit 1) (Glycine cleavage system P-protein subunit 1)	0	0.40	54	-0.39	3	1.38	47	0.14	1
SA1367	P64225	Aminomethyltransferase (EC 2.1.2.10) (Glycine cleavage system T protein)	0					2.20	84	0.34	3
SA1377	Q7A5D8	Glucokinase	0					2.66	18	0.42	3
SA1379	Q7A5D7	Hypothetical protein SA1379	7	0.73	26	-0.14	3	0.63	3	-0.20	2
SA1381	Q7A5D5	Penicillin-binding protein 3	1	1.09	46	0.04	7	0.47	30	-0.32	7
SA1385	Q7A5D3	SA1385 protein	0	0.61	18	-0.22	4				
SA1387	Q7A5D2	SA1387 protein	0	0.72	20	-0.14	6	0.54	33	-0.26	4
SA1390	Q99TT5	RNA polymerase sigma factor rpoD	0	0.39	53	-0.41	4	0.50	12	-0.30	2
SA1393	Q7A5D0	Hypothetical protein SA1393	0					0.56	113	-0.25	3
SA1394	P99129	Glycyl-tRNA synthetase (EC 6.1.1.14) (Glycine--tRNA ligase) (GlyRS)	0	1.52	27	0.18	6	1.68	55	0.23	8
SA1396	P64085	GTP-binding protein era homolog	0	1.13	42	0.05	6	0.75	29	-0.13	6
SA1400	Q7A5C7	PhoH protein	0	1.34	21	0.13	5	0.82	31	-0.09	5
SA1401	Q7A5C6	Hypothetical protein SA1401	0	0.54		-0.27	1	0.54		-0.27	1
SA1402	Q7A5C5	Hypothetical protein SA1402	1	0.61	29	-0.21	14	0.54	30	-0.26	13
SA1404	P66521	30S ribosomal protein S21	0	1.05	28	0.02	2				
SA1405	Q7A5C4	Hypothetical protein SA1405	0					0.33		-0.48	1
SA1408	P63971	Chaperone protein dnaJ (HSP40)	0	0.68	55	-0.17	3	0.78	71	-0.11	6
SA1409	P99110	Chaperone protein dnaK (Heat shock protein 70) (Heat shock 70 kDa protein) (HSP70)	0	0.51		-0.29	1	0.93	32	-0.03	8
SA1413	P65272	GTP-binding protein lepA	0	0.99	22	0.00	3	1.40	14	0.15	3
SA1414	Q7A5C0	30S ribosomal protein S20	0	1.41	28	0.15	3				
SA1417	Q7A5B8	Late competence operon required for DNA binding and uptake comEB	0	0.39	45	-0.41	2	0.50	14	-0.30	2
SA1423	Q7A5B3	Hypothetical protein SA1423	0	1.03	42	0.01	3	0.49	17	-0.31	2
SA1426	Q7A5B1	Hypothetical protein SA1426	0	0.73	39	-0.14	2				
SA1431	Q7A5A6	Hypothetical protein SA1431	0					0.86		-0.07	1

				Proteomic experiment 1 (PE1)				Proteomic experiment 2 (PE2)			
ORF number	AC	Description	TM domains	MRGR3/14-4	CV [%]	log(MRGR3/14-4)	nb. peptides	MRGR3/14-4	CV [%]	log(MRGR3/14-4)	nb. peptides
SA1434	Q7A5A5	SA1434 protein	0					1.06	20	0.03	4
SA1439	P67411	Uridine kinase (EC 2.7.1.48) (Uridine monophosphokinase) (Cytidine monophosphokinase)	0	0.65	9	-0.18	4	0.53	14	-0.27	4
SA1445	P60359	Hypothetical UPF0297 protein SA1445	0					0.57	15	-0.25	2
SA1447	Q7A597	SA1447 protein	0					0.85		-0.07	1
SA1449	Q99TM8	Probable tRNA (5-methylaminomethyl-2-thiouridylate)-methyltransferase (EC 2.1.1.61)	0	1.37	43	0.14	2	1.06	28	0.03	4
SA1450	Q7A595	SA1450 protein	0					0.75	16	-0.12	2
SA1453	Q7A592	Hypothetical protein SA1453	0	0.44		-0.36	1	0.50	16	-0.30	2
SA1454	Q7A591	Hypothetical protein SA1454	0	0.67	13	-0.17	2	0.24		-0.63	1
SA1455	Q7A590	Hypothetical protein SA1455	0	0.79		-0.10	1				
SA1456	P67015	Aspartyl-tRNA synthetase (EC 6.1.1.12) (Aspartate--tRNA ligase) (AspRS)	0					2.78	33	0.44	3
SA1458	Q7A588	N-acetylmuramoyl-L-alanine amidase	1					0.44		-0.35	1
SA1459	O32420	D-tyrosyl-tRNA(Tyr) deacylase (EC 3.1.-.-)	0	0.38		-0.42	1				
SA1460	Q99TL8	GTP pyrophosphokinase (EC 2.7.6.5) (ATP:GTP 3'-pyrophosphotransferase) (ppGpp synthetase I) ((P)ppGpp synthetase)	0	0.97	66	-0.01	7	1.27	37	0.10	4
SA1461	P68779	Adenine phosphoribosyltransferase (EC 2.4.2.7) (APRT)	0					2.76	37	0.44	2
SA1462	Q7A587	SA1462 protein	0					0.64		-0.19	1
SA1463	Q7A586	Protein-export membrane protein SecDF	12	0.70	34	-0.15	11	0.49	30	-0.31	6
SA1464	Q7A585	Hypothetical protein SA1464	1	0.50	91	-0.30	2	0.16	148	-0.80	2
SA1465	P66905	Queuine tRNA-ribosyltransferase (EC 2.4.2.29) (tRNA-guanine transglycosylase) (Guanine insertion enzyme)	0	0.97	89	-0.01	2	0.95	30	-0.02	2
SA1466	P65951	S-adenosylmethionine:tRNA ribosyltransferase-isomerase (EC 5.-.-.-) (Queuosine biosynthesis protein queA)	0	1.15	31	0.06	1	0.85	17	-0.07	2
SA1467	P66758	Holliday junction DNA helicase ruvB	0	0.76	21	-0.12	3	0.45	19	-0.34	2
SA1470	Q7A584	Spo0B-associated GTP-binding protein	0	0.88	33	-0.06	10	0.58	56	-0.24	13
SA1471	P66133	50S ribosomal protein L27	0	1.05	19	0.02	4	0.49	107	-0.31	5
SA1473	Q7A583	50S ribosomal protein L21	0	1.03	25	0.01	6	0.73	18	-0.14	4
SA1475	Q7A581	SA1475 protein	1	0.73	30	-0.13	5	0.63	44	-0.20	6
SA1476	Q7A580	Hypothetical protein SA1476	1	0.27	80	-0.56	2	0.09	96	-1.05	3
SA1487	Q7A575	Folylpolyglutamate synthase	0					0.64	59	-0.20	2

ORF number	AC	Description	TM domains	Proteomic experiment 1 (PE1)				Proteomic experiment 2 (PE2)			
				MRGR3/14-4	CV [%]	log(MRGR3/14-4)	nb. peptides	MRGR3/14-4	CV [%]	log(MRGR3/14-4)	nb. peptides
SA1491	P99096	Glutamate-1-semialdehyde 2,1-aminomutase 1 (EC 5.4.3.8) (GSA 1) (Glutamate-1-semialdehyde aminotransferase 1) (GSA-AT 1)	0					2.36	57	0.37	10
SA1497	P64071	Probable GTP-binding protein engB	0					0.51	65	-0.29	2
SA1499	P99080	Trigger factor (TF)	0	0.65	22	-0.19	5	0.93	20	-0.03	4
SA1502	P66108	50S ribosomal protein L20	0	1.10	37	0.04	3	0.78	59	-0.11	2
SA1504	P65140	Translation initiation factor IF-3	0	1.23	34	0.09	9	0.84	48	-0.08	5
SA1506	P67585	Threonyl-tRNA synthetase (EC 6.1.1.3) (Threonine--tRNA ligase) (ThrRS)	0	2.76	42	0.44	17	3.58	84	0.55	19
SA1507	Q7A568	Primosomal protein	0	0.62	21	-0.21	7	0.54	38	-0.27	10
SA1508	Q7A567	Chromosome replication initiation/membrane attachment protein	0	1.27		0.10	1	1.35	29	0.13	2
SA1509	P67316	Hypothetical UPF0168 protein SA1509	0	0.90		-0.05	1				
SA1510	P99067	Glyceraldehyde-3-phosphate dehydrogenase 2 (EC 1.2.1.12) (GAPDH 2)	0	1.19	49	0.08	7	1.49	53	0.17	9
SA1511	P63831	Dephospho-CoA kinase (EC 2.7.1.24) (Dephosphocoenzyme A kinase)	0					0.63	16	-0.20	3
SA1513	Q7A565	DNA polymerase I	0	0.63	56	-0.20	2	0.52		-0.28	1
SA1515	Q7A563	Alkaline phosphatase synthesis sensor protein	2					0.73		-0.14	1
SA1516	Q7A562	Alkaline phosphatase synthesis transcriptional regulatory protein	0	0.42	9	-0.38	2	0.66	32	-0.18	4
SA1517	P99167	Isocitrate dehydrogenase [NADP] (EC 1.1.1.42) (Oxalosuccinate decarboxylase) (IDH) (NADP+-specific ICDH) (IDP)	0	2.42	57	0.38	8	1.94	49	0.29	11
SA1518	Q7A561	Citrate synthase II	0	0.81	23	-0.09	7	3.84	40	0.58	11
SA1520	Q7A559	Pyruvate kinase	0	2.46	56	0.39	18	2.47	93	0.39	18
SA1521	P99165	6-phosphofructokinase (EC 2.7.1.11) (Phosphofructokinase) (Phosphohexokinase)	0	0.98	13	-0.01	2	1.63	33	0.21	4
SA1522	Q7A558	Acetyl-CoA carboxylase carboxyl transferase subunit alpha	0				1	0.50	26	-0.30	3
SA1523	Q7A557	Acetyl-CoA carboxylase transferase beta subunit	0	0.45	26	-0.35	3	0.65	10	-0.19	2
SA1526	Q7A555	Hypothetical protein SA1526	0					3.95	18	0.60	3
SA1527	Q7A554	Hypothetical protein SA1527	0					0.82	14	-0.08	4
SA1528	Q7A553	Hypothetical protein SA1528	0	1.13	22	0.05	12	0.84	16	-0.08	8
SA1532	Q7A551	Hypothetical protein SA1532	0	0.76	16	-0.12	7	2.03	53	0.31	9

				Proteomic experiment 1 (PE1)				Proteomic experiment 2 (PE2)			
ORF number	AC	Description	TM domains	MRGR3/14-4	CV [%]	log(MRGR3/14-4)	nb. peptides	MRGR3/14-4	CV [%]	log(MRGR3/14-4)	nb. peptides
SA1533	Q99TF2	Acetate kinase (EC 2.7.2.1) (Acetokinase)	0	2.06	43	0.31	13	2.67	45	0.43	17
SA1539	P64003	Septation ring formation regulator ezrA	1	0.79	37	-0.10	20	0.78	21	-0.11	13
SA1540.1	P66563	30S ribosomal protein S4	0	1.16	29	0.07	16	0.83	20	-0.08	11
SA1547	Q7A540	PTS system, N-acetylglucosamine-specific IIABC component	11	0.61	22	-0.21	1	0.51	29	-0.29	2
SA1548	Q7A539	SA1548 protein	0	0.67	38	-0.18	4	0.57	33	-0.24	6
SA1549	Q7A538	SA1549 protein	1	0.30	59	-0.52	20	0.13	42	-0.87	23
SA1553	Q7A535	Formyltetrahydrofolate synthetase	0	0.66	35	-0.18	9	2.11	48	0.32	11
SA1554	Q99TD1	Acetyl-CoA synthetase	1	0.84	23	-0.08	2	0.80	12	-0.10	7
SA1556	P64376	Acetoin utilization protein acuC	0	0.76		-0.12	1				
SA1557	P99175	Probable catabolite control protein A	0	1.38	47	0.14	5	1.48	46	0.17	9
SA1558	Q7A533	SA1558 protein	0	0.86	26	-0.06	3	0.57	37	-0.25	2
SA1559	Q7A532	SA1559 protein	1	1.30	36	0.11	8	0.96	42	-0.02	4
SA1560	Q7A531	SA1560 protein	1	1.14	40	0.06	12	0.77	25	-0.11	9
SA1561	P65475	UDP-N-acetylmuramate--L-alanine ligase (EC 6.3.2.8) (UDP-N-acetylmuramoyl-L-alanine synthetase)	0	1.73	86	0.24	5	1.02	39	0.01	6
SA1562	Q7A530	SA1562 protein	0	1.18	20	0.07	5	0.82	23	-0.09	2
SA1564	Q7A528	Hypothetical protein SA1564	0	0.94	2	-0.03	4	0.77	12	-0.11	3
SA1566	Q7A526	SA1566 protein	0	0.74	16	-0.13	3				
SA1567	Q7A525	Hypothetical protein SA1567	1	0.50	27	-0.30	2	0.45	26	-0.35	2
SA1571	P99090	D-alanine aminotransferase (EC 2.6.1.21) (D-aspartate aminotransferase) (D-amino acid aminotransferase) (D-amino acid transaminase) (DAAT)	0	2.57	20	0.41	6	2.20	51	0.34	9
SA1573	Q7A521	Hypothetical protein SA1573	0	0.66	18	-0.18	9	0.29	37	-0.54	7
SA1574	Q7A520	Pseudouridine synthase (EC 4.2.1.70) (Uracil hydrolyase)	0	0.53		-0.27	1	0.61	3	-0.21	2
SA1584	Q7A513	SA1584 protein	0	0.95	20	-0.02	7	0.68	23	-0.17	6
SA1585	Q7A512	SA1585 protein	1	0.57	40	-0.25	12	0.42	31	-0.38	12
SA1588	Q7A510	Riboflavin synthase alpha chain	0					0.18	76	-0.74	2
SA1590	Q7A508	Hypothetical protein SA1590	0					2.14		0.33	1
SA1593	Q99T94	Hypothetical protein SA1593	1	0.44	24	-0.36	12	0.29	24	-0.54	7
SA1607	Q7A4Z7	Hypothetical protein SA1607	1	0.47	27	-0.33	12	0.35	62	-0.46	10
SA1608	P66767	S-adenosylmethionine synthetase (EC 2.5.1.6) (Methionine adenosyltransferase) (AdoMet synthetase) (MAT)	0					1.82	51	0.26	5
SA1609	P51065	Phosphoenolpyruvate carboxykinase [ATP] (EC 4.1.1.49)	0	0.55	14	-0.26	4				

				Proteomic experiment 1 (PE1)				Proteomic experiment 2 (PE2)			
ORF number	AC	Description	TM domains	MRGR3/14-4	CV [%]	log(MRGR3/14-4)	nb. peptides	MRGR3/14-4	CV [%]	log(MRGR3/14-4)	nb. peptides
SA1639	Q7A4X4	Hypothetical protein SA1639	0	1.33	21	0.12	2	0.71	18	-0.15	2
SA1649	Q7A4W5	Hypothetical protein SA1649	0	0.58	25	-0.23	6	0.48	28	-0.32	5
SA1650	Q7A4W4	Protoporphyrinogen oxidase	0	0.85	20	-0.07	4	0.87	13	-0.06	4
SA1653	Q7A4W3	Signal transduction protein TRAP	0	0.37	62	-0.43	7	0.19	34	-0.71	5
SA1654	Q7A4W2	SA1654 protein	10	0.35	28	-0.46	3	0.50	32	-0.30	2
SA1655	Q7A4W1	SA1655 protein	0	0.52	26	-0.28	2	0.47	24	-0.33	3
SA1656	Q7A4W0	Hit-like protein involved in cell-cycle regulation	0					2.55	63	0.41	3
SA1657	Q7A4V9	Hypothetical protein SA1657	1	1.04	12	0.02	6	0.60	28	-0.22	3
SA1659	P60748	Foldase protein prsA precursor (EC 5.2.1.8)	0	0.16	62	-0.78	25	0.08	109	-1.12	12
SA1660	Q7A4V6	Cmp-binding-factor 1	0					0.78	61	-0.11	2
SA1661	Q7A4V5	Hypothetical protein SA1661	2	0.56	34	-0.25	24	0.41	167	-0.39	21
SA1662	Q7A4V4	Hypothetical protein SA1662	0	0.51	24	-0.29	9	0.61	48	-0.22	9
SA1664	Q7A4V2	Hypothetical protein SA1664	2	0.22	29	-0.66	2				
SA1665	Q7A4V1	Hypothetical protein SA1665	0	2.07	94	0.32	2				
SA1668	Q7A4U8	Hypothetical protein SA1668	0	0.58	24	-0.23	7	0.37	22	-0.43	5
SA1669	P64173	Fumarate hydratase class II (EC 4.2.1.2) (Fumarase C)	0					3.05	79	0.48	6
SA1671	Q7A4U5	Hypothetical protein SA1671	0	1.22	93	0.09	7	0.55	36	-0.26	5
SA1678	Q7A4T8	SA1678 protein	0					0.96		-0.02	1
SA1681	Q7A4T5	Glutamate-1-semialdehyde 2,1-aminomutase 2 (EC 5.4.3.8) (GSA 2) (Glutamate-1-semialdehyde aminotransferase 2) (GSA-AT 2)	0					1.83	57	0.26	6
SA1682	Q7A4T4	Hypothetical protein SA1682	4	0.39	98	-0.41	2				
SA1683	Q7A4T3	SA1683 protein	5					0.70	20	-0.15	4
SA1686	Q7A4T0	Hypothetical protein SA1686	4	0.45	25	-0.35	2	0.38	25	-0.42	3
SA1688	Q7A4S8	SA1688 protein	1	0.52	25	-0.28	4	0.55	29	-0.26	5
SA1690	P66003	Regulatory protein recX	0	1.21	16	0.08	3	0.60	33	-0.22	2
SA1691	Q7A4S6	SgtB protein	1	0.18	43	-0.74	9	0.08	87	-1.09	7
SA1694	Q99T03	Hypothetical protein SA1694	0	0.73	35	-0.14	2	1.16	122	0.07	3
SA1697	Q7A4S1	SA1697 protein	0	1.56		0.19	1	0.83	13	-0.08	1
SA1698	Q7A4S0	Hypothetical protein SA1698	1					0.13	41	-0.87	3
SA1699	Q99SZ8	SA1699 protein	6	1.30	18	0.11	4	0.88	29	-0.06	4
SA1700	Q7A4R9	Two-component response regulator	0	0.21	55	-0.67	3				
SA1701	Q99SZ7	Two-component sensor histidine kinase	2	0.16	66	-0.80	6	0.08	106	-1.09	7

				Proteomic experiment 1 (PE1)				Proteomic experiment 2 (PE2)			
ORF number	AC	Description	TM domains	MRGR3/14-4	CV [%]	log(MRGR3/14-4)	nb. peptides	MRGR3/14-4	CV [%]	log(MRGR3/14-4)	nb. peptides
SA1702	Q7A4R8	Hypothetical protein SA1702	4	0.10	42	-1.00	1	0.05	109	-1.27	2
SA1708	Q7A4R3	SA1708 protein	0	0.77	18	-0.11	3				
SA1709	Q7A4R2	SA1709 protein	0	0.43	29	-0.36	3	0.28	38	-0.55	3
SA1715	P99169	Aspartyl/glutamyl-tRNA(Asn/Gln) amidotransferase subunit B (EC 6.3.5.-) (Asp/Glu-ADT subunit B)	0					0.81	15	-0.09	3
SA1716	P63489	Glutamyl-tRNA(Gln) amidotransferase subunit A (EC 6.3.5.-) (Glu-ADT subunit A)	0					0.75		-0.13	1
SA1719	Q7A4Q6	Hypothetical protein SA1719	0	0.73	21	-0.14	4	0.56	18	-0.25	2
SA1721	P64319	ATP-dependent DNA helicase pcrA (EC 3.6.1.-)	0	1.08	114	0.03	7	0.59	38	-0.23	5
SA1724	Q7A4Q3	Adenylosuccinate lyase	0	1.03	31	0.01	7	3.84	52	0.58	7
SA1727	P61544	Hypothetical UPF0316 protein SA1727	3	0.71	19	-0.15	5	0.67	22	-0.18	5
SA1728	P99150	NH(3)-dependent NAD(+) synthetase (EC 6.3.1.5)	0					1.19		0.08	1
SA1729	Q7A4Q0	SA1729 protein	0					1.78	30	0.25	3
SA1730	Q99SX3	Nitric oxide synthase oxygenase (EC 1.-.-.-) (NOSoxy-like protein) (SANOS)	0	1.16	34	0.06	3	0.89	22	-0.05	3
SA1733	Q7A4P7	Hypothetical protein SA1733	0	1.09	51	0.04	3	0.48	26	-0.32	5
SA1735	P65753	Probable manganese-dependent inorganic pyrophosphatase (EC 3.6.1.1) (Pyrophosphate phospho-hydrolase) (PPase)	0					1.38	68	0.14	4
SA1736	Q7A4P5	Aldehyde dehydrogenase	0	0.51	58	-0.29	7	0.62	14	-0.21	5
SA1738.1	Q7A4P2	Hypothetical protein SAS056	0	0.89	27	-0.05	2				
SA1739	Q7A4P1	Hypothetical protein SA1739	0	1.00	12	0.00	3	0.50	19	-0.30	4
SA1743	Q7A4N7	Hypothetical protein SA1743	0					0.48		-0.32	1
SA1745	Q7A4N5	SA1745 protein	0	0.80	26	-0.10	12	0.36	145	-0.44	9
SA1747	Q7A4N3	SA1747 protein	0	0.68	34	-0.17	3	0.65	15	-0.19	3
SA1748	Q7A4N2	SA1748 protein	0	1.60	33	0.21	2				
SA1749	Q7A4N0	SA1749 protein	0					1.74	67	0.24	6
SA1751	P69775	Map protein [Precursor]	1	0.43	22	-0.37	2	0.38	26	-0.42	2
SA1812	Q7A4L0	SA1812 protein	0	1.83	38	0.26	8	0.97	11	-0.01	2
SA1813	Q99SN7	SA1813 protein	0	1.25	21	0.10	12	0.57	22	-0.25	13
SA1814	Q99SN6	Probable succinyl-diaminopimelate desuccinylase (EC 3.5.1.18) (SDAP)	0					2.27	24	0.36	2
SA1836	P99083	60 kDa chaperonin (Protein Cpn60) (groEL protein)	0	1.15	38	0.06	5	1.93	30	0.29	2
SA1841.1	P01506_WO	Delta-hemolysin precursor (Delta-toxin) (Delta-lysin)	0					1.80	43	0.26	2

ORF number	AC	Description	TM domains	Proteomic experiment 1 (PE1)				Proteomic experiment 2 (PE2)			
				MRGR3/14-4	CV [%]	log(MRGR3/14-4)	nb. peptides	MRGR3/14-4	CV [%]	log(MRGR3/14-4)	nb. peptides
SA1843	Q7A4I6	Accessory gene regulator C	4	0.95	20	-0.02	1	0.75	5	-0.12	2
SA1844	P13131	Accessory gene regulator protein A	0	1.61	19	0.21	6	1.38	13	0.14	3
SA1846	Q7A4I4	Sucrose-6-phosphate hydrolase	0	1.04		0.02	1	1.42	82	0.15	2
SA1847	Q7A4I3	Sucrose operon repressor	0					0.69	29	-0.16	3
SA1851	P60386	Redox-sensing transcriptional repressor rex	0					1.37	59	0.14	1
SA1852	Q7A4I0	Hypothetical ABC transporter ATP-binding protein	0	0.54	27	-0.27	4	0.47	11	-0.33	3
SA1853	Q7A4H9	SA1853 protein	2					0.82		-0.09	1
SA1854	Q7A4H8	SA1854 protein	0					2.17	25	0.34	2
SA1868	Q7A4H1	Hypothetical protein SA1868	0	1.46	30	0.17	7	1.20	34	0.08	3
SA1869	Q99SI8	RNA polymerase sigma factor	0	0.81	18	-0.09	2				
SA1870	P95843	Serine-protein kinase rsbW (EC 2.7.1.37)	0					2.58	7	0.41	2
SA1872	Q7A4H0	SigmaB regulation protein RsbU	0	0.47	38	-0.33	14	0.60	17	-0.22	7
SA1873	Q7A4G9	Hypothetical protein SA1873	0	1.06	32	0.03	2	1.11	19	0.05	2
SA1874	P63480	Alanine racemase 1 (EC 5.1.1.1)	0	2.90	49	0.46	2	0.87	56	-0.06	3
SA1882	Q7A4G3	Sensor protein KdpD	4	0.68	15	-0.17	4	0.48	15	-0.32	5
SA1883	Q7A4G2	KDP operon transcriptional regulatory protein KdpE	0	0.52	47	-0.28	5	0.47	1	-0.32	2
SA1885	Q7A4G0	SA1885 protein	0	0.74	41	-0.13	17	1.00	27	0.00	8
SA1886	Q7A4F9	UDP-N-acetylmuramoylalanyl-D-glutamyl-2, 6-diaminopimelate-D-alanyl-D-alanyl ligase	0	0.43	36	-0.36	4	0.45	21	-0.34	5
SA1887	P63892	D-alanine--D-alanine ligase (EC 6.3.2.4) (D-alanylalanine synthetase) (D-Ala-D-Ala ligase)	0					1.82	49	0.26	4
SA1891	P63801	Cardiolipin synthetase (EC 2.7.8.-) (Cardiolipin synthase) (CL synthase)	2	0.50	26	-0.30	4	0.30	49	-0.52	4
SA1893	P65629	Membrane protein oxaA precursor	6	0.48	19	-0.32	3	0.41	26	-0.38	3
SA1894	P66919	Thiamine-phosphate pyrophosphorylase (EC 2.5.1.3) (TMP pyrophosphorylase) (TMP-PPase) (Thiamine-phosphate synthase)	0	1.28	27	0.11	2	2.05	40	0.31	3
SA1896	P99124	Phosphomethylpyrimidine kinase (EC 2.7.4.7) (HMP-phosphate kinase) (HMP-P kinase)	0	0.71	19	-0.15	7	0.83	25	-0.08	7
SA1902	P84058	UDP-N-acetylglucosamine 1-carboxyvinyltransferase 1 (EC 2.5.1.7) (Enoylpyruvate transferase 1) (UDP-N-acetylglucosamine enolpyruvyl transferase 1) (EPT 1)	0	0.78	34	-0.11	5	0.70	37	-0.15	5

				Proteomic experiment 1 (PE1)				Proteomic experiment 2 (PE2)			
ORF number	AC	Description	TM domains	MRGR3/14-4	CV [%]	log(MRGR3/14-4)	nb. peptides	MRGR3/14-4	CV [%]	log(MRGR3/14-4)	nb. peptides
SA1904	P63665	ATP synthase epsilon chain (EC 3.6.3.14) (ATP synthase F1 sector epsilon subunit)	0	0.83	17	-0.08	3	0.66	27	-0.18	3
SA1905	P99112	ATP synthase beta chain (EC 3.6.3.14)	0	0.69	40	-0.16	30	0.63	25	-0.20	29
SA1906	Q7A4E8	ATP synthase gamma chain	0	0.77	24	-0.12	7	0.67	22	-0.17	7
SA1907	P99111	ATP synthase alpha chain (EC 3.6.3.14)	0	0.77	79	-0.12	22	0.75	22	-0.12	23
SA1908	P99109	ATP synthase delta chain (EC 3.6.3.14)	0	0.71	18	-0.15	6	0.67	30	-0.17	5
SA1909	Q7A4E7	ATP synthase B chain (EC 3.6.3.14)	1	0.73	38	-0.14	8	0.68	21	-0.17	1
SA1911	Q7A4E5	ATP synthase A chain	5					0.41	11	-0.39	1
SA1913	Q7A4E3	UDP-GlcNAc 2-epimerase	0	0.94	36	-0.03	3				
SA1914	P67396	Uracil phosphoribosyltransferase (EC 2.4.2.9) (UMP pyrophosphorylase) (UPRTase)	0	1.33	19	0.12	8	2.17	32	0.34	6
SA1915	P99091	Serine hydroxymethyltransferase (EC 2.1.2.1) (Serine methylase) (SHMT)	0	0.55	36	-0.26	4	2.74	67	0.44	10
SA1917	Q7A4E1	SA1917 protein	0	1.56		0.19	1				
SA1921	P65231	Thymidine kinase (EC 2.7.1.21)	0					1.60	60	0.20	2
SA1922	P66196	50S ribosomal protein L31 type B	0	1.06	38	0.03	6	0.61	37	-0.21	6
SA1923	Q99SD7	Transcription termination factor Rho	0	1.20	24	0.08	14	1.02	15	0.01	8
SA1924	Q7A4D8	SA1924 protein	0					1.80	9	0.25	2
SA1926	P65457	UDP-N-acetylglucosamine 1-carboxyvinyltransferase 2 (EC 2.5.1.7) (Enoylpyruvate transferase 2) (UDP-N-acetylglucosamine enolpyruvyl transferase 2) (EPT 2)	0					0.76	19	-0.12	4
SA1929	P99072	CTP synthase (EC 6.3.4.2) (UTP--ammonia ligase) (CTP synthetase)	0					1.33	61	0.12	4
SA1930	P66715	Probable DNA-directed RNA polymerase delta subunit (RNAP delta factor)	0					1.42	52	0.15	2
SA1933	Q7A4D3	Hypothetical protein SA1933	0	0.72	15	-0.15	3				
SA1935	Q99Q45	HmrA protein	0	0.94	56	-0.03	2				
SA1936	P65330	S-ribosylhomocysteine lyase (EC 4.4.1.21) (Autoinducer-2 production protein luxS) (AI-2 synthesis protein)	0	1.43	21	0.16	4	1.79	24	0.25	5
SA1937	Q7A4D1	Hypothetical protein SA1937	3	0.94	16	-0.03	1	0.47	12	-0.33	2
SA1938	Q7A4D0	Pyrimidine nucleoside phosphorylase	0	1.55	65	0.19	10	1.40	117	0.15	17
SA1942	Q7A4C7	Hypothetical protein SA1942	0	0.56	25	-0.25	6	0.33	24	-0.48	3
SA1943	Q7A4C6	Hypothetical protein SA1943	0	1.02	33	0.01	5	0.82	27	-0.09	6

ORF number	AC	Description	TM domains	Proteomic experiment 1 (PE1)				Proteomic experiment 2 (PE2)			
				MRGR3/14-4	CV [%]	log(MRGR3/14-4)	nb. peptides	MRGR3/14-4	CV [%]	log(MRGR3/14-4)	nb. peptides
SA1945	Q99SB6	Mannose-6-phosphate isomerase (EC 5.3.1.8) (PMI) (Phosphohexomutase) (Phosphomannose isomerase)	0					1.94		0.29	1
SA1946	Q7A4C4	Hypothetical protein SA1946	0					0.75	69	-0.12	2
SA1959	P64228	Glucosamine--fructose-6-phosphate aminotransferase [isomerizing] (EC 2.6.1.16) (Hexosephosphate aminotransferase) (D-fructose-6-phosphate amidotransferase) (GFAT) (L-glutamine-D-fructose-6-phosphate amidotransferase) (Glucosamine-6-phosphate synthase)	0	1.27	54	0.10	4	2.83	65	0.45	9
SA1965	P99087	Phosphoglucosamine mutase (EC 5.4.2.10)	0					0.33	6	-0.48	2
SA1966	Q7A4B0	Hypothetical protein SA1966	1	0.70	15	-0.16	7	0.54	25	-0.27	3
SA1967	Q7A4A9	Conserved hypothetoical protein	2	0.57	24	-0.24	2	0.55	13	-0.26	2
SA1969	Q7A4A8	SA1969 protein	0					0.92	48	-0.03	2
SA1975.1	Q99S93	Hypothetical protein SAS074	0	0.25	12	-0.60	1	0.37	14	-0.43	1
SA1979	Q7A499	SA1979 protein	0	0.68	27	-0.17	7	0.69	76	-0.16	4
SA1981	Q7A497	Hypothetical protein SA1981	0					0.57	16	-0.25	3
SA1983	Q7A495	Hypothetical protein SA1983	0					1.82	99	0.26	2
SA1984	Q53485	Alkaline shock protein 23	0	0.54	29	-0.26	6	1.09	37	0.04	6
SA1985	Q7A494	Hypothetical protein SA1985	2	0.95	22	-0.02	2	0.73	21	-0.14	2
SA1986	Q7A493	Hypothetical protein SA1986	2	1.46	34	0.16	12	0.83	31	-0.08	9
SA1987	Q99S82	SA1987 protein	12					0.25	8	-0.61	1
SA1989	Q7A492	SA1989 protein	0					1.41	20	0.15	3
SA1998	P67744	Lactose phosphotransferase system repressor	0	0.75	26	-0.13	2				
SA2005	Q7A484	Hypothetical protein SA2005	0	0.69	22	-0.16	6				
SA2016	P66646	30S ribosomal protein S9	0	1.44	20	0.16	6	0.75	51	-0.12	9
SA2017	Q7A473	50S ribosomal protein L13	0	1.06	59	0.02	14	0.39	140	-0.41	14
SA2020	Q7A471	Cobalt import ATP-binding protein cbiO 1	0	0.72	69	-0.14	2	0.50	47	-0.30	3
SA2022	Q7A469	50S ribosomal protein L17	0	1.55	56	0.19	6	0.14	223	-0.86	5
SA2023	P66706	DNA-directed RNA polymerase alpha chain (EC 2.7.7.6) (RNAP alpha subunit) (Transcriptase alpha chain) (RNA polymerase alpha subunit)	0	1.23	25	0.09	8	1.73	49	0.24	4
SA2024	P66357	30S ribosomal protein S11	0	1.23	22	0.09	7	0.81	35	-0.09	4
SA2025	P66388	30S ribosomal protein S13	0	1.19	32	0.08	10	1.00	17	0.00	6
SA2026	P65119	Translation initiation factor IF-1	0	1.06	2	0.02	2	0.75	25	-0.13	1

				Proteomic experiment 1 (PE1)				Proteomic experiment 2 (PE2)			
ORF number	AC	Description	TM domains	MRGR3/14-4	CV [%]	log(MRGR3/14-4)	nb. peptides	MRGR3/14-4	CV [%]	log(MRGR3/14-4)	nb. peptides
SA2028	Q7A468	Preprotein translocase secY subunit	10	0.41	41	-0.39	3	0.29	4	-0.53	2
SA2029	O06445	50S ribosomal protein L15	0	0.99	32	-0.01	9	0.50	99	-0.30	7
SA2030	O06444	50S ribosomal protein L30	0	0.93	30	-0.03	4	0.70	36	-0.16	3
SA2031	P66579	30S ribosomal protein S5	0	1.31	23	0.12	17	0.87	22	-0.06	10
SA2032	Q7A467	50S ribosomal protein L18	0	1.08	10	0.03	2	0.70	14	-0.15	1
SA2033	Q7A466	50S ribosomal protein L6	0	1.18	71	0.07	12	0.30	153	-0.52	5
SA2034	P66630	30S ribosomal protein S8	0	1.75	62	0.24	5	1.30	30	0.11	3
SA2035	Q7A465	50S ribosomal protein L5	0	1.67	115	0.22	13	0.67	32	-0.18	11
SA2036	P60735	50S ribosomal protein L24	0	1.00	21	0.00	1	0.63	18	-0.20	1
SA2037	Q7A463	50S ribosomal protein L14	0	0.76	35	-0.12	4				
SA2038	Q7A462	30S ribosomal protein S17	0	1.38	23	0.14	2	1.39	23	0.14	1
SA2039	P66173	50S ribosomal protein L29	0	1.10	18	0.04	3	0.79	25	-0.10	3
SA2040	Q7A461	50S ribosomal protein L16	0	1.00	24	0.00	3	0.69	12	-0.16	4
SA2041	P66553	30S ribosomal protein S3	0	1.14	44	0.06	12	0.85	27	-0.07	7
SA2042	Q7A460	50S ribosomal protein L22	0	1.12	54	0.05	9	0.55	44	-0.26	7
SA2043	P66494	30S ribosomal protein S19	0	1.73	37	0.24	9	0.90	39	-0.05	7
SA2044	P60432	50S ribosomal protein L2	0	0.96	31	-0.02	12	0.69	16	-0.16	11
SA2045	Q7A459	50S ribosomal protein L23	0	1.06	26	0.02	5	0.31	122	-0.50	6
SA2046	P61059	50S ribosomal protein L4	0	1.06	19	0.02	4	0.67	27	-0.17	6
SA2047	P60449	50S ribosomal protein L3	0	0.82	30	-0.08	2	0.69	15	-0.16	2
SA2048	P66334	30S ribosomal protein S10	0	1.49	18	0.17	5	1.05	17	0.02	3
SA2051	Q7A455	DNA topoisomerase III topB	0					0.72	22	-0.14	1
SA2056	Q7A448	SA2056 protein	12	0.60	25	-0.22	10	0.56	20	-0.25	8
SA2057	Q7A447	FmhB protein	0	0.83	27	-0.08	11	0.58	20	-0.24	7
SA2059	Q7A445	Hypothetical protein SA2059	0	0.76	35	-0.12	5	0.48		-0.32	1
SA2064	P65405	Probable molybdopterin-guanine dinucleotide biosynthesis protein A	0					0.34		-0.46	1
SA2067	Q7A440	Probable molybdopterin-guanine dinucleotide biosynthesis mobB	0					1.29		0.11	1
SA2074	Q99RZ3	Probable molybdate-binding protein	0	1.47	33	0.17	7	1.00	25	0.00	6
SA2079	Q7A433	SA2079 protein	0	0.57	19	-0.24	11	0.47	24	-0.33	8
SA2080	Q7A432	SA2080 protein	0					2.05		0.31	1
SA2085	Q7A429	Urease accessory protein UreE	0					0.40	9	-0.40	2

				Proteomic experiment 1 (PE1)				Proteomic experiment 2 (PE2)			
ORF number	AC	Description	TM domains	MRGR3/14-4	CV [%]	log(MRGR3/14-4)	nb. peptides	MRGR3/14-4	CV [%]	log(MRGR3/14-4)	nb. peptides
SA2089	Q7A425	SarR protein	0	2.23	50	0.35	2	1.21	29	0.08	2
SA2093	Q7A423	SsaA protein	0	0.26	66	-0.58	6	0.08	92	-1.12	18
SA2095	Q7A420	SA2095 protein	0	3.20	68	0.51	3	2.16	84	0.33	9
SA2096	Q7A419	Hypothetical protein SA2096	3	0.51	36	-0.29	2				
SA2098	Q7A417	SA2098 protein	0	0.51		-0.29	1	1.54	57	0.19	9
SA2099	Q7A416	SA2099 protein	0	0.61	41	-0.21	3	0.61	21	-0.22	2
SA2102	Q99RW4	SA2102 protein	0	0.28	38	-0.55	9	0.15	54	-0.82	21
SA2103	Q7A413	SA2103 protein	0	0.26	52	-0.59	16	0.08	77	-1.09	11
SA2105	Q7A411	Hypothetical protein SA2105	0	1.03	65	0.01	4	1.03	45	0.01	3
SA2106	Q7A410	SA2106 protein	7	0.37	32	-0.43	1	0.30		-0.52	1
SA2108	Q7A408	SA2108 protein	0	2.31	29	0.36	3	1.43	50	0.16	3
SA2111	Q7A403	SA2111 protein	0					0.69	8	-0.16	2
SA2113	Q7A401	Hypothetical protein SA2113	0	0.35	22	-0.45	2	0.30	38	-0.53	3
SA2114	Q7A400	PTS system, arbutin-like IIBC component	10	0.92	21	-0.03	6	0.92	16	-0.03	6
SA2115	Q7A3Z9	SA2115 protein	0	1.37	41	0.14	2	0.59		-0.23	1
SA2116	Q7A3Z8	Hypothetical protein SA2116	0					1.21	4	0.08	3
SA2117	Q7A3Z7	SA2117 protein	10					0.39		-0.41	1
SA2119	Q7A3Z5	SA2119 protein	0					1.19	66	0.08	3
SA2121	P64418	Imidazolonepropionase (EC 3.5.2.7) (Imidazolone-5-propionate hydrolase)	0	2.51		0.40	1				
SA2122	P67417	Urocanate hydratase (EC 4.2.1.49) (Urocanase) (Imidazolonepropionate hydrolase)	0	1.43	31	0.16	5	1.24	38	0.09	7
SA2128	Q7A3Z1	Hypothetical protein SA2128	0					0.70	30	-0.16	1
SA2132	Q99RT2	SA2132 protein	0	2.07	42	0.32	3	1.30	30	0.11	3
SA2133	Q7A3Y8	Hypothetical protein SA2133	4	0.70		-0.16	1				
SA2135	Q7A3Y6	SA2135 protein	11					0.57		-0.24	1
SA2137	Q7A3Y5	SA2137 protein	2					0.40	13	-0.40	2
SA2140	Q7A3Y2	SA2140 protein	0	0.91	39	-0.04	3	0.54	68	-0.27	3
SA2141	Q7A3Y1	Hypothetical protein SA2141	4	0.67	31	-0.18	2	0.52	17	-0.28	2
SA2147	Q7A3X5	TcaR transcription regulator	0	2.56	19	0.41	1	0.64	33	-0.19	1
SA2148	Q7A3X4	SA2148 protein	6	0.22		-0.67	1				
SA2155	P65422	Probable malate:quinone oxidoreductase 1 (EC 1.1.99.16) (Malate dehydrogenase [acceptor] 1) (MQO 1)	0	0.88	23	-0.06	11	0.34	139	-0.47	8

ORF number	AC	Description	TM domains	Proteomic experiment 1 (PE1)				Proteomic experiment 2 (PE2)			
				MRGR3/14-4	CV [%]	log(MRGR3/14-4)	nb. peptides	MRGR3/14-4	CV [%]	log(MRGR3/14-4)	nb. peptides
SA2156	Q7A3W7	SA2156 protein	13	0.84	16	-0.07	2				
SA2157	Q7A3W6	Hypothetical protein SA2157	0	0.55	18	-0.26	2	0.60	5	-0.23	2
SA2158	Q7A3W5	SA2158 protein	0	0.60	28	-0.22	8	0.43	97	-0.36	8
SA2162	Q7A3W1	SA2162 protein	0	0.38	25	-0.42	3	0.26	37	-0.59	3
SA2167	Q7A3V6	PTS system, sucrose-specific IIBC component	8					0.50	11	-0.30	3
SA2171	Q7A3V3	Hypothetical protein SA2171	1	0.85	30	-0.07	7	0.58	17	-0.23	5
SA2173	Q7A3V1	Hypothetical protein SA2173	1	0.63	26	-0.20	2				
SA2185	Q7A3U0	Respiratory nitrate reductase alpha chain	0	0.69	13	-0.16	2	0.81	7	-0.09	4
SA2188	Q99RM9	Nitrite reductase	0					1.22	51	0.08	6
SA2194	Q7A3T2	SA2194 protein	0	1.14	40	0.06	5	0.74	27	-0.13	3
SA2197	Q7A3S9	Hypothetical protein SA2197	0	0.44	2	-0.35	2	0.51	39	-0.29	5
SA2200	Q7A3S6	SA2200 protein	0	0.56	33	-0.25	3				
SA2201	Q7A3S5	SA2201 protein	2					0.33	29	-0.48	2
SA2202	Q99RL6	SA2202 protein	0	0.62	37	-0.21	14	0.57	29	-0.25	13
SA2204	P99153	2,3-bisphosphoglycerate-dependent phosphoglycerate mutase (EC 5.4.2.1) (Phosphoglyceromutase) (PGAM) (BPG-dependent PGAM) (dPGM)	0	0.46	21	-0.34	7				
SA2206	Q99RL2	IgG-binding protein SBI	0	0.71	38	-0.15	11	0.46	32	-0.34	8
SA2209	Q07226	Gamma-hemolysin component B precursor	0	1.42	28	0.15	2	0.48	48	-0.32	3
SA2220	Q7A3R2	Hypothetical protein SA2220	0					1.96	45	0.29	2
SA2221	Q7A3R1	Hypothetical protein SA2221	1	0.14	64	-0.86	10	0.03	107	-1.48	5
SA2224	Q7A3Q8	Hypothetical protein SA2224	0	0.68	17	-0.17	2	0.45	17	-0.35	4
SA2228	Q7A3Q7	SA2228 protein	12	0.58	77	-0.24	3	0.34	32	-0.47	3
SA2235	Q7A3Q0	Glycine betaine/carnitine/choline ABC transporter opuCC	1	0.41	23	-0.38	9	0.38	20	-0.42	6
SA2237	Q7A3P8	Glycine betaine/carnitine/choline ABC transporter opuCA	0	0.49	55	-0.31	9	0.26	26	-0.58	3
SA2240	Q7A3P6	SA2240 protein	0	1.24	17	0.09	2	0.91	19	-0.04	4
SA2247	Q7A3N9	Hypothetical protein SA2247	1	1.07	11	0.03	2	1.04	14	0.02	1
SA2255	Q7A3N0	Oligopeptide transporter putative substrate binding domain	0	1.37	27	0.14	2	1.03	28	0.01	4
SA2267	Q99RF4	Hypothetical protein SA2267	0	0.16	32	-0.80	2	0.14	2	-0.84	1
SA2277	Q7A3K9	Hypothetical protein SA2277	0	1.85	12	0.27	4	1.44	16	0.16	3
SA2288	Q7A3J9	UTP-glucose-1-phosphate uridyltransferase	0					1.26	4	0.10	2
SA2296	Q7A3J2	SA2296 protein	2	0.31	37	-0.50	5	0.20	38	-0.71	5
SA2297	Q7A3J1	SA2297 protein	0	0.69	99	-0.16	7	0.28	78	-0.56	6

				Proteomic experiment 1 (PE1)				Proteomic experiment 2 (PE2)			
ORF number	AC	Description	TM domains	MRGR3/14-4	CV [%]	log(MRGR3/14-4)	nb. peptides	MRGR3/14-4	CV [%]	log(MRGR3/14-4)	nb. peptides
SA2302	Q7A3I7	SA2302 protein	0	4.69	111	0.67	3	1.32	81	0.12	2
SA2304	Q7A3I5	Fructose-bisphosphatase	0	0.43	15	-0.37	3				
SA2311	Q7A3H8	SA2311 protein	0	0.73		-0.13	1	1.17	2	0.07	1
SA2312	P99116	D-lactate dehydrogenase (EC 1.1.1.28) (D-LDH) (D-specific D-2-hydroxyacid dehydrogenase)	0	1.86	81	0.27	2	3.82	62	0.58	8
SA2313	Q7A3H7	Hypothetical protein SA2313	0					0.63	20	-0.20	2
SA2316	Q7A3H4	Sortase	1	0.81	16	-0.09	2				
SA2323	Q7A3G7	Hypothetical protein SA2323	0	0.76	42	-0.12	10	0.58	31	-0.24	12
SA2326	Q7A3G4	PTS system, glucose-specific IIABC component	9	1.01		0.00					
SA2327	Q7A3G3	SA2327 protein	0	0.61	28	-0.21	16	0.35	31	-0.46	14
SA2332	Q7A3F8	SA2332 protein	0					0.11	76	-0.95	4
SA2336	Q7A3F4	ATP-dependent Clp proteinase chain clpL	0	0.44	20	-0.36	20	0.47	28	-0.33	14
SA2341	P99076	1-pyrroline-5-carboxylate dehydrogenase (EC 1.5.1.12) (P5C dehydrogenase)	0	0.32		-0.50	1	0.28	86	-0.55	5
SA2344	Q7A3E6	Copper-transporting ATPase copA	8	0.83	37	-0.08	5	0.89	12	-0.05	3
SA2348	Q7A3E2	Squalene desaturase	0	0.65	19	-0.19	6	0.38	36	-0.42	5
SA2351	Q7A3D9	SA2351 protein	0	0.84	26	-0.07	3	0.51	32	-0.29	12
SA2354	Q7A3D6	SA2354 protein	11					0.63		-0.20	1
SA2356	P99160	Immunodominant staphylococcal antigen A precursor	0					0.16	19	-0.80	2
SA2364	Q7A3C7	SA2364 protein	0					0.80		-0.10	1
SA2367	Q7A3C4	Hypothetical protein SA2367	0					0.23	8	-0.64	2
SA2374	Q7A3B7	Hypothetical protein SA2374	0					2.41	3	0.38	2
SA2375	Q7A3B6	SA2375 protein	0	0.97	31	-0.01	11	0.73	32	-0.14	9
SA2376	Q7A3B5	Hypothetical protein SA2376	1	0.47		-0.33	1				
SA2385	P04827	Streptomycin 3''-adenylyltransferase (EC 2.7.7.47) (AAD(9))	0	0.47	45	-0.33	7	0.17	130	-0.76	7
SA2391	P65659	Pantoate--beta-alanine ligase (EC 6.3.2.1) (Pantothenate synthetase) (Pantoate activating enzyme)	0					1.24	20	0.09	2
SA2395	P99119	L-lactate dehydrogenase 2 (EC 1.1.1.27) (L-LDH 2)	0					1.71	66	0.23	5
SA2399	P99117	Fructose-bisphosphate aldolase class I (EC 4.1.2.13) (FBP aldolase)	0					0.66	30	-0.18	2
SA2400	P99115	Probable malate:quinone oxidoreductase 2 (EC 1.1.99.16) (Malate dehydrogenase [acceptor] 2) (MQO 2)	0	0.56	63	-0.25	20	0.20	113	-0.70	19
SA2402	Q7A3A2	Acetate-CoA ligase (EC 6.2.1.1)	0	2.68	19	0.43	2	1.88	59	0.27	4

				Proteomic experiment 1 (PE1)				Proteomic experiment 2 (PE2)			
ORF number	AC	Description	TM domains	MRGR3/14-4	CV [%]	log(MRGR3/14-4)	nb. peptides	MRGR3/14-4	CV [%]	log(MRGR3/14-4)	nb. peptides
SA2405	P60337	Choline dehydrogenase (EC 1.1.99.1) (CHD) (CDH)	0	0.28	57	-0.55	9	0.15	52	-0.82	6
SA2410	Q7A395	Anaerobic ribonucleoside-triphosphate reductase	0	0.69	15	-0.16	2	0.87	23	-0.06	5
SA2413	Q7A392	Sulfite reductase flavoprotein (EC 1.8.1.2)	0	0.31	38	-0.51	5	0.25	31	-0.61	8
SA2417	Q7A388	SA2417 protein	2	0.44		-0.35	1	0.34		-0.47	1
SA2422	Q7A383	SA2422 protein	0	0.87	79	-0.06	3	0.43	22	-0.37	3
SA2424	Q7A381	SA2424 protein	0	1.33	53	0.12	4	1.30	22	0.12	6
SA2425	P99069	Carbamate kinase (EC 2.7.2.2)	0	3.64	51	0.56	5	4.59	77	0.66	10
SA2427	P65602	Ornithine carbamoyltransferase, catabolic (EC 2.1.3.3) (OTCase)	0					0.74	60	-0.13	2
SA2428	P63554	Arginine deiminase (EC 3.5.3.6) (ADI) (Arginine dihydrolase) (AD)	0	1.21	35	0.08	9	2.08	54	0.32	12
SA2431	Q7A377	Immunodominant antigen B	0	2.16	66	0.33	3	0.80	21	-0.10	5
SA2433	Q7A375	SA2433 protein	0	0.64	32	-0.19	3	0.58	1	-0.24	2
SA2434	Q7A374	SA2434 protein	8					0.82	17	-0.09	3
SA2436	Q7A372	SA2436 protein	6	0.78	12	-0.11	2	0.61	28	-0.22	3
SA2441	Q7A367	SA2441 protein	0					0.55		-0.26	1
SA2490	Q7A331	SA2490 protein	0	0.44	19	-0.36	4	0.94	9	-0.03	2
SA2498	Q7A321	SA2498 protein	0	0.79	78	-0.10	1	1.36	1	0.13	1
SA2500	P64230	Glucose inhibited division protein A	0					0.39	14	-0.40	3
SAA0003	Q5HJZ6	Plasmid recombination enzyme	0	0.80	9	-0.10	2	0.60		-0.22	1
SAP010	Q9AC80	Beta-lactamase	0	1.78	41	0.25	11	0.88	27	-0.06	7
SAP014	Q9AC76	Tnp protein	0					0.42	45	-0.38	2
SAR0080	Q6GKM5	Hypothetical protein	0	1.22	46	0.09	12	0.83	17	-0.08	6
SAR0158	Q6GKE9	Capsular polysaccharide synthesis enzyme	0	0.37	34	-0.43	3	0.15	27	-0.83	2
SAR0160	Q6GKE7	Capsular polysaccharide synthesis enzyme	0					0.19	23	-0.71	4
SAR0723	Q6GIX1	Cation-transporting ATPase	6	0.58		-0.24	1	0.97		-0.01	1
SAR2030	Q6GFB8	MHC class II analog	0	0.50	15	-0.30	4				
SAS0971	Q6GAH7	Putative glycosyl transferases	0	6.09	84	0.78	9	5.65	40	0.75	5
SAS1741	Q6G8B6	Putative lantibiotic transport ATP-binding protein	0					1.18	37	0.07	5
SAS1743	Q6G8B4	Putative lantibiotic modifying enzyme	0					1.56	12	0.19	2
SAS1745	Q6G8B2	Putative lantibiotic biosynthesis protein	0					0.56	1	-0.25	2
SAS1897	Q6G7W2	Hypothetical phage protein	0	0.65		-0.19	1	0.49	17	-0.31	3
SAV0413	Q932H3	Hypothetical protein	0	2.08	49	0.32	2	0.94	23	-0.03	4

				Proteomic experiment 1 (PE1)				Proteomic experiment 2 (PE2)			
ORF number	AC	Description	TM domains	MRGR3/14-4	CV [%]	log(MRGR3/14-4)	nb. peptides	MRGR3/14-4	CV [%]	log(MRGR3/14-4)	nb. peptides
SAV0414	Q932H2	Hypothetical protein	0					2.16	46	0.33	4
SAV0849	Q932C1	Hypothetical protein	0	1.11	66	0.05	4	0.86	32	-0.07	3
SAV1938	Q99QS1	Map protein [Precursor]	0	0.46	17	-0.34	8	0.18	40	-0.74	10
SAVP026	P14507	Bifunctional AAC/APH	0					4.06	24	0.61	6
SE0080	Q8CQH3	Regulatory protein	0	0.98	40	-0.01	5	1.17	77	0.07	6
