# Supplementary material for: Exploring glycopeptide-resistance in Staphylococcus aureus: a combined proteomics and transcriptomics approach for the identification of resistance-related markers
Source: BMC Genomics. 2006 Nov 22;7:296. doi: 10.1186/1471-2164-7-296 (PMC1687195; doi:10.1186/1471-2164-7-296)
Supplement: Additional file 5 — Relative quantification of all identified proteins and trend of mRNA expression obtained by microarray for the comparison between strains MRGR3 and 14-4. Table showing the comparison between protein expression and trend of mRNA expression measured by microarray for strain MRGR3 (parental) strain and 14-4 (GISA) [file 1471-2164-7-296-S5.pdf]

Additional file 5 : Relative quantification of all identified proteins and trend of mRNA expression obtained by microarray for the comparison between strains MRGR3 and 14-4

ORF number	AC	Description	1:TM domains	PR1:log (14-4/MRGR3)	PR2:log (14-4/MRGR3)	mean	expression
SA0013	Q7A8E3	Hypothetical protein SA0013	2	0.34	0.43	0.39	up
SA0108	Q7A872	SarH1 protein	0	-0.42	-0.32	-0.37	down
SA0144	Q7A842	Capsular polysaccharide synthesis enzyme Cap5A	2	0.45	0.98	0.71	up
SA0147	Q7A840	Capsular polysaccharide synthesis enzyme Cap5D	4	0.52	0.83	0.68	up
SA0155	Q7A832	Capsular polysaccharide synthesis enzyme Cap5L	0	0.39	0.84	0.62	up
SA0156	Q7A831	Capsular polysaccharide synthesis enzyme Cap5M	1	0.51	0.81	0.66	up
SA0159	Q7A828	Capsular polysaccharide synthesis enzyme Cap5P	0	0.27	0.33	0.30	up
SA0184	Q7A806	Hypothetical protein SA0184	0	-0.18	-0.25	-0.21	down
SA0189	Q7A801	Probable type I restriction enzyme restriction chain	0	-0.45	-0.47	-0.46	down
SA0229	Q7A7W6	SA0229 protein	0	0.40	0.52	0.46	up
SA0244	Q7A7V1	SA0244 protein	0	0.35	0.60	0.47	up
SA0246	Q7A7U9	SA0246 protein	0	-0.45	-0.53	-0.49	down
SA0248	Q7A7U7	SA0248 protein	0	0.28	0.39	0.34	up
SA0342	Q7A7L2	SA0342 protein	0	-0.19	-0.59	-0.39	down
SA0368	Q7A7I9	SA0368 protein	9	0.48	0.36	0.42	up
SA0422	Q7A7E1	SA0422 protein	0	0.37	0.39	0.38	up
SA0448	P67579	Methionyl-tRNA synthetase (EC 6.1.1.10) (Methionine--tRNA ligase) (MetRS)	0	-0.45	-0.49	-0.47	down
SA0456	Q7A7B5	SpoVG protein	0	-0.19	-0.27	-0.23	down
SA0469	Q7A7A5	Cell-division protein	2	0.29	0.48	0.39	up
SA0480	Q7A799	CtsR protein	0	0.36	0.37	0.37	up
SA0485	Q7A795	Hypothetical protein SA0485	4	0.25	0.39	0.32	up
SA0511	Q7A788	SA0511 protein	0	-0.52	-0.24	-0.38	down
SA0536.1	Q99W32	Hypothetical protein SAS016	0	1.12	0.94	1.03	up
SA0537	Q7A765	SA0537 protein	0	-0.37	-0.47	-0.42	down
SA0570	Q7A735	Hypothetical protein SA0570	0	0.38	0.80	0.59	up
SA0591	Q7A715	Hypothetical protein SA0591	6	0.91	1.29	1.10	up
SA0593	Q7A713	Teichoic acids export ATP-binding protein tagH (EC 3.6.3.40)	0	0.41	0.30	0.35	up
SA0595	Q7A712	Teichoic acid biosynthesis protein B	0	0.35	0.40	0.37	up
SA0599	Q7A708	ATP-binding cassette transporter A	6	0.29	0.31	0.30	up
SA0618	Q7A6Z0	Hypothetical protein SA0618	0	0.24	0.49	0.37	up

ORF number	AC	Description	1:TM domains	PE:log (14-4/MRGR3)	PE2:log (14-4/MRGR3)	mean	expression
SA0678	Q7A6T7	SA0678 protein	6	0.33	0.32	0.32	up
SA0713	P67425	UvrABC system protein B (UvrB protein) (Excinuclease ABC subunit B)	0	-0.29	-0.31	-0.30	down
SA0721	Q7A6Q7	Hypothetical protein SA0721	0	-0.21	-0.31	-0.26	down
SA0731	P99088	Enolase (EC 4.2.1.11) (2-phosphoglycerate dehydratase) (2-phospho-D-glycerate hydro-lyase)	0	0.41	0.60	0.50	up
SA0741	Q7A6P6	Hypothetical protein SA0741	0	0.42	0.43	0.42	up
SA0744	Q7A6P4	Extracellular ECM and plasma binding protein	0	0.47	0.38	0.43	up
SA0771	Q7A6M0	Hypothetical protein SA0771	0	0.27	0.37	0.32	up
SA0802	Q7A6J4	SA0802 protein	0	0.54	0.54	0.54	up
SA0813	P60675	Na(+)/H(+) antiporter subunit A (Mnh complex subunit A)	21	0.35	0.61	0.48	up
SA0826	P72365	Signal peptidase IB	1	0.60	0.89	0.75	up
SA0831	Q7A6H1	Coenzyme A disulfide reductase (EC 1.8.1.14) (CoA-disulfide reductase) (CoADR)	0	-0.48	-0.46	-0.47	down
SA0843	Q7A6F8	3-oxoacyl-synthase	0	-0.47	-0.52	-0.49	down
SA0868	Q7A6D9	SA0868 protein	12	0.45	0.44	0.44	up
SA0873	Q7A6D4	Hypothetical protein SA0873	0	-0.41	-0.58	-0.50	down
SA0876	P65480	UDP-N-acetylmuramoylalanyl-D-glutamate--2,6-diaminopimelate ligase (EC 6.3.2.13) (UDP-N-acetylmuramyl-tripeptide synthetase) (Meso-diaminopimelate-adding enzyme) (UDP-MurNAc-tripeptide synthetase)	0	0.45	0.47	0.46	up
SA0909	Q7A6A2	FmtA, autolysis and methicillin resistant-related protein	1	0.62	0.78	0.70	up
SA0917	Q7A695	PurK protein	0	-0.33	-0.39	-0.36	down
SA0931	Q7A689	Hypothetical protein SA0931	10	0.41	0.56	0.48	up
SA0937	Q7A685	SA0937 protein	9	0.39	0.40	0.40	up
SA0939	Q7A683	Hypothetical protein SA0939	0	0.39	0.49	0.44	up
SA0943	Q7A681	Hypothetical protein SA0943	0	0.38	0.36	0.37	up
SA0946	Q59822	Dihydrolipoyl dehydrogenase (EC 1.8.1.4)	0	-0.31	-0.52	-0.42	down
SA0954	Q7A675	Hypothetical protein SA0954	3	0.25	0.49	0.37	up
SA0969	Q7A661	SA0969 protein	1	0.41	0.58	0.50	up
SA0977	Q7A655	Cell surface protein	1	-0.44	-0.29	-0.36	down
SA1000	Q7A639	SA1000 protein	0	0.45	1.08	0.77	up
SA1019	Q99UT4	Hypothetical protein SA1019	0	0.41	0.33	0.37	up
SA1024	Q7A619	Penicillin-binding protein 1	1	0.26	0.39	0.33	up

ORF number	AC	Description	1:TM domains	PE:log (14-4/MRGR3)	PE2:log (14-4/MRGR3)	mean	expression
SA1029	P45498	Cell division protein ftsZ	0	-0.22	-0.39	-0.30	down
SA1040	Q7A610	Pseudouridine synthase (EC 4.2.1.70) (Uracil hydrolyase)	0	0.40	0.54	0.47	up
SA1043	P65618	Aspartate carbamoyltransferase (EC 2.1.3.2) (Aspartate transcarbamylase) (ATCase)	0	-0.35	-0.38	-0.36	down
SA1045	P99147	Carbamoyl-phosphate synthase small chain (EC 6.3.5.5) (Carbamoyl-phosphate synthetase glutamine chain)	0	-0.60	-0.42	-0.51	down
SA1046	P63740	Carbamoyl-phosphate synthase large chain (EC 6.3.5.5) (Carbamoyl-phosphate synthetase ammonia chain)	0	-0.50	-0.55	-0.53	down
SA1053	P66726	DNA-directed RNA polymerase omega chain (EC 2.7.7.6) (RNAP omega subunit) (Transcriptase omega chain) (RNA polymerase omega subunit)	0	-0.25	-0.49	-0.37	down
SA1063	Q7A5Z8	Protein kinase	1	0.33	0.38	0.35	up
SA1069	Q7A5Z4	Conserved hypotehtical protein	0	-0.17	-0.25	-0.21	down
SA1073	Q7A5Z3	Malonyl CoA-acyl carrier protein transacylase	0	-0.54	-0.24	-0.39	down
SA1088	P99071	Succinyl-CoA synthetase beta chain (EC 6.2.1.5) (SCS-beta)	0	-0.25	-0.24	-0.25	down
SA1105	P63333	Hypothetical zinc metalloprotease SA1105 (EC 3.4.24.-)	5	0.48	0.38	0.43	up
SA1117	Q7A5X7	Polyribonucleotide nucleotidyltransferase	0	0.31	0.27	0.29	up
SA1129	P67278	Hypothetical UPF0144 protein SA1129	1	0.35	0.48	0.41	up
SA1133	Q7A5W4	Hypothetical protein SA1133	0	-0.33	-0.45	-0.39	down
SA1142	Q7A5V7	Aerobic glycerol-3-phosphate dehydrogenase	0	0.27	0.53	0.40	up
SA1155	Q7A5U5	SA1155 protein	2	0.36	0.31	0.33	up
SA1183	Q99UC9	Glycine betaine transporter	11	0.36	0.34	0.35	up
SA1186	Q7A5S4	Hypothetical protein SA1186	0	0.54	0.58	0.56	up
SA1190	Q7A5S3	Amino acid carrier protein	9	0.39	0.31	0.35	up
SA1192	Q7A5S0	Hypothetical protein SA1192	8	0.39	0.46	0.43	up
SA1193	Q7A5R9	Oxacillin resistance-related FmtC protein	14	0.46	0.33	0.40	up
SA1195	Q99Q02	Peptide methionine sulfoxide reductase regulator MsrR	1	0.58	0.62	0.60	up
SA1238	P60108	TelA-like protein SA1238	0	0.33	0.43	0.38	up
SA1244	Q7A5N4	Dihydrolipoamide succinyltransferase	0	-0.69	-0.62	-0.65	down
SA1253	Q7A5M9	Probable carboxy-terminal processing proteinase ctpA	1	0.59	0.64	0.61	up
SA1256	P99065	Peptide methionine sulfoxide reductase msrB (EC 1.8.4.6)	0	0.60	0.79	0.69	up
SA1257	P65446	Peptide methionine sulfoxide reductase msrA 2 (EC 1.8.4.6) (Protein-methionine-S-oxide reductase 2) (Peptide Met(O) reductase 2)	0	0.92	0.80	0.86	up

ORF number	AC	Description	1:TM domains	PE:log (14-4/MRGR3)	PE2:log (14-4/MRGR3)	mean	expression
SA1271	Q7A5L8	SA1271 protein	0	-0.51	-0.68	-0.59	down
SA1279	Q7A5L1	Hypothetical protein SA1279	0	0.41	0.65	0.53	up
SA1283	Q7A5K8	PBP2	1	0.35	0.85	0.60	up
SA1293	Q7A5J9	Hypothetical protein SA1293	4	0.49	0.61	0.55	up
SA1303	P67062	Menaquinone biosynthesis methyltransferase ubiE (EC 2.1.1.-)	0	0.35	0.44	0.40	up
SA1307	P64060	GTP-binding protein engA	0	-0.46	-0.44	-0.45	down
SA1361	Q7A5F0	Hypothetical protein SA1361	0	0.34	0.36	0.35	up
SA1363	Q7A5E8	Hypothetical protein SA1363	0	0.28	0.37	0.33	up
SA1390	Q99TT5	RNA polymerase sigma factor rpoD	0	0.41	0.30	0.36	up
SA1417	Q7A5B8	Late competence operon required for DNA binding and uptake comEB	0	0.41	0.30	0.36	up
SA1453	Q7A592	Hypothetical protein SA1453	0	0.36	0.30	0.33	up
SA1464	Q7A585	Hypothetical protein SA1464	1	0.30	0.80	0.55	up
SA1476	Q7A580	Hypothetical protein SA1476	1	0.56	1.05	0.80	up
SA1506	P67585	Threonyl-tRNA synthetase (EC 6.1.1.3) (Threonine--tRNA ligase) (ThrRS)	0	-0.44	-0.55	-0.50	down
SA1517	P99167	Isocitrate dehydrogenase [NADP] (EC 1.1.1.42) (Oxalosuccinate decarboxylase) (IDH) (NADP+-specific ICDH) (IDP)	0	-0.38	-0.29	-0.34	down
SA1520	Q7A559	Pyruvate kinase	0	-0.39	-0.39	-0.39	down
SA1533	Q99TF2	Acetate kinase (EC 2.7.2.1) (Acetokinase)	0	-0.31	-0.43	-0.37	down
SA1549	Q7A538	SA1549 protein	1	0.52	0.87	0.70	up
SA1567	Q7A525	Hypothetical protein SA1567	1	0.30	0.35	0.33	up
SA1571	P99090	D-alanine aminotransferase (EC 2.6.1.21) (D-aspartate aminotransferase) (D-amino acid aminotransferase) (D-amino acid transaminase) (DAAT)	0	-0.41	-0.34	-0.38	down
SA1585	Q7A512	SA1585 protein	1	0.25	0.38	0.31	up
SA1593	Q99T94	Hypothetical protein SA1593	1	0.36	0.54	0.45	up
SA1607	Q7A4Z7	Hypothetical protein SA1607	1	0.33	0.46	0.39	up
SA1653	Q7A4W3	Signal transduction protein TRAP	0	0.43	0.71	0.57	up
SA1654	Q7A4W2	SA1654 protein	10	0.46	0.30	0.38	up
SA1655	Q7A4W1	SA1655 protein	0	0.28	0.33	0.31	up
SA1659	P60748	Foldase protein prsA precursor (EC 5.2.1.8)	0	0.78	1.12	0.95	up
SA1661	Q7A4V5	Hypothetical protein SA1661	2	0.25	0.39	0.32	up
SA1686	Q7A4T0	Hypothetical protein SA1686	4	0.35	0.42	0.38	up

ORF number	AC	Description	1:TM domains	PE:log (14-4/MRGR3)	PE2:log (14-4/MRGR3)	mean	expression
SA1691	Q7A4S6	SgtB protein	1	0.74	1.09	0.92	up
SA1701	Q99SZ7	Two-component sensor histidine kinase	2	0.80	1.09	0.94	up
SA1702	Q7A4R8	Hypothetical protein SA1702	4	1.00	1.27	1.13	up
SA1709	Q7A4R2	SA1709 protein	0	0.36	0.55	0.46	up
SA1751	P69775	Map protein [Precursor]	1	0.37	0.42	0.40	up
SA1852	Q7A4I0	Hypothetical ABC transporter ATP-binding protein	0	0.27	0.33	0.30	up
SA1883	Q7A4G2	KDP operon transcriptional regulatory protein KdpE	0	0.28	0.32	0.30	up
SA1886	Q7A4F9	UDP-N-acetylmuramoylalanyl-D-glutamyl-2, 6-diaminopimelate-D-alanyl-D-alanyl ligase	0	0.36	0.34	0.35	up
SA1891	P63801	Cardiolipin synthetase (EC 2.7.8.-) (Cardiolipin synthase) (CL synthase)	2	0.30	0.52	0.41	up
SA1893	P65629	Membrane protein oxaA precursor	6	0.32	0.38	0.35	up
SA1942	Q7A4C7	Hypothetical protein SA1942	0	0.25	0.48	0.37	up
SA1975.1	Q99S93	Hypothetical protein SAS074	0	0.60	0.43	0.52	up
SA2028	Q7A468	Preprotein translocase secY subunit	10	0.39	0.53	0.46	up
SA2079	Q7A433	SA2079 protein	0	0.24	0.33	0.29	up
SA2093	Q7A423	SsaA protein	0	0.58	1.12	0.85	up
SA2095	Q7A420	SA2095 protein	0	-0.51	-0.33	-0.42	down
SA2102	Q99RW4	SA2102 protein	0	0.55	0.82	0.68	up
SA2103	Q7A413	SA2103 protein	0	0.59	1.09	0.84	up
SA2106	Q7A410	SA2106 protein	7	0.43	0.52	0.47	up
SA2113	Q7A401	Hypothetical protein SA2113	0	0.45	0.53	0.49	up
SA2162	Q7A3W1	SA2162 protein	0	0.42	0.59	0.50	up
SA2197	Q7A3S9	Hypothetical protein SA2197	0	0.35	0.29	0.32	up
SA2221	Q7A3R1	Hypothetical protein SA2221	1	0.86	1.48	1.17	up
SA2228	Q7A3Q7	SA2228 protein	12	0.24	0.47	0.35	up
SA2235	Q7A3Q0	Glycine betaine/carnitine/choline ABC transporter opuCC	1	0.38	0.42	0.40	up
SA2237	Q7A3P8	Glycine betaine/carnitine/choline ABC transporter opuCA	0	0.31	0.58	0.45	up
SA2267	Q99RF4	Hypothetical protein SA2267	0	0.80	0.84	0.82	up
SA2296	Q7A3J2	SA2296 protein	2	0.50	0.71	0.61	up
SA2312	P99116	D-lactate dehydrogenase (EC 1.1.1.28) (D-LDH) (D-specific D-2-hydroxyacid dehydrogenase)	0	-0.27	-0.58	-0.43	down
SA2336	Q7A3F4	ATP-dependent Clp proteinase chain clpL	0	0.36	0.33	0.34	up

ORF number	AC	Description	1:TM domains	PE:log (14-4/MRGR3)	PE2:log (14-4/MRGR3)	mean	expression
SA2341	P99076	1-pyrroline-5-carboxylate dehydrogenase (EC 1.5.1.12) (P5C dehydrogenase)	0	0.50	0.55	0.52	up
SA2385	P04827	Streptomycin 3''-adenylyltransferase (EC 2.7.7.47) (AAD(9))	0	0.33	0.76	0.54	up
SA2400	P99115	Probable malate:quinone oxidoreductase 2 (EC 1.1.99.16) (Malate dehydrogenase [acceptor] 2) (MQO 2)	0	0.25	0.70	0.48	up
SA2402	Q7A3A2	Acetate-CoA ligase (EC 6.2.1.1)	0	-0.43	-0.27	-0.35	down
SA2405	P60337	Choline dehydrogenase (EC 1.1.99.1) (CHD) (CDH)	0	0.55	0.82	0.68	up
SA2413	Q7A392	Sulfite reductase flavoprotein (EC 1.8.1.2)	0	0.51	0.61	0.56	up
SA2417	Q7A388	SA2417 protein	2	0.35	0.47	0.41	up
SA2425	P99069	Carbamate kinase (EC 2.7.2.2)	0	-0.56	-0.66	-0.61	down
SAR0158	Q6GKE9	Capsular polysaccharide synthesis enzyme	0	0.43	0.83	0.63	up
SAS0971	Q6GAH7	Putative glycosyl transferases	0	-0.78	-0.75	-0.77	down
SAV1938	Q99QS1	Map protein [Precursor]	0	0.34	0.74	0.54	up
