# Supplementary material for: Exploring glycopeptide-resistance in Staphylococcus aureus: a combined proteomics and transcriptomics approach for the identification of resistance-related markers
Source: BMC Genomics. 2006 Nov 22;7:296. doi: 10.1186/1471-2164-7-296 (PMC1687195; doi:10.1186/1471-2164-7-296)
Supplement: Additional file 6 — Relative quantification of all identified proteins and trend of mRNA expression obtained by microarray for the comparison between strains 14-4 and 14-4Rev. Table showing the comparison between protein expression and trend of mRNA expression measured by microarray for strain 14-4 (GISA) strain and 14-4Rev (susceptible revertant of GISA 14-4) [file 1471-2164-7-296-S6.pdf]

Additional file 6 : Relative quantification of all identified proteins and trend of mRNA expression obtained by microarray for the comparison between strains 14-4 and 14-4Rev

ORF number	AC	Description	TM domains	PR1:log(14-4/14-4R)	PR2:log(14-4/14-4R)	mean	expression
SA0022	Q99XE9	SA0022 protein	0	-0.45	-0.34	-0.39	down
SA0108	Q7A872	SarH1 protein	0	-0.38	-0.40	-0.39	down
SA0147	Q7A840	Capsular polysaccharide synthesis enzyme Cap5D	4	0.32	0.46	0.39	up
SA0155	Q7A832	Capsular polysaccharide synthesis enzyme Cap5L	0	0.29	0.50	0.39	up
SA0159	Q7A828	Capsular polysaccharide synthesis enzyme Cap5P	0	0.20	0.37	0.29	up
SA0189	Q7A801	Probable type I restriction enzyme restriction chain	0	-0.41	-0.33	-0.37	down
SA0219	Q7A7X5	Formate acetyltransferase activating enzyme	0	-0.31	-0.36	-0.34	down
SA0244	Q7A7V1	SA0244 protein	0	0.22	0.34	0.28	up
SA0246	Q7A7U9	SA0246 protein	0	-0.47	-0.41	-0.44	down
SA0342	Q7A7L2	SA0342 protein	0	-0.38	-0.67	-0.52	down
SA0422	Q7A7E1	SA0422 protein	0	0.28	0.23	0.26	up
SA0448	P67579	Methionyl-tRNA synthetase (EC 6.1.1.10) (Methionine--tRNA ligase) (MetRS)	0	-0.36	-0.48	-0.42	down
SA0456	Q7A7B5	SpoVG protein	0	-0.31	-0.38	-0.35	down
SA0469	Q7A7A5	Cell-division protein	2	0.23	0.24	0.23	up
SA0485	Q7A795	Hypothetical protein SA0485	4	0.29	0.24	0.26	up
SA0511	Q7A788	SA0511 protein	0	-0.61	-0.36	-0.49	down
SA0537	Q7A765	SA0537 protein	0	-0.58	-0.47	-0.53	down
SA0562	Q7A742	Alcohol dehydrogenase I	0	-0.24	-0.61	-0.42	down
SA0570	Q7A735	Hypothetical protein SA0570	0	0.23	0.36	0.30	up
SA0591	Q7A715	Hypothetical protein SA0591	6	0.37	0.30	0.34	up
SA0595	Q7A712	Teichoic acid biosynthesis protein B	0	0.17	0.26	0.21	up
SA0713	P67425	UvrABC system protein B (UvrB protein) (Excinuclease ABC subunit B)	0	-0.32	-0.44	-0.38	down
SA0731	P99088	Enolase (EC 4.2.1.11) (2-phosphoglycerate dehydratase) (2-phospho-D-glycerate hydro-lyase)	0	0.30	0.41	0.36	up
SA0774	Q7A6L7	SA0774 protein	0	-0.52	-0.29	-0.41	down
SA0802	Q7A6J4	SA0802 protein	0	0.29	0.28	0.29	up
SA0826	P72365	Signal peptidase IB	1	0.32	0.47	0.40	up
SA0831	Q7A6H1	Coenzyme A disulfide reductase (EC 1.8.1.14) (CoA-disulfide reductase) (CoADR)	0	-0.53	-0.53	-0.53	down
SA0843	Q7A6F8	3-oxoacyl-synthase	0	-0.62	-0.46	-0.54	down

ORF number	AC	Description	TM domains	PE1:log(14-4/REV)	PE2:log(14-4/REV)	mean	expression
SA0868	Q7A6D9	SA0868 protein	12	0.36	0.28	0.32	up
SA0873	Q7A6D4	Hypothetical protein SA0873	0	-0.41	-0.51	-0.46	down
SA0876	P65480	UDP-N-acetylmuramoylalanyl-D-glutamate--2,6-diaminopimelate ligase (EC 6.3.2.13) (UDP-N-acetylmuramyl-tripeptide synthetase) (Meso-diaminopimelate-adding enzyme) (UDP-MurNAc-tripeptide synthetase)	0	0.31	0.30	0.30	up
SA0909	Q7A6A2	FmtA, autolysis and methicillin resistant-related protein	1	0.27	0.51	0.39	up
SA0917	Q7A695	PurK protein	0	-0.32	-0.42	-0.37	down
SA0931	Q7A689	Hypothetical protein SA0931	10	0.31	0.45	0.38	up
SA0939	Q7A683	Hypothetical protein SA0939	0	0.25	0.30	0.27	up
SA0943	Q7A681	Hypothetical protein SA0943	0	0.27	0.25	0.26	up
SA0943.1	Q820A6	Pyruvate dehydrogenase E1 component, alpha subunit (EC 1.2.4.1)	0	-0.51	-0.37	-0.44	down
SA0944	Q9L6H5	Pyruvate dehydrogenase E1 component, beta subunit (EC 1.2.4.1)	0	-0.45	-0.45	-0.45	down
SA0945	P65636	Dihydrolipoyllysine-residue acetyltransferase component of pyruvate dehydrogenase complex (EC 2.3.1.12) (E2) (Dihydrolipoamide acetyltransferase component of pyruvate dehydrogenase complex)	0	-0.67	-0.44	-0.55	down
SA0946	Q59822	Dihydrolipoyl dehydrogenase (EC 1.8.1.4)	0	-0.49	-0.52	-0.50	down
SA0969	Q7A661	SA0969 protein	1	0.20	0.34	0.27	up
SA0977	Q7A655	Cell surface protein	1	-0.48	-0.67	-0.58	down
SA1024	Q7A619	Penicillin-binding protein 1	1	0.20	0.27	0.23	up
SA1029	P45498	Cell division protein ftsZ	0	-0.38	-0.34	-0.36	down
SA1040	Q7A610	Pseudouridine synthase (EC 4.2.1.70) (Uracil hydrolyase)	0	0.28	0.31	0.30	up
SA1043	P65618	Aspartate carbamoyltransferase (EC 2.1.3.2) (Aspartate transcarbamylase) (ATCase)	0	-0.38	-0.45	-0.42	down
SA1045	P99147	Carbamoyl-phosphate synthase small chain (EC 6.3.5.5) (Carbamoyl-phosphate synthetase glutamine chain)	0	-0.85	-0.56	-0.70	down
SA1046	P63740	Carbamoyl-phosphate synthase large chain (EC 6.3.5.5) (Carbamoyl-phosphate synthetase ammonia chain)	0	-0.58	-0.55	-0.57	down
SA1053	P66726	DNA-directed RNA polymerase omega chain (EC 2.7.7.6) (RNAP omega subunit) (Transcriptase omega chain) (RNA polymerase omega subunit)	0	-0.26	-0.34	-0.30	down
SA1073	Q7A5Z3	Malonyl CoA-acyl carrier protein transacylase	0	-0.86	-0.42	-0.64	down
SA1133	Q7A5W4	Hypothetical protein SA1133	0	-0.42	-0.31	-0.36	down
SA1142	Q7A5V7	Aerobic glycerol-3-phosphate dehydrogenase	0	0.20	0.25	0.22	up

ORF number	AC	Description	TM domains	PE1:log(14-4/REV)	PE2:log(14-4/REV)	mean	expression
SA1170	Q7A5T2	Catalase (EC 1.11.1.6)	0	0.22	0.44	0.33	up
SA1195	Q99Q02	Peptide methionine sulfoxide reductase regulator MsrR	1	0.29	0.46	0.38	up
SA1238	P60108	TelA-like protein SA1238	0	0.21	0.37	0.29	up
SA1244	Q7A5N4	Dihydrolipoamide succinyltransferase	0	-0.89	-0.61	-0.75	down
SA1253	Q7A5M9	Probable carboxy-terminal processing proteinase ctpA	1	0.32	0.46	0.39	up
SA1256	P99065	Peptide methionine sulfoxide reductase msrB (EC 1.8.4.6)	0	0.23	0.39	0.31	up
SA1257	P65446	Peptide methionine sulfoxide reductase msrA 2 (EC 1.8.4.6) (Protein-methionine-S-oxide reductase 2) (Peptide Met(O) reductase 2)	0	0.45	0.47	0.46	up
SA1258	P67371	Hypothetical UPF0230 protein SA1258	0	-0.27	-0.29	-0.28	down
SA1271	Q7A5L8	SA1271 protein	0	-0.69	-0.60	-0.64	down
SA1279	Q7A5L1	Hypothetical protein SA1279	0	0.32	0.28	0.30	up
SA1283	Q7A5K8	PBP2	1	0.23	0.49	0.36	up
SA1293	Q7A5J9	Hypothetical protein SA1293	4	0.23	0.42	0.33	up
SA1307	P64060	GTP-binding protein engA	0	-0.48	-0.47	-0.47	down
SA1323	Q7A5H6	Staphylococcal respiratory response protein SrrA	0	-0.50	-0.29	-0.40	down
SA1361	Q7A5F0	Hypothetical protein SA1361	0	0.18	0.24	0.21	up
SA1454	Q7A591	Hypothetical protein SA1454	0	0.24	0.34	0.29	up
SA1476	Q7A580	Hypothetical protein SA1476	1	0.21	0.33	0.27	up
SA1506	P67585	Threonyl-tRNA synthetase (EC 6.1.1.3) (Threonine--tRNA ligase) (ThrRS)	0	-0.57	-0.51	-0.54	down
SA1517	P99167	Isocitrate dehydrogenase [NADP] (EC 1.1.1.42) (Oxalosuccinate decarboxylase) (IDH) (NADP+-specific ICDH) (IDP)	0	-0.42	-0.29	-0.35	down
SA1520	Q7A559	Pyruvate kinase	0	-0.42	-0.38	-0.40	down
SA1523	Q7A557	Acetyl-CoA carboxylase transferase beta subunit	0	0.28	0.24	0.26	up
SA1533	Q99TF2	Acetate kinase (EC 2.7.2.1) (Acetokinase)	0	-0.40	-0.44	-0.42	down
SA1571	P99090	D-alanine aminotransferase (EC 2.6.1.21) (D-aspartate aminotransferase) (D-amino acid aminotransferase) (D-amino acid transaminase) (DAAT)	0	-0.59	-0.37	-0.48	down
SA1593	Q99T94	Hypothetical protein SA1593	1	0.25	0.39	0.32	up
SA1653	Q7A4W3	Signal transduction protein TRAP	0	0.25	0.42	0.34	up
SA1654	Q7A4W2	SA1654 protein	10	0.27	0.23	0.25	up
SA1655	Q7A4W1	SA1655 protein	0	0.22	0.25	0.23	up
SA1659	P60748	Foldase protein prsA precursor (EC 5.2.1.8)	0	0.28	0.43	0.35	up

ORF number	AC	Description	TM domains	PE1:log(14-4/REV)	PE2:log(14-4/REV)	mean	expression
SA1691	Q7A4S6	SgtB protein	1	0.31	0.26	0.28	up
SA1701	Q99SZ7	Two-component sensor histidine kinase	2	0.24	0.55	0.40	up
SA1751	P69775	Map protein [Precursor]	1	0.29	0.24	0.27	up
SA1836	P99083	60 kDa chaperonin (Protein Cpn60) (groEL protein)	0	-0.31	-0.37	-0.34	down
SA1886	Q7A4F9	UDP-N-acetylmuramoylalanyl-D-glutamyl-2, 6-diaminopimelate-D-alanyl-D-alanyl ligase	0	0.17	0.26	0.22	up
SA1894	P66919	Thiamine-phosphate pyrophosphorylase (EC 2.5.1.3) (TMP pyrophosphorylase) (TMP-PPase) (Thiamine-phosphate synthase)	0	-0.43	-0.45	-0.44	down
SA1914	P67396	Uracil phosphoribosyltransferase (EC 2.4.2.9) (UMP pyrophosphorylase) (UPRTase)	0	-0.31	-0.41	-0.36	down
SA1936	P65330	S-ribosylhomocysteine lyase (EC 4.4.1.21) (Autoinducer-2 production protein luxS) (AI-2 synthesis protein)	0	-0.36	-0.30	-0.33	down
SA1938	Q7A4D0	Pyrimidine nucleoside phosphorylase	0	-0.29	-0.35	-0.32	down
SA2093	Q7A423	SsaA protein	0	0.22	0.40	0.31	up
SA2095	Q7A420	SA2095 protein	0	-0.37	-0.28	-0.33	down
SA2102	Q99RW4	SA2102 protein	0	0.26	0.40	0.33	up
SA2103	Q7A413	SA2103 protein	0	0.28	0.51	0.39	up
SA2106	Q7A410	SA2106 protein	7	0.27	0.33	0.30	up
SA2108	Q7A408	SA2108 protein	0	-0.46	-0.44	-0.45	down
SA2113	Q7A401	Hypothetical protein SA2113	0	0.33	0.34	0.34	up
SA2132	Q99RT2	SA2132 protein	0	-0.51	-0.37	-0.44	down
SA2221	Q7A3R1	Hypothetical protein SA2221	1	0.30	0.46	0.38	up
SA2237	Q7A3P8	Glycine betaine/carnitine/choline ABC transporter opuCA	0	0.19	0.44	0.32	up
SA2255	Q7A3N0	Oligopeptide transporter putative substrate binding domain	0	-0.51	-0.43	-0.47	down
SA2277	Q7A3K9	Hypothetical protein SA2277	0	-0.40	-0.31	-0.36	down
SA2302	Q7A3I7	SA2302 protein	0	-0.74	-0.47	-0.60	down
SA2312	P99116	D-lactate dehydrogenase (EC 1.1.1.28) (D-LDH) (D-specific D-2-hydroxyacid dehydrogenase)	0	-0.29	-0.69	-0.49	down
SA2336	Q7A3F4	ATP-dependent Clp proteinase chain clpL	0	0.20	0.25	0.22	up
SA2405	P60337	Choline dehydrogenase (EC 1.1.99.1) (CHD) (CDH)	0	0.24	0.48	0.36	up
SA2413	Q7A392	Sulfite reductase flavoprotein (EC 1.8.1.2)	0	0.27	0.35	0.31	up
SA2425	P99069	Carbamate kinase (EC 2.7.2.2)	0	-0.60	-0.57	-0.58	down
SA2428	P63554	Arginine deiminase (EC 3.5.3.6) (ADI) (Arginine dihydrolase) (AD)	0	-0.28	-0.32	-0.30	down

ORF number	AC	Description	TM domains	PE1:log(14-4/REV)	PE2:log(14-4/REV)	mean	expression
SAR0158	Q6GKE9	Capsular polysaccharide synthesis enzyme	0	0.25	0.46	0.35	up
SAS0971	Q6GAH7	Putative glycosyl transferases	0	-0.52	-0.93	-0.73	down
