# Supplementary material for: Exploring glycopeptide-resistance in Staphylococcus aureus: a combined proteomics and transcriptomics approach for the identification of resistance-related markers
Source: BMC Genomics. 2006 Nov 22;7:296. doi: 10.1186/1471-2164-7-296 (PMC1687195; doi:10.1186/1471-2164-7-296)
Supplement: Additional file 7 — Differentially expressed transcripts between 14-4 and MRGR3. Table showing all differentially expressed genes measured by using microarray between strains 14-4 (GISA) and MRGR3 (parental strain) [file 1471-2164-7-296-S7.pdf]

Additional file 7 : Differentially expressed transcripts between 14-4 and MRGR3

ORF number	14-4/MRGR3	Description	Expression
MW0353	3.348	hypothetical protein	up
MW0559	2.07	conserved hypothetical protein	up
MW0918	0.408	hypothetical protein	down
MW1600	2.309	hypothetical protein	up
MW1929	2.056	hypothetical protein	up
SA0041	2.851	xylose repressor homologue	up
SA0107	0.348166667	immunoglobulin G binding protein A precursor	down
SA0191	3.79	conserved hypothetical protein	up
SA0203	2.527	hypothetical protein	up
SA0227	0.367	conserved hypothetical protein	down
SA0272	0.5	transmembrane protein Tmp7	down
SA0274	0.0124	conserved hypothetical protein	down
SA0277	0.41	hypothetical protein	down
SA0338	0.487	hypothetical protein	down
SA0395	2.142	hypothetical protein	up
SA0452	2.121	VEG protein homologue	up
SA0467	0.498	conserved hypothetical protein	down
SA0532	22.46	hypothetical protein	up
SA0536	2.514	hypothetical protein	up
SA0536.1	2.054	vraX	up
SA0591	2.365	hypothetical protein	up
SA0653	0.456	transcription repressor of fructose operon	down
SA0688	0.395	ferrichrome ABC transporter permease	down
SA0689	0.336	ferrichrome ABC transporter permease	down
SA0797	2.274	nifU protein	up
SA0821	0.3285	argininosuccinate lyase	down
SA0825	2.381666667	type-I signal peptidase	up
SA0890	2.082	conserved hypothetical protein	up
SA0918	0.359333333	phosphoribosylaminoimidazolesuccinocarboxamide synthetase homolog	down
SA1041	0.476	pyrimidine operon repressor chainA	down
SA1067	2.354	50S ribosomal protein L28	up
SA1113	0.439	ribosome-binding factor A	down
SA1120	0.496	transcription regulator GntR family	down
SA1195	2.062	peptide methionine sulfoxide reductase regulator MsrR	up
SA1272	0.349	alanine dehydrogenase	down
SA1477	2.101	hypothetical protein	up
SA1493	0.475	uroporphyrinogen-III synthase	down
SA1621	0.343	hypothetical protein	down
SA1640	2.1	conserved hypothetical protein	up
SA1674	0.423	glutamate ABC transporter ATP-binding protein	down

ORF number	14-4/MRGR3	Description	Expression
SA1752	2.0435	truncated beta-hemolysin	up
SA1815	0.283	Na+-transporting ATP synthase	down
SA1835	0.468	integrase	down
SA1840	0.37	conserved hypothetical protein	down
SA1979	0.497	hypothetical protein, similar toferrichrome ABC transporter (binding protein)	down
SA1984	0.445	alkaline shock protein 23	down
SA2007	0.344	alpha-acetolactate decarboxylase	down
SA2091	0.497	hypothetical protein	down
SA2113	2.566	hypothetical protein	up
SA2146	2.824	tcaA protein	up
SA2303	0.3385	membrane spanning protein	down
SA2343	2.683	hypothetical protein	up
SA2412	2.227	uroporphyrin-III C-methyltransferase	up
SA2429	4.051	arginine repressor	up
SA2432	0.451	hypothetical protein	down
SA2480	2.5445	Drp35	up
SA2481	2.238	conserved hypotehtical protein	up
SACOL1582	0.218	conserved hypothetical protein	down
SACOL1583	0.11	conserved hypothetical protein	down
SACOL1586	0.161	conserved hypothetical protein	down
SACOL2187	3.094	hypothetical protein	up
SACOL2380	2.328	hypothetical protein	up
SACOL2558	3.579	hypothetical protein	up
SACOL2637	3.302	hypothetical protein	up
SAV0785	8.49	hypothetical protein	up
SAV0801	2.05	hypothetical protein	up
SAV0852	2.81	hypothetical protein	up
