# Supplementary material for: Exploring glycopeptide-resistance in Staphylococcus aureus: a combined proteomics and transcriptomics approach for the identification of resistance-related markers
Source: BMC Genomics. 2006 Nov 22;7:296. doi: 10.1186/1471-2164-7-296 (PMC1687195; doi:10.1186/1471-2164-7-296)
Supplement: Additional file 8 — Differentially expressed transcripts between 14-4 and 14-4Rev. Table showing all differentially expressed genes measured by using microarray between strains 14-4 (GISA) and 14-4Rev (susceptible revertant of GISA 14-4) [file 1471-2164-7-296-S8.pdf]

Additional file 8 : Differentially expressed transcripts between 14-4 and 14-4Rev

ORF number	14-4/14-4R	Description	Expression
MW0918	0.244	hypothetical protein	down
MW1195	2.446	hypothetical protein	up
MW1763	0.363	epidermin biosynthesis protein EpiC, authentic point mutation	down
SA0022	0.41	hypothetical protein, similar to 5'-nucleotidase	down
SA0041	2.691	xylose repressor homologue	up
SA0107	0.195	immunoglobulin G binding protein A precursor	down
SA0191	2.361	conserved hypothetical protein	up
SA0227	0.451	propionate CoA-transferase, putative	down
SA0519	0.275	Ser-Asp rich fibrinogen-binding, bone sialoprotein-binding protein	down
SA0532	10.41	hypothetical protein	up
SA0535	2.003	vra operon	up
SA0536	5.347	hypothetical protein	up
SA0536.1	4.613	vraX	up
SA0553	0.414	MesJ/Ycf62 family protein	down
SA0591	2.963	hypothetical protein	up
SA0603	0.491	ferrichrome transport permease	down
SA0688	0.314	similar to ferrichrome ABC transporter permease	down
SA0689	0.375	hypothetical protein	down
SA0821	0.198	argininosuccinate lyase	down
SA0825	2.231	type-I signal peptidase	up
SA0914	2.366	hypothetical protein, similar to chitinase B	up
SA0918	0.36	phosphoribosylaminoimidazolesuccinocarboxamide synthetase homolog	down
SA0977	0.499	cell surface protein	down
SA1009	0.402	hypothetical protein, similar to exotoxin 1	down
SA1067	2.118	50S ribosomal protein L28	up
SA1071	2.982	chitinase-related protein	up
SA1113	0.483	ribosome-binding factor A	down
SA1195	2.276	peptide methionine sulfoxide reductase regulator MsrR	up
SA1272	0.4	alanine dehydrogenase	down
SA1477	2.457	hypothetical protein	up
SA1586	2.161	6,7-dimethyl-8-ribityllumazine synthase	up
SA1621	0.304	hypothetical protein	down
SA1674	0.366	glutamate ABC transporter ATP-binding protein	down
SA1683	0.5	ABC transporter (ATP-binding protein) homolog	down
SA1691	2.465	similar to penicillin-binding protein 1A/1B	up
SA1700	2.076	two-component response regulator	up
SA1752	2.092	truncated beta-hemolysin	up
SA1810	0.415	integrase	down
SA1815	0.279	hypothetical protein, similar to Na+-transporting ATP synthase	down

ORF number	14-4/14-4R	Description	Expression
SA1816	2.034	extracellular enterotoxin L	up
SA1853	0.423	hypothetical protein, similar to DNA mismatch repair protein MutS	down
SA1959.1	2.44	hypothetical protein	up
SA1984	0.463	alkaline shock protein 23	down
SA2000	2.03	hypothetical protein	up
SA2007	0.378	alpha-acetolactate decarboxylase, putative	down
SA2083	2.011	urease beta subunit	up
SA2113	4.31	hypothetical protein	up
SA2146	4.232	tcaA protein	up
SA2156	2.043	SA2156	up
SA2303	0.291	hypothetical protein, similar to membrane spanning protein	down
SA2343	5.547	hypothetical protein	up
SA2412	2.505	similar to uroporphyrin-III C-methyltransferase	up
SA2429	4.23	hypothetical protein, similar to arginine repressor	up
SA2480	2.14	Drp35	up
SA2481	2.115	conserved hypotehtical protein	up
SACOL1043	0.173	glycosyl transferase	down
SACOL1336	2.446	hypothetical protein	up
SACOL2637	3.149	hypothetical protein	up
SAV0398	0.476	tetracycline resistance protein	down
SAV0785	5.265	hypothetical protein	up
SAV0801	2.656	hypothetical protein	up
SAV0852	2.269	hypothetical protein	up
SAV0859	5.096	hypothetical protein	up
