# Supplementary material for: Exploring glycopeptide-resistance in Staphylococcus aureus: a combined proteomics and transcriptomics approach for the identification of resistance-related markers
Source: BMC Genomics. 2006 Nov 22;7:296. doi: 10.1186/1471-2164-7-296 (PMC1687195; doi:10.1186/1471-2164-7-296)
Supplement: Additional file 9 — Evaluation of membrane purity by Western blot. Coomassie blue stained SDS-PAGE and Western blot performed with anti-protein A antibody allowing to evaluate enrichment in membrane proteins of the protein fractions. [file 1471-2164-7-296-S9.pdf]

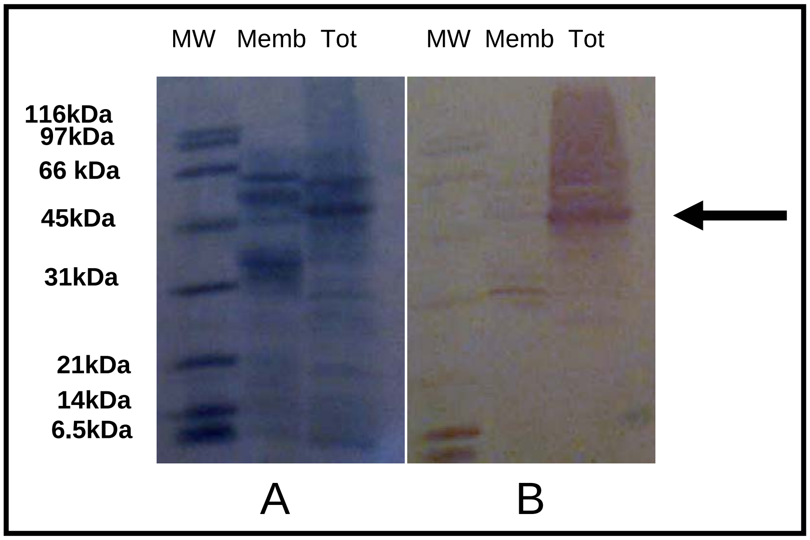

Additional file 9: Evaluation of membrane purity by Western blot. A. Coomassie stained gel loaded with 20 µg of protein fraction. **B.** Western blot performed with anti-spa antibodies (mab-spa-27from Sigma), revealed with anti-mouse Ig-phosphatase. An important signal around 50 kD is obtained from total protein extract whereas a very limited (but visible) signal was obtained from membrane preparation.
